# Mixed-ligand complexes of copper(ii) with thienoyltrifluoroacetonate and nitrogen containing ligands: synthesis, structures, antimicrobial activity, cytotoxicity, Hirshfeld surface analysis and DFT studies†

**DOI:** 10.1039/d2ra02428d

**Published:** 2022-08-17

**Authors:** Helen Oluwatola Omoregie, Oluwakemi A. Oloba-Whenu, Olawale J. Olowu, Tolulope M. Fasina, Alexandra Friedrich, Martin Haehnel, Todd B. Marder

**Affiliations:** Department of Chemistry, Faculty of Science, University of Ibadan Nigeria tolaomoregie@gmail.com; Department of Chemistry, Faculty of Science, University of Lagos Nigeria; Institute of Inorganic Chemistry, Julius-Maximilians-Universität Würzburg Am Hubland 97074 Würzburg Germany

## Abstract

Mixed-ligand complexes of copper(ii) with thienoyltrifluoroacetonate (TTA-H), 2,2′-bipyridine (bipy), 1,10-phenanthroline (phen), and tetramethylethylenediamine (tmen), associated with counter ions such as Cl^−^, and NO_3_^−^ have been synthesized and characterized by molar conductance measurements, elemental analysis, mass spectrometry, IR and UV-Vis spectroscopy, antimicrobial activity, cytotoxicity assay studies, and single-crystal X-ray diffraction. The UV-Vis spectra and crystal structures are consistent with the adoption of square pyramidal geometry for all of the complexes except [Cu(TTA)tmen]NO_3_ and [Cu(TTA)_2_tmen] which have square planar and octahedral geometries, respectively. Conductance measurements of the mixed-ligand complexes indicated that they were all non-electrolytes, with the ligands and anions being coordinated to Cu except [Cu(TTA)tmen]NO_3_ which is a 1 : 1 electrolyte. All of the complexes were moderately active on all the fungi tested (*Candida albicans*, *Aspergillus niger*, *Penicillium notatum*, *Rhizopus stolonifer*) except [Cu(TTA)bipyCl] which showed increased activity in *Candida albicans* and *Aspergillus niger.* All of the compounds tested showed LC_50_ values greater than 100 with [Cu(TTA)(phen)NO_3_] being the least toxic of the compounds. Molecular geometries of the complexes were optimized at the PBE1PBE/def2SVP and PBE1PBE/6-311g(d,p) level of theory and the results were compared with the single-crystal X-ray diffraction data. Electronic properties such as HOMO, LUMO, HOMO–LUMO gaps and global reactivity descriptors are reported at the PBE1PBE/6-311g(d,p) level of theory. Hirshfeld surface analysis was carried out to investigate the cooperative non-covalent supramolecular interactions within the various complexes.

## Abstract 2

Mixed-ligand complexes of copper(ii) with thienoyltrifluoroacetonate, 2,2′-bipyridine, 1,10-phenanthroline, and tetramethylethylenediamine, associated with counter ions such as Cl^−^ and NO_3_ have been synthesized and characterized.

## Graphical abstract

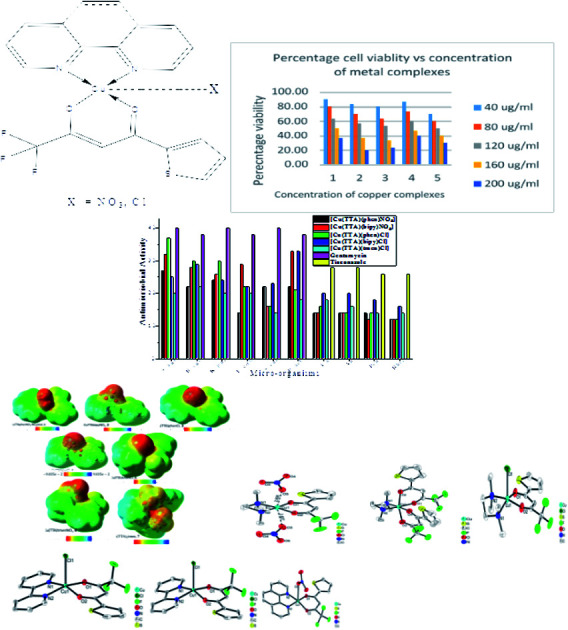

## Introduction

Metal ions play major roles in biological processes and the introduction of these ions into a biological system for the cure of diseases is one of the main subdivisions in the field of bioinorganic chemistry.^1^ Metal ions exist as electron-deficient cations in biological systems and are hence attracted to electron-rich biological molecules such as proteins and DNA.^2^ It has been reported that biological systems themselves provide numerous examples of ‘designer ligands’ that coordinate to metal ions to carry out important biological functions.^2^

Metal ions such as platinum, titanium, ruthenium, gold, copper, silver and their complexes have been found to show good biological activity such as antitumour, antiamoebic, antihistaminic, antiulcer, antimicrobial, anticancer and antihypertensive agents.^3–6^ Several transition metals are associated with many biological processes that are necessary for life.^7^ These transition metals can coordinate with C- or N- moieties from proteins in various ways, and they play an important role in the activity of biomacromolecules. These metal ions are currently present in various inorganic pharmaceuticals used as drugs against different types of diseases, ranging from antibacterial and antifungal agents to anticancer applications.^7–9^

Research has shown that free copper ions have toxic effects against both bacteria and fungi^10^ and, because of this behaviour, several researchers used the coordination of organic molecules to copper to improve the antimicrobial activity.^11,12^ Many copper-based complexes have been designed over the past few decades, possessing different kinds of ligands, substituents and geometries that influence their antimicrobial activities. These complexes showed promising action against microorganisms, dependent on their structural and electronic properties, and are relevant candidates for further pharmaceutical studies.^7,13,14^

Over the past decade, there has been considerable interest in the synthesis of metal complexes for potential application as drugs.^15^ Examples include the cleavage of DNA by copper complexes with 1,10-phenanthroline and 2,2′-bipyridine ligands to inhibit tumour cell growth.^16^ The small molecules that are being developed which are capable of catalyzing DNA hydrolysis under physiological conditions are very important in the development of novel drugs.^17^ Therefore, copper complexes have been studied as potential antitumor agents, as DNA is an important target for these drugs.^16,18,19^ The solvatochromic behaviour of mixed-ligand metal(ii) complexes has been extensively studied.^20–22^ The search for new synthetic routes to multimetallic complexes is of interest in modern coordination chemistry, and several versatile building blocks containing transition metal ions have been employed to generate heteropolynuclear complexes.^23^ Complexes of the type [Cu(AA)(BB)]X or [Cu(AA)(BB)X], where (AA) stands for acetylacetonate or salicylaldehydeate, (BB) = phen or me_2_bipy, and X = ClO_4_, were used as building blocks in the synthesis of mononuclear and heteropolynuclear complexes.^23^ Moreover, ternary copper(ii) complexes containing an aromatic diimine and a bidentate ligand with two oxygen atoms have been found to have rather unique qualities.^24^ Malekshah *et al.* reported biological studies and computational modeling of copper complexes derived from β-diketones and their nano-particles which were prepared by dissolving copper complexes in a small amount of *n*-hexane and placed in a high-density ultrasonic probe. This study showed that nano-particles have a stronger antiproliferative effect on MKN-45 cells than complexes.^25^ Furthermore, Malekshah *et al.* reported bioactivity and molecular simulation studies of four copper(ii) complexes with methylacetoacetonate and benzoylacetonate ligands. This study revealed that the human cancer cell lines are more sensitive to benzoylacetonate than methylacetoacetate complexes and the overall findings showed that the complexes are potential anticancer drugs.^26^ A comparative study of [Cu(TTA)bipyClO_4_] with carboplatin showed that the Cu complex has an IC_50_ lower than that of carboplatin, displaying a promising anticancer activity against K562 cells.^27^ Studies on mixed-chelate Cu(ii) complexes Casiopeínas® [Cu(TTA)(phen)(ClO_4_)] and [Cu(TTA)(phen)(N_3_)Cu(TTA)(phen)H_2_O](ClO_4_)H_2_O (TTA = 4,4,4-trifluoro-1-(2-furyl)-1,3-butanedionato) revealed that [Cu(TTA)(phen)(ClO_4_)] has a higher binding affinity to DNA than [Cu(TTA)(phen)(N_3_)Cu(TTA)(phen)H_2_O](ClO_4_)H_2_O and thus may be used as a substitute for anticancer drugs.^28^

While some studies have reported the anticancer activity of mixed-chelate Cu(ii) complexes [Cu(TTA)(bipy)(ClO_4_)]^27^ and [Cu(TTA)(phen)(ClO_4_)],^28^ the antimicrobial studies of the mixed ligand Cu(ii) complexes with thienoyltrifluoroacetonate (TTA-H), 1,10-phenanthroline (phen), 2,2′-bipyridine (bipy), and tetramethylethylenediamine (tmen), associated with counter ions such as Cl^−^ and NO_3_^−^ have not attracted attention. With the aim of investigating their cytotoxicity and antimicrobial activity, we have synthesized mixed ligand complexes of copper(ii) with thienoyltrifluoroacetonate, 1,10-phenanthroline, 2,2′-bipyridine, and tetramethylethylenediamine. Crystal structures, DFT optimized geometries, and Hirshfeld surface analyses of the compounds are also reported.

## Experimental

### Materials and physical measurements

The following reagents were used: thienoyltrifluoroacetonate (TTA-H) (Aldrich), copper nitrate trihydrate, copper chloride, tetramethylethylenediamine, 2,2′-bipyridine and 1,10-phenanthroline (Analytical grade). Elemental analyses for C, H, N were performed on an Elementar vario MICRO cube elemental analyzer at the Institute of Inorganic Chemistry at the Julius-Maximilians-Universität Würzburg. The % metals in the mixed-ligand complexes were determined by a titrimetric method with EDTA. The molar conductivities (*Λ*_m_) of the soluble compounds in nitromethane at room temperature were determined using a digital conductivity meter (Labtech). Infrared spectra of the complexes, as pressed KBr discs, were recorded on a Buck 500 Scientific model infrared spectrophotometer in the region 4000–400 cm^−1^. Absorption spectra of the complexes in chloroform and methanol solutions were recorded on a Spectro UV-VIS double beam PC scanning spectrophotometer UVD-2960. Antibacterial activity was determined by agar-cup diffusion as described by Singleton.^29^ HRMS measurements were carried out using a Thermo Fischer Exactive high-resolution mass spectrometer. Magnetic susceptibilities were measured with a Sherwood Scientific magnetic susceptibility balance, MSB Mark 1.

### Synthesis of [Cu(TTA)phenNO_3_](1)

Copper(ii) nitrate trihydrate (1.098 g, 4.54 mmol) dissolved in 1.8 mL water was added directly to a mixture of phenanthroline (0.9 g, 4.54 mmol) and thienoyltrifluoroacetonate (1.009 g, 4.54 mmol) in 10 mL of methanol. The mixture was stirred for 1 h and the precipitate formed was collected by filtration, washed with water and methanol, and dried *in vacuo*. The product was recrystallized by layering Et_2_O over methanol. Yield: 56.57%. *μ*_eff_ = 1.88 B.M. Anal. calc. for C_20_H_12_CuF_3_N_3_O_5_S: C, 45.59; H, 2.30; N, 7.97; S, 6.09; Cu, 12.06%. Found: C, 45.95; H, 2.38; N, 8.37; S, 5.84, Cu, 11.29%. IR [(*ν*(C=O) + *ν*(C=C)) 1603vs], [thienyl ring stretches 1542s, 1519s, 1430s, 1410s]; NO_2_ str, 1384s; N–O, 876m; *γ*(C–H), 858s, 723s. UV-Vis (CHCl_3_), *λ*_max_ (nm) = 665, 350, 275, 210, 205. Molar conductivity = 47 ohm^−1^ cm^2^ mol^−1^. HRMS (ASAP): calc. for [Cu(TTA)phen]^+^*m*/*z* 463.9868, found: 463.9853.

### Synthesis of [Cu(TTA)bipyNO_3_](2)

This complex was prepared using a similar procedure as for 1 with the following materials: copper(ii) nitrate trihydrate (1.098 g, 4.54 mmol) in water (10 mL); 2,2′-bipyridine (0.7092 g, 4.54 mmol) and thienoyltrifluoroacetonate (1.009 g, 4.54 mmol) in methanol (10 mL). Yield: 45.80%. *μ*_eff_ = 2.06 B.M. Anal. calc. for C_18_H_12_CuF_3_N_3_O_5_S: C, 42.99; H, 2.41; N, 8.36; S, 6.38; Cu, 12.64%. Found: C, 42.52; H, 2.30; N, 8.342; S, 6.35, Cu, 12.72%. IR [(*ν*(C=O) + *ν*(C=C)) 1637m, 1579w], [thienyl ring stretches 1542m, 1509m, 1476m, 1447m, 1408m]; NO_2_ str, 1384s; N–O, 826m; *γ*(C–H), 777s. UV-Vis (CHCl_3_), *λ*_max_ = 665, 310 nm, molar conductivity = 41 ohm^−1^ cm^2^ mol^−1^. HRMS (ASAP): calc. for [Cu(TTA)bipy]^+^*m*/*z* 439.9868, found: 439.9854.

### Synthesis of [Cu(TTA)phenCl] (3)

This complex was prepared using a similar procedure as for 1 with the following materials: copper(ii) chloride trihydrate (0.774 g, 4.54 mmol) in water (1 mL); 1,10-phenanthroline (0.900 g, 4.54 mmol) and thienoyltrifluoroacetonate (1.009 g, 4.54 mmol) in methanol (10 mL). Yield: 80.49%. *μ*_eff_ = 1.95 B.M. Anal. calc. for C_20_H_12_ClCuF_3_N_2_O_2_S: C, 48.01; H, 2.42; N, 5.60; S, 6.41; Cu, 12.70%. Found: C, 48.23; H, 2.23; N, 5.75; S, 6.15; Cu, 12.62%. IR [(*ν*(C=O) + *ν*(C=C)) 1629w], [thienyl ring stretches 1519vs, 1552m, 1531m, 1521m, 1513m, 1428vs, 1410vs]; *γ*(C–H), 847s, 721s. UV-Vis (CHCl_3_), *λ*_max_ = 760, 350, 296 nm. Molar conductivity = 34 ohm^−1^ cm^2^ mol^−1^. HRMS (ASAP): Calc. for [Cu(TTA)phen]^+^*m*/*z* 463.9868, found: 463.9854.

### Synthesis of [Cu(TTA)bipyCl] (4)

This complex was prepared using a similar procedure as for 1 with the following materials: copper(ii) chloride trihydrate (0.774 g, 4.54 mmol) in water (1 mL); 2,2′-bipyridine (0.7092 g, 4.54 mmol) and thienoyltrifluoroacetonate (1.009 g, 4.54 mmol) in methanol (10 mL). Yield: 65.19%. *μ*_eff_ = 2.12 B.M. Anal. calc. for C_18_H_12_ClCuF_3_N_2_O_2_S: C, 45.38; H, 2.54; N, 5.88; Cu, 13.34%. Found: C, 44.66; H, 2.46; N, 6.23; Cu, 13.01%. IR [(*ν*(C=O) + *ν*(C=C)) 1606m, 1567s], [thienyl ring stretches 1539s, 1513m, 1497s, 1476m, 1449s, 1408vs]; *γ*(C–H), 777s. UV-Vis (CHCl_3_), *λ*_max_ = 825, 350, 315, 300, 205 nm. Molar conductivity = 34 ohm^−1^ cm^2^ mol^−1^. HRMS (ASAP): calc. for [Cu(TTA)bipy]^+^*m*/*z* 439.9868, found: 439.9853.

### Synthesis of [Cu(TTA)tmenCl] (5)

This complex was prepared using a similar procedure as for 1 with the following materials: copper(ii) chloride trihydrate (0.3836 g, 2.25 mmol) in water (1 mL); *N*,*N*,*N*′,*N*′-tetramethylethylenediamine (0.3 mL, 2.25 mmol) and thienoyltrifluoroacetonate (0.5000 g, 2.250 mmol) in methanol (10 mL). Yield: 60.77%. *μ*_eff_ = 2.11 B.M. Anal. calc. for C_14_H_20_ClCuF_3_N_2_O_2_S: C, 38.53; H, 4.62; N, 6.42; S, 7.35; Cu, 14.65%. Found: C, 38.86; H, 4.70; N, 6.50; S, 7.47; Cu, 14.15%. IR [(*ν*(C=O) + *ν*(C=C)) 1693m, 1592vs], [thienyl ring stretches 1545s, 1511m, 1462m, 1412s]. UV-Vis (CHCl_3_), *λ*_max_ = 720, 354 nm. Molar conductivity = 38 ohm^−1^ cm^2^ mol^−1^.

### Synthesis of [Cu(TTA)tmen]NO_3_ (6)

This complex was prepared using a similar procedure as for 1 with the following materials: copper(ii) nitrate trihydrate (0.5436 g, 2.25 mmol) in water (1 mL) *N*,*N*,*N*′,*N*′-tetramethylethylenediamine (0.3 mL, 2.250 mmol) and thienoyltrifluoroacetonate (0.5000 g, 2.250 mmol) in methanol (10 mL). Yield: 50.33%. *μ*_eff_ = 1.92 B.M. Anal. calc. for C_14_H_20_CuF_3_N_3_O_5_S: C, 36.32; H, 4.35; N, 9.08; S, 6.93; Cu, 13.73%. Found: C, 36.40; H, 4.11; N, 8.92; S, 6.90; Cu, 13.75%. IR [(*ν*(C=O) + *ν*(C=C)) 1594s, 1553m], [thienyl ring stretches 1531w, 1519m, 1479s, 1412vs]; NO_2_ str, 1384s; N–O, 826w; UV-Vis (CHCl_3_), *λ*_max_ = 654, 306 nm. Molar conductivity = 67 ohm^−1^ cm^2^ mol^−1^.

### Synthesis of [Cu(TTA)_2_(tmen)] (7)

This complex was prepared using a similar procedure as for 1 with the following materials: copper(ii) acetate (0.4492 g, 2.25 mmol) in water (1 mL); *N*,*N*,*N*′,*N*′-tetramethylethylenediamine (0.3 mL, 2.25 mmol) and thienoyltrifluoroacetonate (0.5000 g, 2.25 mmol) in methanol (10 mL). Yield: 81.86%. *μ*_eff_ = 2.09 B.M. Anal. calc. for C_22_H_24_CuF_6_N_2_O_4_S_2_: C, 42.47; H, 3.89; N, 4.50; S, 10.31; Cu, 10.21%. Found: C, 42.73; H, 3.58; N, 4.79; S, 10.42; Cu, 10.81%. IR [(*ν*(C=O + (C=C)) 1622s, 1561w], [thienyl ring stretches 1531s]. UV-Vis (CHCl_3_), *λ*_max_ = 713, 353, 317 nm. Molar conductivity = 38 ohm^−1^ cm^2^ mol^−1^. HRMS (ASAP): Calc. for [Cu(TTA)(tmen)]^+^*m*/*z* 400.0494, found: 400.0479.

### Single-crystal X-ray diffraction

Crystals suitable for single-crystal X-ray diffraction were selected, coated in perfluoropolyether oil, and mounted on MiTeGen sample holders. Diffraction data were collected on a Bruker Smart-Apex (compounds 5, 6, 7) or a Bruker X8 Apex II (compounds 1, 2, 4) 4-circle diffractometer with a CCD area detector using graphite-monochromated Mo-K_α_ radiation. The crystals were cooled using a home-made nitrogen gas jet or Bruker Kryoflex low-temperature device. Data were collected at 168 K (5, 6, 7) or 100 K (1, 2, 4). The images were processed and corrected for Lorentz-polarization effects and absorption as implemented in the Bruker software packages. The structures were solved using the intrinsic phasing method (SHELXT)^30^ and Fourier expansion technique. All non-hydrogen atoms were refined in anisotropic approximation, with hydrogen atoms ‘riding’ in idealized positions, by full-matrix least squares against *F*^2^ of all data, using SHELXL software.^30^ Diamond^31^ software was used for graphical representation. Crystal data and experimental details are listed in Table S9 in the ESI;† full structural information has been deposited with Cambridge Crystallographic Data Centre. CCDC-2056492 (1), 2056493 (2), 2056494 (4), 2056495 (5), 2056496 (6), and 2056497 (7).

### *In vitro* antimicrobial studies

The antimicrobial activity of the synthesized compounds was determined by agar-cup diffusion method as described by Singleton. Each of the seven compounds was used at a concentration of 100 mg mL^−1^, prepared by dissolving 0.5 g in 5 mL of either sterile distilled water or methanol. Plate cultures were prepared by seeding method for bacteria and yeasts, and spread-plate method for mould using 0.1 mL of 10^−2^ dilution from 18-24 h broth culture of each bacterium in Mueller Hinton agar, and 24–72 h broth cultures of each fungus in Sabouraud dextrose agar. Each of the wells dug in the set agar media was filled with three drops of the dissolved compound (100 mg mL^−1^). A pre-incubation diffusion period of 1 h was allowed after which the culture plates were incubated at 37 °C for 24 h (for bacteria) and at 28 °C for 24–72 h (for fungi). Thereafter, the antimicrobial susceptibility was assessed by observing the plates for zones of growth inhibition which was measured in mm.^32,33^

### Cytotoxicity assay

The *in vitro* cytotoxic effects of the compounds were assayed by the brine shrimp lethality bioassay technique.^34,35^*Artemia salina* leach (brine shrimp eggs) was used as the test organism. The eggs were allowed to incubate for 48 h in a small tank containing sea water under constant illumination. The hatched larva (shrimp nauplii) were collected with a Pasteur pipette and diluted in fresh sea water to increase visibility. Stock solutions of 200 μg mL^−1^ of each compound were prepared in a 1 : 1 mixture of DMSO and seawater as solvent. The solutions were serially diluted to 160, 120, 80, 40 μg mL^−1^ with sea water. Then, 3 mL of the solution was added to 3 mL of sea water containing 10 nauplii in a test-tube. After 24 h, the test tubes were inspected with a magnifying glass and the number of nauplii which survived in each test tube was counted. Each assay was performed in triplicate. The median lethal concentration (LC_50_) was obtained using Probit analysis.

### Calculation details

The molecular geometries of the copper(ii) complexes were optimized using the Gaussian09W^36^ suite of software which includes GaussView.^37^ The structures were optimized using the hybrid density functional PBE1PBE^38–40^ with two different basis sets: the def2SVP,^36,37,41,42^ a split valence basis set with polarization on all atoms, and 6-311g(d,p),^43–45^ which is a Pople-type triple split valence basis set with polarization on all atoms. A subsequent frequency calculation at the same level of theory was carried out to confirm that the structures correspond to a minimum on the potential energy surface. The optimized structures are displayed using the visualization software CYLview Beta1.0,^46^ and the HOMO and LUMO and electrostatic potential map surfaces were generated using GaussView software.^37^ Calculated geometries of the title compounds were compared with experimental values obtained from crystallographic data. The geometry optimization was carried out using the coordinates from the experimentally determined crystal structures as the starting structures without constraints to bonds, angles or dihedral angles, and all atoms were free to optimize.

The Hirshfeld surfaces and 2D fingerprint plots were generated using Crystal Explorer 7.1 software.^47^ The crystallographic information file obtained from single-crystal X-ray diffraction was used as the input file. The TONTO application within Crystal Explorer was used to calculate the interaction energies using B3LYP/6-311G(d,p).

## Results and discussion

**Scheme 1:**
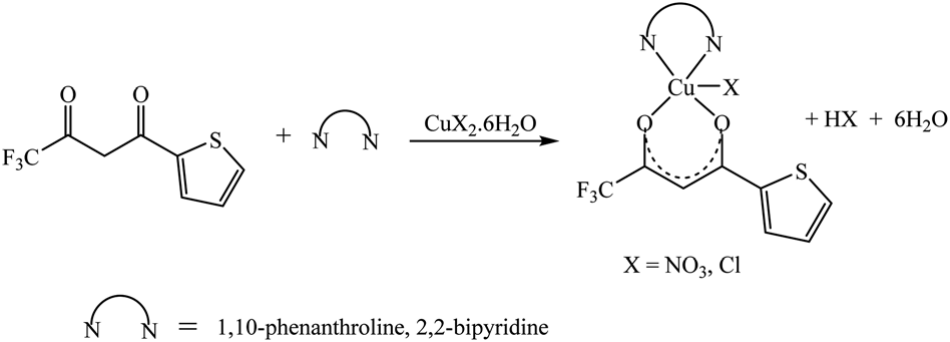


**Scheme 2:**
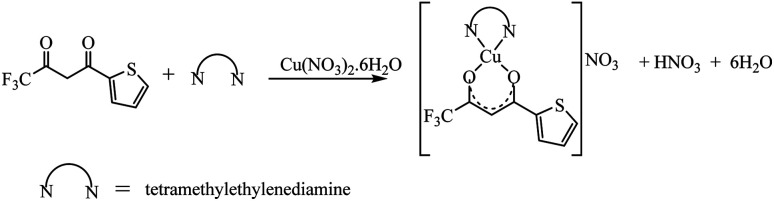


**Scheme 3:**
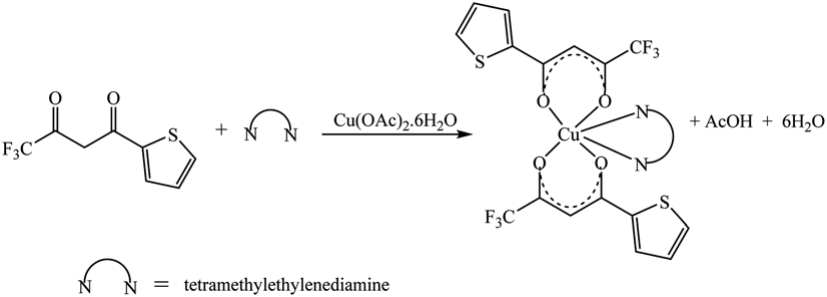


The synthetic methodology for complexes 1–7 is outlined in Scheme 1–3. The copper(ii) complexes were obtained by reaction of appropriate amounts of copper(ii) salts with thienoyltrifluoroacetonate and the respective ligand, 1,10-phenanthroline, 2,2′-bipyridine, or tetramethylethylenediamine, in good yields (46–81%). The formation of the complexes is given by the equation below:CuX_2_ + TTA–H + N–N → [Cu(TTA)(N–N)X] + HXwhere X = Cl^−^, NO_3_^−^ and N–N = 1,10-phenanthroline, 2,2′-bipyridine, or tetramethylethylenediamine.

All complexes were various shades of green in colour, except [Cu(TTA)(tmen)]NO_3_ which was dark blue in colour. The molar conductivities of the complexes are low with Λ_m_ values in the range of 34–47 ohm^−1^ cm^2^ mol^−1^, which suggest that they are non-electrolytes^48–50^ except [Cu(TTA)(tmen)]NO_3_ which is a 1 : 1 electrolyte.

### Single-crystal X-ray diffraction analysis

**Fig. 1:**
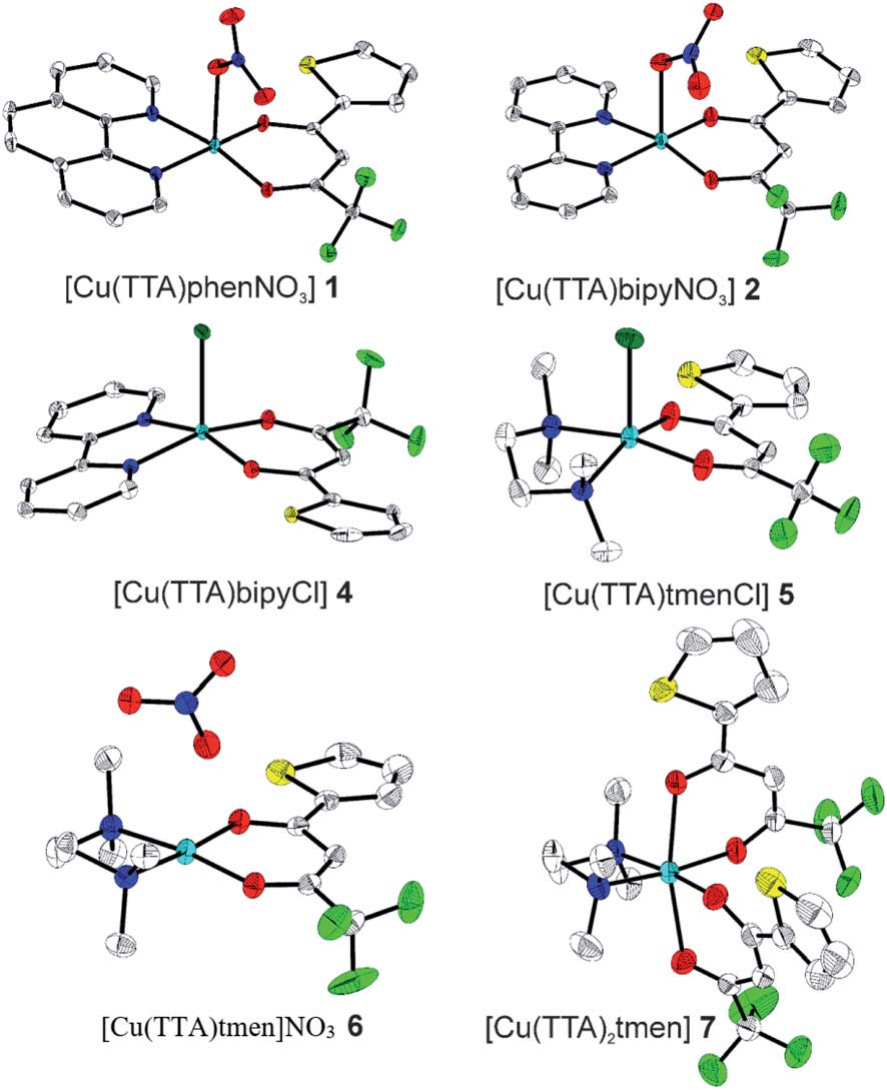
The solid-state molecular structures of 1, 2, 4, 5, 6 and 7, determined by single-crystal X-ray diffraction at 100 K. All ellipsoids are drawn at 50% probability, and H atoms as well as solvent molecules are omitted for clarity. Only the major parts of disordered molecules are shown. Atom colours: C (white), N (dark blue), O (red), F (light green), S (yellow), Cl (dark green), and Cu (light blue).

The crystal structures of compounds 1, 2, 4, 5, 6 and 7 were obtained using single-crystal X-ray diffraction, and their molecular structures are shown in Fig. 1. The central Cu atom of the complex has a square pyramidal [5]-coordination geometry in 1, 2, 4, and 5 with the nitrate or Cl anions in the apical position. The square is formed by two oxygen atoms of the TTA group and two nitrogen atoms of the phen, bipy, or tmen groups. In compound 6, Cu has a [4 + 2]-coordination geometry forming four short bonds with the N and O atoms of the tmen and TTA groups, respectively, in a square planar geometry and two strongly elongated apical contacts (2.6062(16) and 2.8265(18) Å, respectively, Table S10 and Fig. S15 in the ESI†) with oxygen atoms of two nitrate groups. In contrast, Cu is bonded to one tmen and two TTA groups in 7 and, hence, exhibits an octahedral [6]-coordination, which is slightly elongated due to the Jahn–Teller effect. These differences in the Cu coordination are also reflected in the bond angles. In the square pyramidal coordination, the Cu atom position is slightly offset from the square towards the apical atom with the O–Cu–N angles within the square (156.1–171.1° in 1, 2, 4 and 5, Table S10 in the ESI†) being significantly smaller than 180°. The offset of the Cu atom from the mean plane of the square is smaller in 1 and 2 (0.157(2) and 0.174(2) Å) with the NO_3_ anion in apical position than in 4 and 5 (0.305(1) and 0.320(1) Å) with the Cl anion. In the [4 + 2]- and [6]- coordinated compounds 6 and 7, however, the Cu offset is negligible (0.033(1) and 0.025(1) Å, respectively) and the O–Cu–N angles are close to 180° (172.7–175.5°, Table S10 in the ESI†) better representing a square planar or octahedral coordination. The N–N–O–O square is nearly planar with dihedral angles between *ca.* 0.6–7.5° (Table S10 in the ESI†). Within the equatorial square, the Cu–N bonds are shortest in the bipy group of compound 2 (1.975(4) and 1.978(4) Å), medium in the phen and bipy groups of 1 and 4 (1.996(3)–2.017(3) Å), respectively, longer in the tmen group of 6 (2.019(2) and 2.031(2) Å) and longest in the tmen groups of 5 and 7 (2.038(1)–2.075(1) Å) (Table S10 in the ESI†). This shows the elongating effect from bipy (2) to phen (1) to tmen (6) ligand, and when substituting NO_3_^−^ (2, 6) by Cl^−^ (4, 5), as well as from the increase in the coordination number ([6] in 7). The latter two effects are also observed for the Cu–O bonds, *i.e.*, Cu–O increases by substituting NO_3_^−^ (1, 2, 6, 1.918(4)–1.929(1) Å) with Cl^−^ (4, 5, 1.939(1)–1.963(1) Å), and is longest for [6]-coordination (7, 1.960(1) and 1.990(1) Å) (Table S10 in the ESI†). The Cu–O bonds towards the apical O atoms of the NO_3_ groups are elongated in 1, 2 and 6 (2.241(2)–2.292(1) Å) if compared to the Cu–O bonds within the square (1.918(4)–1.990(1) Å). The square pyramidal and octahedral geometries are also distorted by a significant tilt (4.95(5)–13.36(5)°) of the axial Cu–O bonds and contacts away from the normal of the mean square plane in 1, 2, 6 and 7, while the respective tilt, and hence distortion, is only small for the Cu–Cl bonds (2.35(4) and 1.47(3)°) in 4 and 5, respectively (Table S10 in the ESI†).

**Fig. 2:**
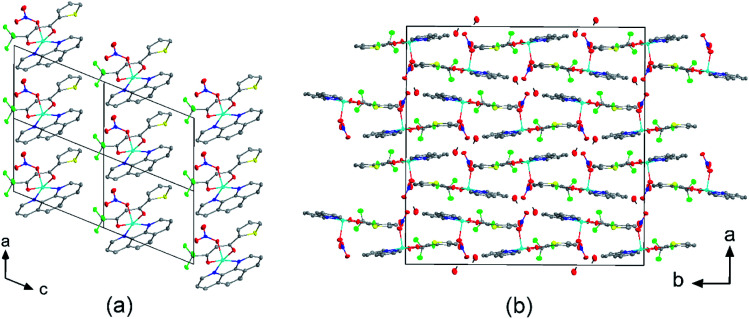
Crystal structures of (a) [Cu(TTA)phenNO_3_] (1) projected along the *b* axis, and (b) [Cu(TTA)bipyNO_3_] (2) projected along the *c* axis. (a) Four unit cells are shown. The complexes of 1 stack along the *a* axis by forming weak π⋯π interactions between phenanthroline and thienyl groups. (b) The complexes of 2 form dimers of antiparallel molecules with close Cu⋯Cu distances of 3.813(10) Å. Solvent water molecules form hydrogen bonds with the nitrate groups. Atom colours: C (white), N (dark blue), O (red), F (light green), S (yellow), Cl (dark green), and Cu (light blue).

**Fig. 3:**
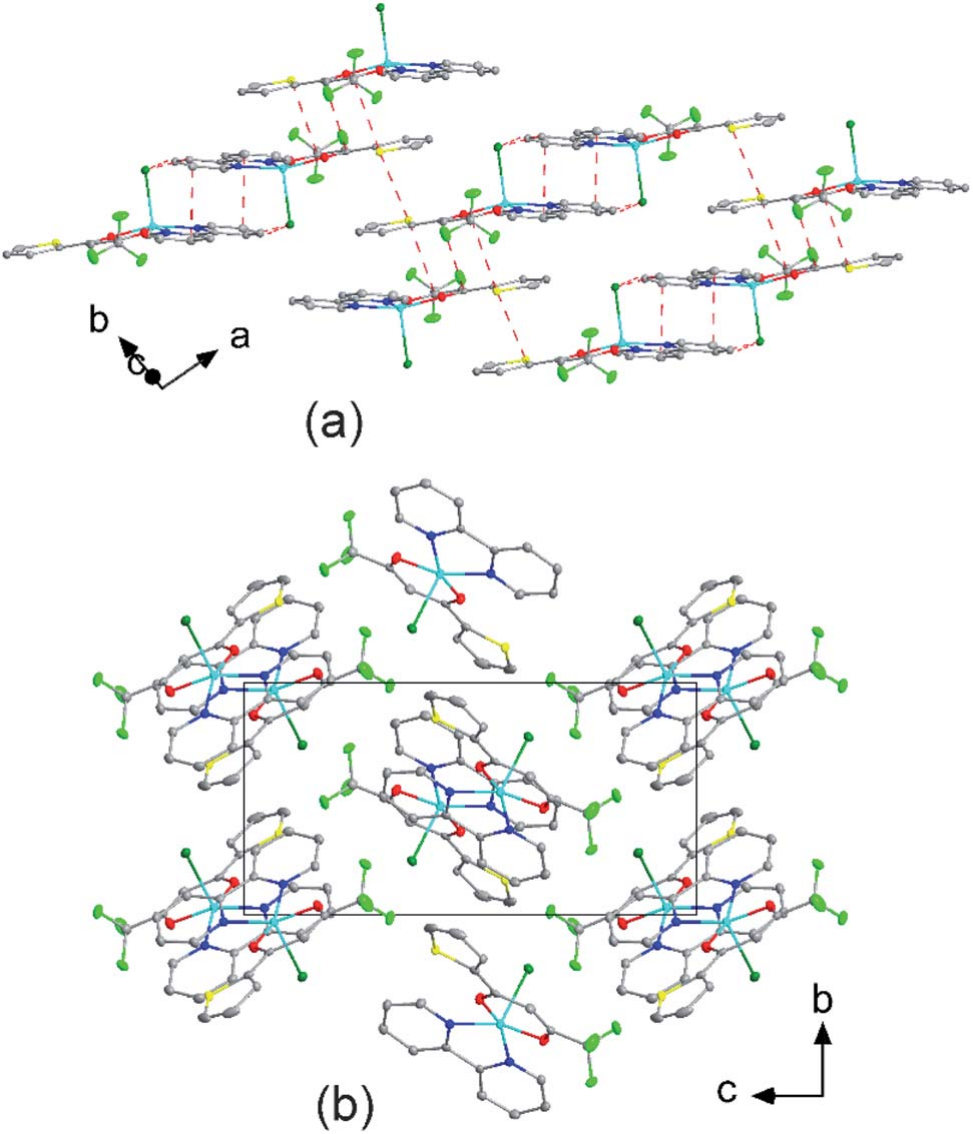
(a) Molecular packing showing the intermolecular interactions (red dashed lines) of [Cu(TTA)bipyCl] (4) and (b) crystal structure of 4 projected along the *a* axis. Atom colours: C (white), N (dark blue), O (red), F (light green), S (yellow), Cl (dark green), and Cu (light blue).

The molecular packing is particularly interesting in the crystal structures of compounds 1, 2 and 4, which contain planar phenanthroline or bipyridine ligands that form weak to moderate π-stacking interactions. In compound 1, all the phenanthroline ligands and, hence, complexes are oriented parallel to each other with the apical NO_3_ anions pointing towards the same direction (Fig. 2(a)). The complexes stack along the *a* axis by forming weak π⋯π interactions between phenanthroline and thienyl groups with mean interplanar separations of 3.52 Å and 3.45 Å (Table 1). In compound 2, the complexes form dimers of antiparallel molecules with close Cu⋯Cu distances of 3.813(10) Å (Fig. 2(b)). Dimers are interconnected *via* π⋯π interactions between the pyridine ring and the thienyl group with a mean interplanar separation of 3.38 Å and an angle of 9.20(18)° between plane normals (Table 1). The crystal structure of compound 2 also contains solvent water molecules, which form O–H⋯O hydrogen-bonds with nitrate groups. In compound 4, complexes are offset stacked along the *a* axis with alternating up and down orientation (Fig. 3). Within the stack, π⋯π-interactions between bipyridine ligands (interplanar separation of 3.3570(16) Å) alternate with intermolecular interactions between TTA ligands (mean interplanar separation of 3.34 Å; 6.01(7)° between plane normals), while Cl atoms form C–H⋯Cl bonds with the bipyridine units (Fig. 3(a)). Offset stacks are interconnected along the *b* axis *via* π⋯π-interactions between thienyl ligands (interplanar separation of 3.3499(18) Å) (Table 1).

**Table 1:** Aryl⋯aryl (π⋯π) distances (Å) and angles (°) in crystals of 1, 2 and 4: centroid–centroid distance, interplanar separation, offset shift, and slip angle. phen = phenanthroline, pyri = pyridine, bipy = bipyridine, thie = thienoylacetonate, tta = thienoyltrifluoroacetonate

Compound	Aryl⋯aryl	Centroid–centroid distance	Interplanar separation	Offset shift^a^	Angle between plane normals
1	phen⋯thie	4.9325(18)	3.502(8)/3.539(3)	3.473(8)/3.436(3)	1.91(12)
		3.578(2)	3.517(2)/3.510(3)	0.652(3)/0.693(7)	1.91(12)
2	pyri⋯thie	3.693(8)	3.416(10)/3.352(9)	1.551(8)	9.20(18)
4	bipy⋯bipy	3.5548(17)	3.3570(16)	1.169(3)	0.0(2)
	thie⋯thie	3.711(2)	3.3499(18)	1.597(5)	0.0(3)
	tta⋯tta	3.722(2)	3.324(2)/3.4670(17)	1.673(4)/1.353(3)	6.01(7)

a The offset shift, also called inter-centroid shift, is the distance within a plane of an aryl ring between the centroid of the respective aryl ring and the intersection point with the normal to the plane through the centroid of the other aryl ring.

### Hirshfeld surface analysis

**Fig. 4:**
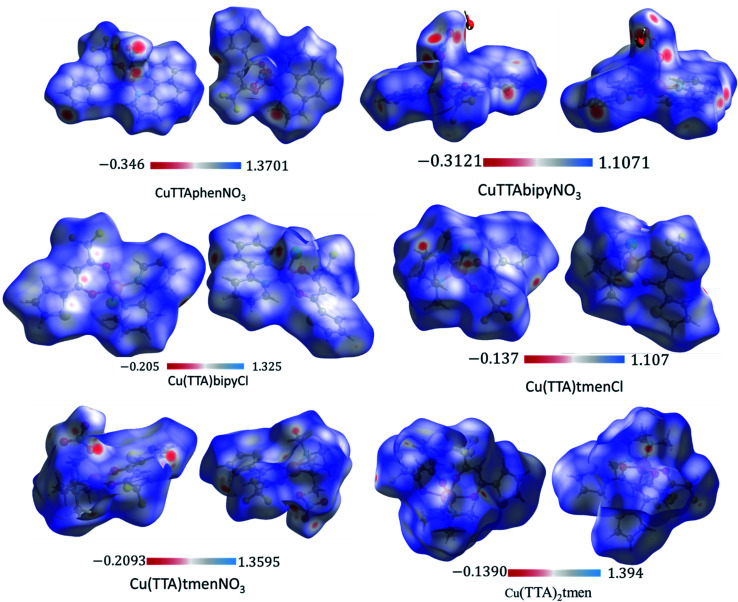
3D Hirshfeld surfaces of 1, 2, 4, 5, 6 and 7 plotted in the indicated range in a.u.

**Fig. 5:**
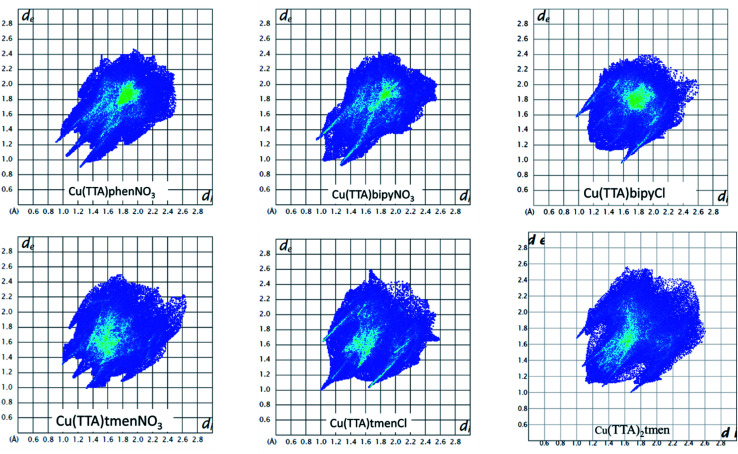
Fingerprint plots as obtained from the crystal structures of all the six complexes.

The Hirshfeld surface was analyzed to further characterize the nature and amount of intermolecular contacts. A Hirshfeld surface is defined by the density weight function of the specific molecule of interest over the same sum of density of its nearest neighbour, thereby resulting in a 0.5 arbitrary units isosurface. It is similar to that of a van der Waals surface but takes into consideration neighbouring molecules and, hence, provides information about intermolecular interactions.^51,52^ The Hirshfeld surface can be mapped over properties such as *d*_norm_, *d*_i_, *d*_e_, curvature, shape index^53^ and electrostatic potential.^54^ The normalized contact distance *d*_norm_, based on both *d*_e_ (distance from the point to the nearest nucleus external to the surface) and *d*_i_ (distance to the nearest nucleus internal to the surface) and also the van der Waals (vdW) radii of the atoms, enables identification of the regions of particular importance to intermolecular interactions.^52^ The Hirshfeld surfaces mapped over *d*_norm_, in the range indicated to each complex, are displayed for the complexes 1, 2, 4, 5, 6 and 7 enveloping the molecular structures in Fig. 4. The *d*_norm_ values are mapped onto the Hirshfeld surface using a red–blue–white colour scheme: red regions correspond to closer contacts and negative *d*_norm_ value, the blue regions correspond to longer contacts and positive *d*_norm_ value and the white regions are those where the distance of contacts is exactly the vdW separation and with a *d*_norm_ value of zero. The combination of *d*_e_ and *d*_i_ in the form of a 2D fingerprint plot^55^ provides the summary of intermolecular contacts in the crystal and significant intermolecular interactions are mapped in Fig. 5. The percentage values calculated for all possible intermolecular contacts are given in Table S8.† Full decomposed fingerprint plots are given in the ESI.† In all cases, the grey shadow is an outline of the complete fingerprint plot (Fig. S4–S9†). In all complexes, the H⋯H, H⋯F/F⋯H, and H⋯C/C⋯H interactions are the most dominant. In the Cl complexes, H⋯Cl/Cl⋯H interactions are dominant and H⋯O/O⋯H are important in the nitro complexes. A significant amount of C⋯C interactions, which mostly indicate π–π stacking, is present in complexes 1(12%), 2(8%) and 4(5.6%) which contain the planar 1,10-phenanthroline or 2,2′-bipyridine ligands and are insignificant or absent in others. The H⋯C/C⋯H interaction is least in 1 which has the highest amount of C⋯C interaction. The C⋯F/F⋯C and H⋯S/S⋯H interactions are sizeable in all the complexes. For 7, only the H⋯H, H⋯F/F⋯H and H⋯C/C⋯H interactions are important.

Curvedness is a function of the root-mean-square curvature of the surface, with flat areas of the surface having a low curvedness and areas of sharp curvature having a high curvedness.^51^ A surface with low curvedness designates a flat region and may be indicative of π–π stacking in the crystal. On the other hand, a Hirshfeld surface with high curvature is highlighted as dark-blue edges, which is indicative of an absence of π–π stacking and usually tends to divide the surface into patches, indicating interactions between neighbouring molecules. The Hirshfeld surface mapped over the curvature of the complexes are given in Fig. S10 in the ESI.†

### DFT calculated optimized geometries

**Fig. 6:**
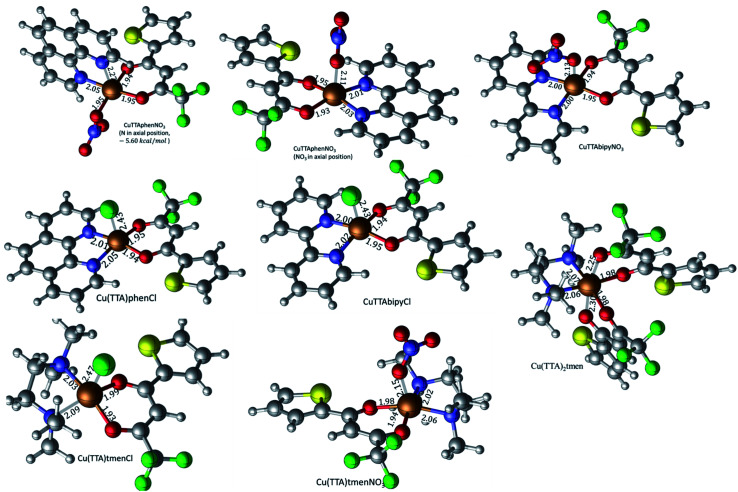
Geometry-optimized molecular structures of the title complexes starting from the experimentally determined crystal structures at the PBE1PBE/6-311g(d,p) level of theory. Selected bond lengths (Å) are shown.

**Fig. 7:**
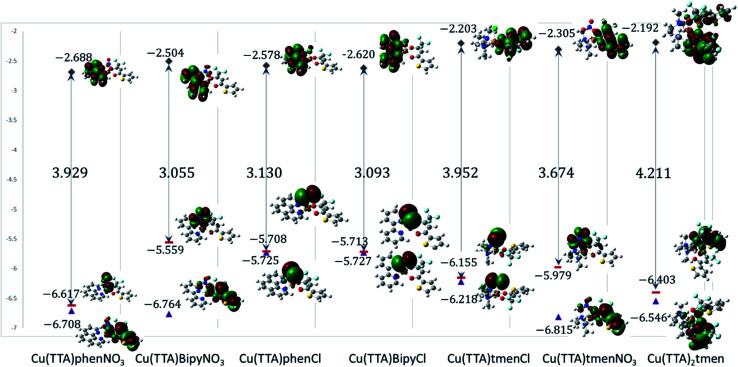
Frontier molecular orbitals of the complexes. The calculated energies are given in eV.

The isolated molecular geometries were optimized using the PBE1PBE functional and 6-311g(d,p) basis set and the molecular structures obtained from X-ray diffraction as the starting structures. The optimized structures are displayed in Fig. 6. A comparison between the optimized and experimentally determined bond lengths and angles are given in Tables S2–S7 in the ESI.† Complex 7 yielded the expected octahedral complex in good agreement with the experimentally determined crystal structure, and complexes 1 to 6 yielded the expected square pyramidal structures. However, for the square pyramidal complexes, optimization with the 6-311g(d,p) basis set yielded structures with either Cl or NO_3_ in the axial position as in the X-ray structure, except for [Cu(TTA)phenNO_3_], 1 where one of the nitrogen atoms of the phenanthroline was in the axial position. When the NO_3_ ligand was constrained to be in the axial position, the structure obtained was calculated to be about 5.6 kcal mol^−1^ less stable than the initially obtained structure; this is also displayed in Fig. 7. Optimization using the basis set def2svp yielded structures where either the oxygen atom of TTA ligand or the nitrogen atom of the corresponding amine/imine ligand is in the axial position, Fig. S1.† Because of the results obtained using the def2svp basis set, structures with either the oxygen atom of the TTA or nitrogen atom of the corresponding amine were optimized with the 6-311g(d,p) basis set and these are displayed in Fig. S2,† and their corresponding energies with respect to the structures in Fig. 7 are also given. It is observed that they are calculated to be lower in energy than when either the Cl or NO_3_ was in the axial position. However, the energy difference was not as large as that which was observed in the [Cu(TTA)phenNO_3_] complex. With the Cl ligand, optimization with the nitrogen atom in the axial position was not observed with either the 6-311g(d,p) or dev2svp basis sets. We believe that Cl or NO_3_ in the axial position in the crystal structure must be due to other intermolecular interactions in the crystal.

In all cases, however, with the 6-311g(d,p) basis, bond lengths are in good agreement with the experimental structures, but there are obvious differences in the dihedral angles, (see Tables S2–S7 in the ESI†). It should also be noted that these differences in bond parameters are attributed to the fact that the experimental results are in the solid state whereas the theoretical calculations were carried out in the gas phase.

For 1, results discussed below are based on the NO_3_ in the axial position as this was what was observed in the crystal structure determined by X-ray diffraction.

### Electronic properties and frontier molecular orbital analysis

Frontier molecular orbital energy studies can provide valuable insights into biologically active compounds' potential biological mechanisms.^56^ The values of the calculated energies of the HOMO, *E*_HOMO_, energy of the LUMO, *E*_LUMO_, energy gap Δ*E*_gap_, ionization energy (*I*), and electron affinity (*A*)^57,58^ are presented in Table 2. These are also represented in Fig. 7 with the electron density distribution plot of the LUMO, HOMO and HOMO-1. It is observed that the ease of release of electrons will therefore probably follow the trend 1 < 7 < 5 < 6 < 4 < 3 < 2 and ease of accepting electron will follow the trend 7 < 5 < 6 < 2 < 3 < 4 < 1. From Table 2 the [Cu(TTA)bipyNO_3_] complex 2 has smallest energy gap with Δ*E*_gap_ = 3.055 eV and the [Cu(TTA)_2_tmen] complex 7 has the largest energy gap Δ*E*_gap_ = 4.211 eV. The trend in the Δ*E*_gap_ follows 2 < 4 < 3 < 6 < 1 < 5 < 7. All of the metal complexes, except [Cu(TTA)_2_tmen], also exhibit large dipole moments, which may favour their interactions with other species with large dipole moments *via* dipole–dipole interactions, especially in biological systems. The square pyramidal complexes are more polar than the octahedral complexes.

**Table 2:** Energies of the HOMO, LUMO and some global reactivity descriptors^a^

	[Cu(TTA) phenNO_3_] 1	[Cu(TTA) bipyNO_3_] 2	[Cu(TTA)phenCl] 3	[Cu(TTA) bipyCl] 4	[Cu(TTA)tmenCl] 5	[Cu(TTA)tmenNO_3_] 6	[Cu(TTA)_2_ tmen] 7
*E*_HOMO_ (eV)	−6.617	−5.559	−5.708	−5.713	−6.155	−5.979	−6.403
*E*_LUMO_ (eV)	−2.688	−2.504	−2.578	−2.620	−2.203	−2.305	−2.192
Δ*E*_gap_ (eV)	3.929	3.055	3.130	3.093	3.952	3.674	4.211
*I* (eV)	6.617	5.559	5.708	5.713	6.155	5.979	6.403
*A* (eV)	2.688	2.504	2.578	2.620	2.203	2.305	2.192
*η* (eV)	1.965	1.528	1.565	1.547	1.976	1.837	2.106
*μ* (eV)	−4.653	−4.032	−4.143	−4.167	−4.179	−4.142	−4.298
*χ* (eV)	4.653	4.032	4.143	4.167	4.179	4.142	4.298
*ω* (eV)	21.26	12.41	13.43	13.42	17.25	15.76	19.44
*S* (eV)	−0.255	−0.327	−0.319	−0.323	−0.253	−0.272	−0.237
Dipole moment	10.7	9.3	10.1	10.0	9.2	10.2	8.1

a Key: chemical hardness: *η*, (eV); chemical potential: *μ*, (eV); electronegativity: *χ*, (eV); global softness: *S*, (eV); Δ*E*_gap_ = *E*_LUMO_ − *E*_HOMO_, (eV); electrophilicity index: *ω*, (eV).

From Fig. 7, for the square pyramidal complexes, the HOMO (SOMO) density is mainly on the p-orbital of either Cl/NO_3_ bonded with the d-orbital on the copper atom. The density of the HOMO-1, which is the highest doubly occupied molecular orbital, varies; for the chloride complexes, the electron density is mainly on the Cl p-orbital bonded to the d-orbital of the central atom and, for the NO_3_ complexes, a sizable contribution from the TTA ligand is observed in addition to the charge density contribution from the NO_3_ ligand and copper atom. The LUMO is mainly on the phenanthroline and bipyridine ligands; however, in complexes with the tetramethylethylenediamine, the LUMO is on the TTA ligand. The implication of this is that the metal complexes studied will preferably interact with electrophilic species through the axial Cl and NO_3_ ligand in the square pyramidal complexes and through the TTA ligand in the octahedral complex. They will interact with nucleophilic species through the TTA ligand in 5, 6 and 7 and through phenanthroline and bipyridine ligands in the other complexes. Also, metal-to-ligand charge transfer transitions are expected. For the octahedral complex, 7, the HOMO is a copper d-orbital spread over one of the TTA ligands and the LUMO is mainly on the other TTA ligand.

Global reactivity descriptors such as chemical hardness (*η*), electronic chemical potential (*μ*), electronegativity (*χ*), global softness (*S*), and electrophilicity index (*ω*), were also considered and calculated at the PBE1PBE/6-311g(d,p) level of theory. The chemical potential, *μ*, and chemical hardness, *η*, are defined as the first derivative of the electronic energy and chemical potential with respect to the electron number (*N*) at constant external potential, *v*(*r*), respectively, as given in eqn (1) and (2)1
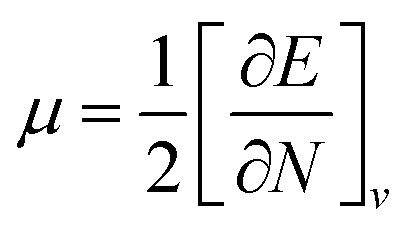
2
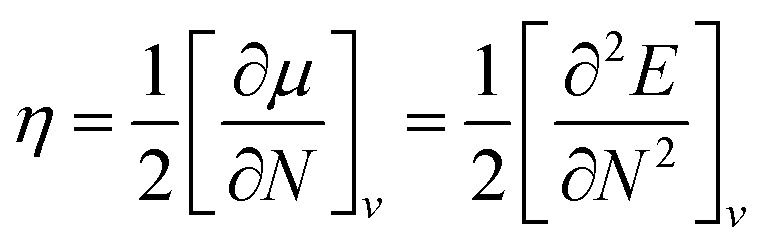


The chemical potential, *μ* characterizes the escaping tendency of electrons from the equilibrium system and the molecular hardness determines the resistance to charge transfer. Within the framework of the finite differences approximation, the above descriptors and other global descriptors can also be calculated as follows.^57–60^3
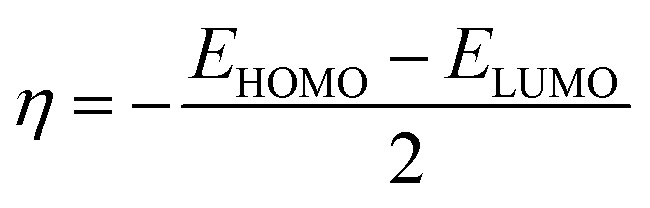
4
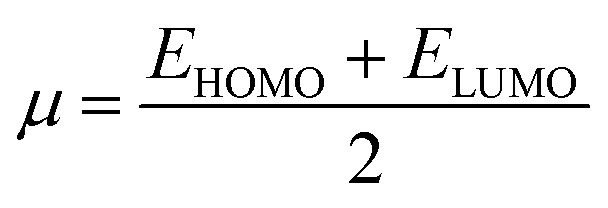
5
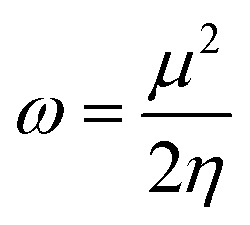
6
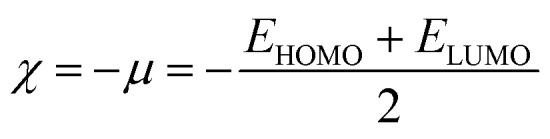

7 *S* = 1/2*η*

The values of all important reactivity descriptors of the compounds under study are given in Table 2. It is clear that among all derivatives, the octahedral complex has the highest value of *η* equal to 2.106 eV being the chemically hardest compound among all, and [Cu(TTA)bipyNO_3_], 2, has the lowest value (1.528 eV). The complexes with the bipyridine ligand are softer than the others. From Table 2, it is clear that [Cu(TTA)phenNO_3_], 1, has the lowest chemical potential value (−4.653 eV), while [Cu(TTA)bipyNO_3_], 2, has the highest chemical potential −4.032 eV). The electrophilicity index (*ω*) is a thermodynamic property that measures the changes in energy when a chemical system becomes saturated by adding electrons. The results from Table 2 indicate that [Cu(TTA)phenNO_3_], 1, has the highest electrophilicity index value (21.26 eV) and is the most electrophilic in nature, whereas [Cu(TTA)bipyNO_3_], 2, has the lowest value of 12.41 eV and is the least electrophilic in nature and others follow the trend 4 ≈ 3 < 6 < 5 < 7. The electronegativity (*χ*) trend is 2 < 6 ≈ 3 < 4 < 5 < 7 < 1.

The electrostatic potential surface (ESP),^61^ is given in Fig. S3† for all of the complexes studied. The ESP surface is positive around the hydrogen atoms surrounding the amine ligand and the negative charge is mostly around the corresponding Cl/NO_3_ ligand in the square pyramid.

### Electronic spectra

The electronic solution spectra of the mixed-ligand complexes of copper(ii) were studied in chloroform and methanol and their tentative assignments are presented in Table 3. The electronic properties of copper(ii) β-diketonates in solution are well known to be strongly dependent on the solution media and coordinating solvents are found to have a particularly dramatic influence on it.^62,63^ It is well known that the d–d absorption spectra of copper(ii) β-diketonates are much influenced by the variation of solvents and that the positions and intensities of the spectra depend primarily upon the solvent basicity.^64^ The stronger ligation to the copper(ii) ion along the tetragonal axis causes an appreciable shift of the d–d absorption band to lower energy and results in an increase of the intensity of the band.^62,64^ Hence, a minor shift to higher frequency of the ligand field spectral band in a coordinating solvent such as methanol relative to a non-coordinating solvent for copper(ii) species in solution is indicative of a probable transformation from five-coordinate square pyramidal copper(ii) environment to six-coordinate environment upon coordination of such solvents as methanol.^49,50,63^ Thus, complexes 1–5 have square–pyramidal geometry due to higher energy shifts in methanol relative to chloroform. The intraligand π–π* and ligand to metal charge transfer (LMCT) transitions are observed in the 28 249–50 000 cm^−1^ region for the synthesized complexes.^65,66^

**Table 3:** Relevant electronic solution spectra of mixed-ligand complexes of copper(ii) with thienoyltrifluoroacetonate and nitrogen containing ligands

Compounds	Charge transfer/π–π^a^ (cm^−1^) (ε)						d–d (cm^−1^) (ε)	
	CHCl_3_	CH_3_OH	CHCl_3_	CH_3_OH	CHCl_3_	CH_3_OH	CHCl_3_	CH_3_OH
TTA-H			31 056 37 073 bsh	34 247 (1711) 37 073 bsh	28 409	29 586 (2335)	—	—
[Cu(TTA)phen(Cl)]	—	47 619 (40 829)	33 809 (29 756)	—	28 571 (21 382)	28 571 (23 630)	13 158	15 625
[Cu(TTA)phenNO_3_]	48 780 (429)	47 619 (36 966)	36 364 (32 634)	36 364 (32 266)	28 571 (16 031)	—	15 038	15 625
[Cu(TTA)bipyNO_3_]	—	—	32 258 (8020)	—	—	—	15 038	15 748
[Cu(TTA)bipy(Cl)]	48 780 (634)	—	31 711 (1099)	32 258 (18 320)	28 571 (871)	28 571 (17 924)	12 121	15 625
			33 333 (1188)					
[Cu(TTA)tmen]NO_3_	—	—	32 680 (14 271)	—	—	28 986 (44 282)	15 291	—
[Cu(TTA)tmenCl]	—	—		33 233 (2378)	28 249	—	13 888 (229)	16 393
[Cu(TTA)_2_tmen]		—	31 546 (15 121)	—	28 329	—	14 025	—

a — Shoulder.

The solid state electronic reflectance spectra of the copper(ii) complexes were carried out in nujol and their tentative assignments are presented in Table 4. The assignments of bands were made by reference to similar compounds in the literature. A single broad band was observed in the visible spectra of complexes 1–5 in the range 16 667–14 124 cm^−1^ which is consistent with a square-pyramidal geometry for copper(ii) complexes.^67^ The ultraviolet spectra of the compounds are characterized by three or four peaks between 27 624–48 780 cm^−1^. These bands have been attributed to charge transfer transitions and intraligand π–π* transitions.^65,66^ Calculated absorption UV-visible spectra from TD-DFT at the m06-2x/6-311++G(d,p) level of theory was within the ranges from 26 116 to 8465 cm^−1^ (382 to 1183 nm) for all complexes (Table S12†). The square pyramidal complexes with the tmen ligand and the octahedral complex gave rise to a band around 25 000 cm^−1^ (400 nm). The other complexes displayed more prominent peaks at lower energies of *ca.* 20 790–19 084 cm^−1^ (481–524 nm), but with higher intensity. The prominent bands correspond mainly to d–d transitions with some ligand-to-ligand charge transfer. These values are slightly higher than the experimental values. However, they are qualitatively in agreement in the relative order observed experimentally, although the calculations were performed in vacuum.

**Table 4:** Electronic solid state reflectance spectra of copper(ii) mixed ligand complexes of thenoyltrifluoroacetonate with 1,10-phenanthroline and 2,2′-bipyridine

	d–d transitions cm^−1 ^	CT/π–π* transitions
[Cu(TTA)phenNO_3_]	14 080	35 007, 40 107, 43 558, 48 780
[Cu(TTA)bipyNO_3_]	15 594	35 248, 41 167
[Cu(TTA)phenCl]	15 408	29 972, 34 934, 41 865, 47 393
[Cu(TTA)bipyCl]	16 667	35 842, 44 843, 46 728, 48 780
[Cu(TTA)tmenCl]	14 124	27 624, 31 447, 39 526
[Cu(TTA)_2_(tmen)]	14 025	27 624, 31 646, 39 526
[Cu(TTA)tmen]NO_3_	17 421	27 624, 31646, 39 841

### Cytotoxicity assay

**Fig. 8:**
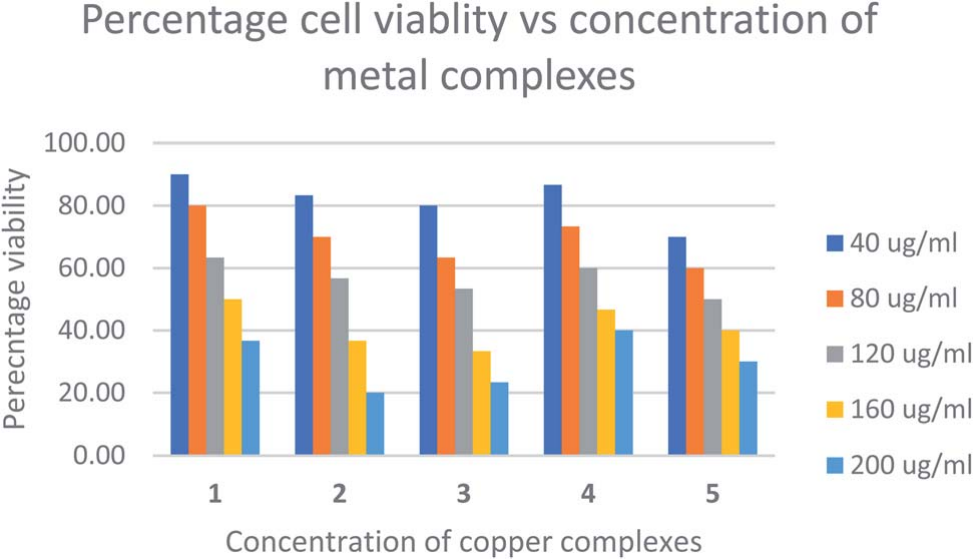
Percentage cell viability *vs.* concentration of metal complexes.

The relative cytotoxicity of the compounds was evaluated using the brine shrimp assay. The percentage of viable eggs relative to the standard were plotted against the concentration of the compounds and the LC_50_ values obtained using Probit analysis. The dose-dependent cytotoxic activity of the complexes is shown in Fig. 8. Compounds with LC_50_ values in range 100–500 ppm are considered to have moderate cytotoxicity while those with LC_50_ ≥ 1000 are non-toxic.^67^ All compounds tested showed LC_50_ values > 100μg mL^−1^ (Table S1†) indicating the compounds are moderately toxic.

The toxicities at 160 μg mL^−1^ of [Cu(TTA)phenNO_3_](1) [Cu(TTA)bipyNO_3_] (2) [Cu(TTA)phenCl] (3) [Cu(TTA)bipyCl] (4) and [Cu(TTA)tmenCl] (5) were found to be 50, 37, 33, 47, and 40%, respectively. These results reveal that [Cu(TTA)phenNO_3_] has the lowest cytotoxic activity amongst the complexes studied. The LC_50_ values indicate that both the nature of the bidentate chelating and anionic ligands affect the toxicity of the compounds. Previous studies^68^ indicate that copper complexes containing 1,10 phenanthroline have lower LC_50_ values compared to 2,2′-bipyridine presumably due to separate actions of Cu(ii) ions and the phen ligand. However, in this study [Cu(TTA)phenNO_3_](1), bearing both phen and NO_3_ exhibited lowest cytotoxicity. The toxicity of the compounds containing a chloride ligand varied with the bidentate ligand in the order bipy < phen < tmen.

### Antimicrobial activity

**Fig. 9:**
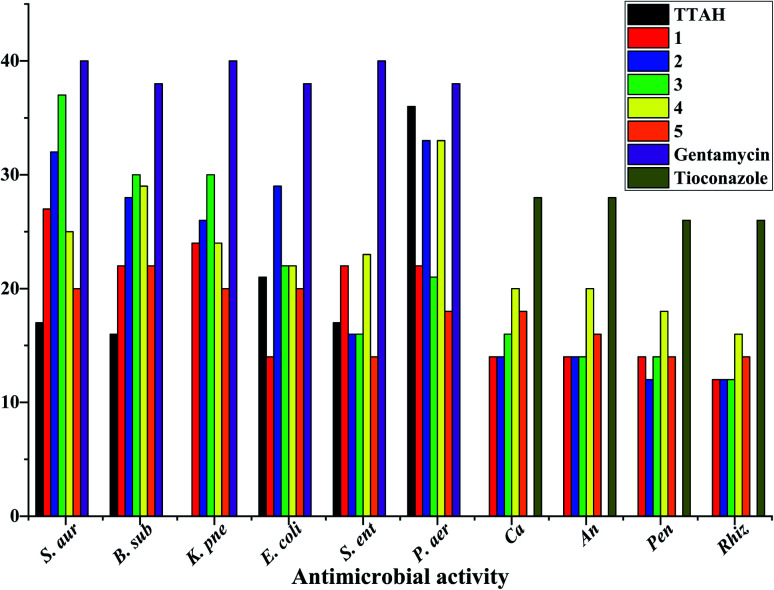
Antimicrobial activity data of mixed-ligand complexes of copper(ii) with thienoyltrifluoroacetonate and nitrogen containing ligands. N.B: S. aur = *Staphylococcus aureus*; B. sub = *Bacillus subtilis*; K. pne = *Klebsiella pneumonia*; E. coli = *Escherichia coli*; S. ent = *Salmonella enterica*; P. aer = *Pseudomonas aeruginosa*; Ca = *Candida albicans*; An = *Aspergillus niger*; Pen = *Penicillium notatum*; Rhiz = *Rhizopus*.

Laboratory strains of *Staphylococcus aureus*, *Bacillus subtilis*, *Klebsiella pneumoniae*, *Escherichia coli*, *Salmonella enterica*, *Pseudomonas aeruginosa*, *Candida albicans*, *Aspergillus niger*, *Rhizopus stolonifer* and *Penicillium notatum* were used for screening. It has been reported that Cu(ii) complexes showed improved antibacterial activities as compared with their parent ligands. It is revealed that this is by an increase of the lipophilicity of the complexes, which occurs after the complexation of the organic residue around the copper ion which favours their transfer across the lipid membrane of the bacterial cell wall.^7^ Complexes 1–5 displayed better antimicrobial activity than TTAH except against the Gram(−) P. aer. In complexes 3–5, the activity against the Gram(+) and Gram(−) bacteria appears to depend on the nature of the co-ligand {phen > bipy > tmen} except for Gram(−) S. ent. and P. aer. where the order is bipy > phen > tmen. Their antifungal activity, however, follows the order bipy > tmen > phen. In complexes 1 and 2, the observed trends against Gram(+) and Gram(−) bacteria follow the order bipy > phen except for Gram(−) S. ent. where a reversed trend is observed (Fig. 9).

## Conclusions

The copper(ii) complexes were characterized using elemental analysis, mass spectrometry, spectroscopic studies, antimicrobial activity, cytotoxicity assay studies, Hirshfeld analysis, DFT, and single-crystal X-ray diffraction measurements. The antibacterial activity of the copper complexes compared favourably with that of gentamicin used at 10 μg mL^−1^. With respect to antifungal activity, the standard drug (tioconazole) used at 70% concentration had higher activity than the copper(ii) complexes except [Cu(TTA)bipyCl] (4) in *Candida albicans* and *Aspergillus niger* which had higher activity. The cytotoxicity test revealed that [Cu(TTA)phenNO_3_] (1) has the lowest cytotoxicity amongst the complexes studied. In order to compare the experimental geometric parameters with the computed values, the dispersion corrected DFT method was employed, which gives good agreement. HOMO, LUMO, and HOMO–LUMO gaps were computed and Hirshfeld surface analysis addressed the percentage of non-covalent interactions.

## Conflicts of interest

There are no conflicts to declare.

## Supplementary Material

RA-012-D2RA02428D-s001

RA-012-D2RA02428D-s002
