# Supplementary material for: Mixed-ligand complexes of copper(ii) with thienoyltrifluoroacetonate and nitrogen containing ligands: synthesis, structures, antimicrobial activity, cytotoxicity, Hirshfeld surface analysis and DFT studies
Source: RSC Adv. 2022 Aug 17;12(36):23513–26. doi: 10.1039/d2ra02428d (PMC9382361; doi:10.1039/d2ra02428d)
Supplement: RA-012-D2RA02428D-s001 [file RA-012-D2RA02428D-s001.pdf]

Electronic Supplementary Material (ESI) for RSC Advances.
This journal is © The Royal Society of Chemistry 2022

Supporting Information

Mixed-ligand complexes of copper(II) with thienoyltrifluoroacetonate and nitrogen containing ligands: synthesis, structures, antimicrobial activity, cytotoxicity, Hirshfeld surface analysis and DFT studies

Helen Oluwatola Omoregie[a*], Oluwakemi A. Oloba-Whenu[b], Tolulope M. Fasina[b], Alexandra Friedrich[c], Martin Haehnel[c], Todd B. Marder[c]

[a]Department of Chemistry, Faculty of Science, University of Ibadan, Nigeria

[b]Department of Chemistry, Faculty of Science, University of Lagos, Nigeria

[c]Institute of Inorganic Chemistry, Julius-Maximilians-Universität Würzburg, Am Hubland, 97074 Würzburg, Germany

Electronic Supplementary Information (ESI)

Electronic Supplementary Information (ESI) Available:

Table of Contents

Antimicrobial activity and cytotoxicity data -- S2-3

Geometry optimized molecular structures of the title square planar complexes-- S4

Optimized structures at the PBE1PBE/6-311g(d,p) level of theory for the square based pyramidal complexes------------------------ S5

Selected optimized and experimental geometric parameters for the complexes--S6-8

Electrostatic potential surfaces of the copper complexes at the PBE1PBE/6-311g(d,p) level of theory---------------------------------S9

Fingerprint plots for the complexes---S10-15

Hirshfeld surfaces---S16

Summary of the various interactions (in %) in the crystal of the complexes--S17

Solid-state molecular structures of the complexes--S18-20

Single-crystal X-ray diffraction data and refinement---S21-22

Selected bond lengths (Å), distances (Å), and angles (°) of the complexes--S23

Continuous Shape measurements (CSM) of the complexes---S24

Calculated absorption UV-visible spectra from TD-DFT at the m06-2x/6-311++G(d,p) level of theory ---------------------------------S24

UV/Visible and IR Spectra---S25-50

Table S1: Antimicrobial activity and cytotoxicity data of mixed-ligand complexes of copper(II) with thienoyltrifluoroacetonate and nitrogen containing ligands

Compound	*S. aur*	*B. sub*	*K. pne*	*E. coli*	*S. ent*	*P. aer*	*C.a*	*A.n*	*Pen*	*Rhiz*	LC_{50}
TTA-H	17	16	ND	21	17	36	ND	ND	ND	ND	ND
[Cu(TTA)(phen)NO_3]	27	22	24	14	22	22	14	14	14	12	157.99
[Cu(TTA)(bipy)NO_3]	32	28	26	29	16	33	14	14	12	12	115.82
[Cu(TTA)(phen)Cl]	37	30	30	22	16	21	16	14	14	12	107.66
[Cu(TTA)(bipy)Cl]	25	29	24	22	23	33	20	20	18	16	152.48
[Cu(TTA)tmenCl]	20	22	20	20	14	18	18	16	14	14	104.76
Gentamicin(10µg/ml)	40	38	40	38	40	38	ND	ND	ND	ND	
Tioconazole(70%)	ND	ND	ND	ND	ND	ND	28	28	26	26	
Methanol	No activities										

Growth inhibition zone in millimeters (mm)

10-19-moderately active 20-29-active; 30 and above- very active

R= organism resistant to the extract ND=not determined

N.B:

S. aur = *Staphylococcus aureus*

B. sub = *Bacillius subtilis*

K. pne = *Klebsiella pneumonia*

E. coli = *Escherichia coli*

S. ent = *Salmonella enterica*

P. aer = *Pseudomonas aeruginosa*

Ca = *Candida albicans*

An = *Aspergillus niger*

Pen =*Penicillium notatum*

Rhiz =*Rhizopus*

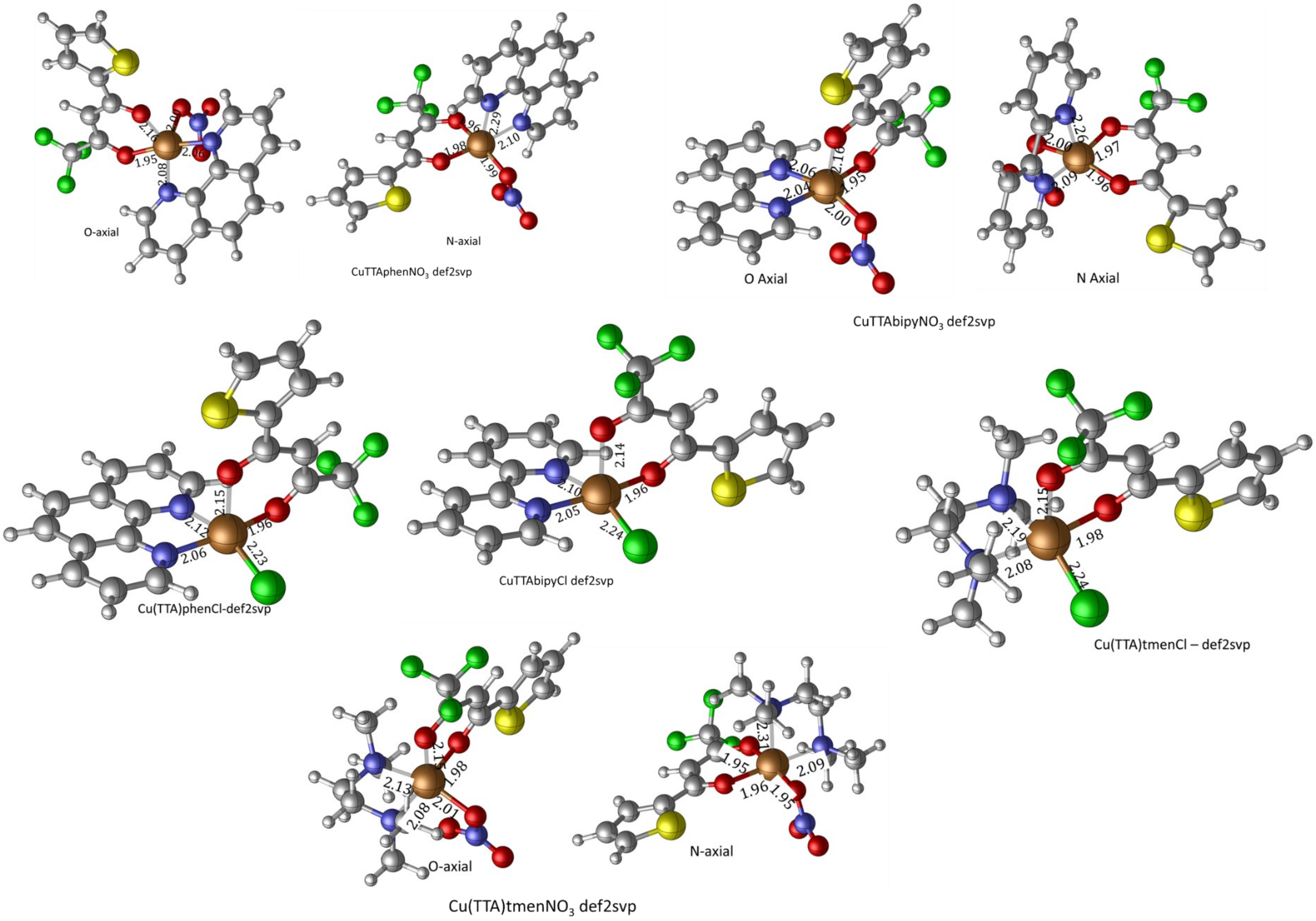

Figure S1: Geometry optimized molecular structures of the title square planar complexes starting from the experimentally determined structures at the PBE1PBE/def2svp level of theory.

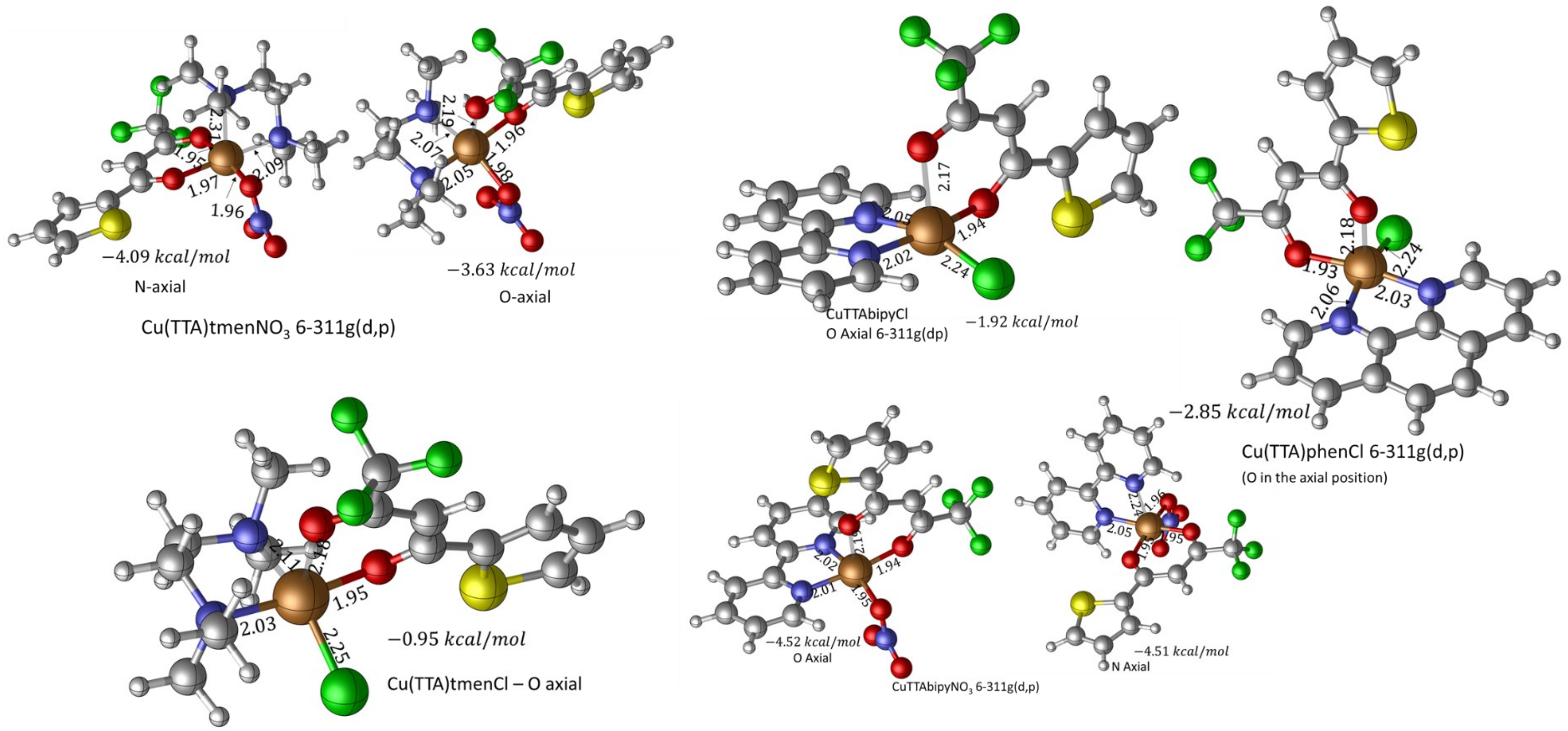

Figure S2: Geomerty optimized structures at the PBE1PBE/6-311g(d,p) level of theory for the square based pyramidal complexes in which either the oxygen atom of the TTA ligand or nitrogen atom of the corresponding amine/imine is in the axial position. The relative energy values with respect to when NO_3 or Cl is the ligand are given for each optimized structure.

Table S2: Selected optimized and experimental geometric parameters for Cu(TTA)PhenNO_3 at the PBE1PBE/6-311g(d,p) level of theorydisplayed in Figure 6, in the main article

	Cu(TTA)phenNO_3		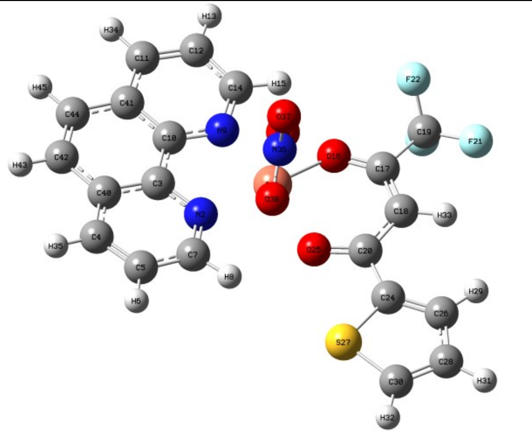
	Opt	Exp	
Cu1-O39	2.11	2.241(2)	
Cu1-O16	1.93	1.927(2)	
Cu1-O25	1.95	1.929(2)	
Cu1-N2	2.01	1.996(3)	
Cu1-N9	2.03	2.017(3)	
O39-Cu-O16	95.6	93.52(10)	
O39-Cu-O25	103.2	104.97(9)	
O39-Cu-N2	93.9	93.27(11)	
O39-Cu-N9	92	85.41(10)	
O39-Cu-O16-C17	76.7	90.8(3)	
O39-Cu-N9-C10	-89.1	-88.0(3)	
N9-N2-O25-O16	3.7	1.72(12)	

Note: O16 (opt) ≡ O1 (exp); O25 (opt) ≡ O2 (exp); O39 (opt) ≡ O3 (exp); N9 (opt) ≡ N2 (exp); N2 (opt) ≡ N1 (exp); C17 (opt) ≡C17 (exp); C10 (opt) ≡ C6 (exp)

Table S3: Selected optimized and experimental geometric parameters for Cu(TTA)BipyNO_3 at the PBE1PBE/6-311g(d,p) level of theorydisplayed in Figure 6, in the main article
.

	Cu(TTA)bipyNO_3		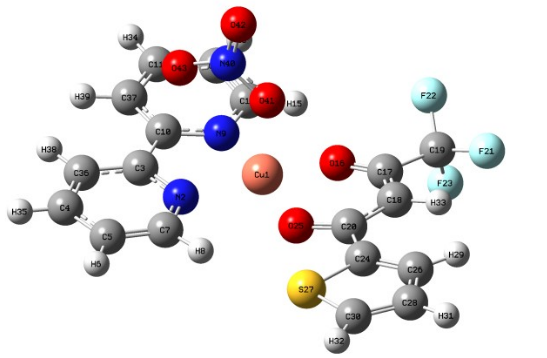
	Opt	Exp	
Cu1-O41	2.13	2.280(7)	
Cu1-O16	1.94	1.926(4)	
Cu1-O25	1.95	1.918(4)	
Cu1-N2	2	1.978(4)	
Cu1-N9	1.99	1.975(4)	
O41-Cu-O16	95.9	89.87(16)	
O41-Cu-O25	93.1	105.95(15)	
O41-Cu-N2	96.9	95.92(17)	
O41-Cu-N9	94.8	88.03(17)	
O41-Cu-O16-C17	72.2	95.9(4)	
O41-Cu-N9-C10	-85.9	-93.5(4)	
N9-N2-O25-O16	-3.5	4.98(17)	

Note: O16 (opt) ≡ O2 (exp); O25 (opt) ≡ O1 (exp); O41 (opt) ≡ O3 (exp); N9 (opt) ≡ N1 (exp); N2 (opt) ≡ N2 (exp); C17 (opt) ≡ C14 (exp); C10 (opt) ≡ C5 (exp)

Table S4: Selected optimized and experimental geometric parameters for Cu(TTA)tmenCl at the PBE1PBE/6-311g(d,p) level of theorydisplayed in Figure 6, in the main article.

	Cu(TTA)tmenCl		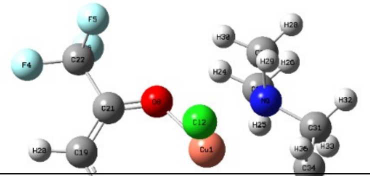
	Opt	Exp	
Cu1-Cl	2.48	2.5048(5)	
Cu1-O8	1.93	1.939(1)	
Cu1-O7	1.99	1.963(1)	

Cu1-N9	2.09	2.075(1)	
Cu1-N10	2.03	2.038(2)	
Cl-Cu-N9	101.9	102.21(4)	
Cl-Cu-N10	94.1	97.68(4)	
Cl-Cu-O7	102.2	101.41(4)	
Cl-Cu-O8	97.8	95.59(4)	
Cl-Cu-O8-C21	77.6	-106.1(1)	
Cl-Cu-N9-C31	89.1	98.42(9)	
O8-O7-N10-N9	-9	-7.54(5)	

Note: O7 (opt) ≡ O1 (exp); O8 (opt) ≡ O2 (exp); N9 (opt) ≡ N1 (exp); N10 (opt) ≡ N2 (exp); C21 (opt) ≡ C7 (exp); C31 (opt) ≡ C11 (exp)

Table S54: Selected optimized and experimental geometric parameters for Cu(TTA)tmenNO_3 at the PBE1PBE/6-311g(d,p) level of theorydisplayed in Figure 6, in the main article

	Cu(TTA)tmenNO_3	
	Opt	Exp
Cu1-O8	2.15	2.606(2)
Cu1-O7	1.93	1.929(1)
Cu1-O6	1.98	1.922(1)
Cu1-N12	2.05	2.031(2)
Cu1-N11	2.02	2.019(2)
O8-Cu-O7	88.5	84.75(6)
O8-Cu-O6	90.6	88.84(6)
O8-Cu-N12	109	98.20(6)
O8-Cu-N11	100.8	92.52(6)
O8-Cu-O7-C24	70	88.0(2)
O8-Cu-N12-C37	103.9	-103.2(1)
N11-N12-O7-O6	7.9	6.99(6)

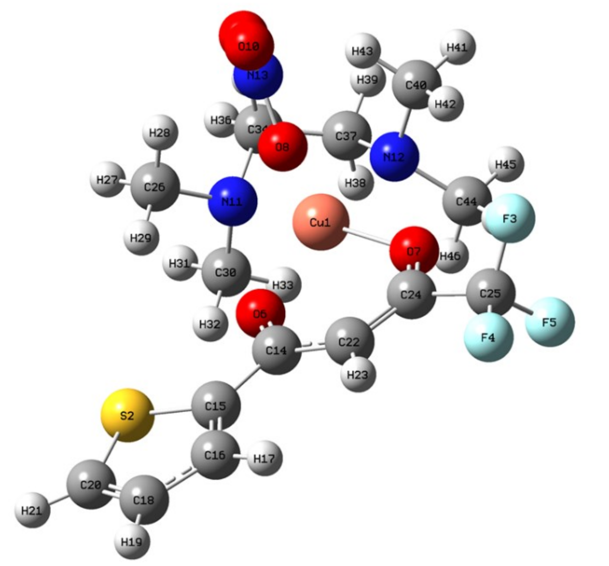

Note: O6 (opt) ≡ O2 (exp); O7 (opt) ≡ O1 (exp); O8 (opt) ≡ O5 (exp); N11 (opt) ≡ N2 (exp); N12 (opt) ≡ N1 (exp); C24 (opt) ≡ C1 (exp); C37 (opt) ≡ C11 (exp)

Table S6: Selected optimized and experimental geometric parameters for Cu$(TTA)_2$tmen at the PBE1PBE/6-311g(d,p) level of theorydisplayed in Figure 6, in the main article.

	Cu$(TTA)_2$tmen	
	Opt	Exp
Cu1 O4	2.25	2.292(1)
Cu1-O44	2.3	2.281(1)
Cu1-O13	1.98	1.960(1)
Cu1-O52	1.98	1.990(1)
Cu1-N22	2.07	2.061(2)
Cu1-N23	2.06	2.056(2)

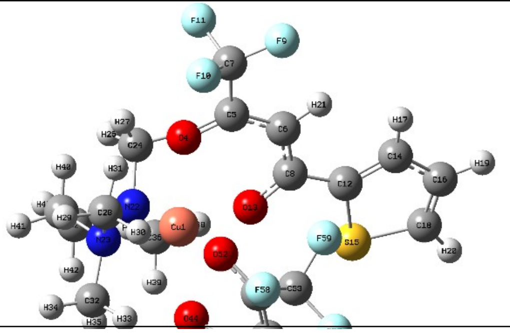

O4-Cu-O44	169.4	167.2(1)	
O4-Cu1-O13	83.6	84.74(6)	
O4-Cu1-O52	90.4	86.07(5)	
O4-Cu1-N22	94.8	99.69(6)	
O4-Cu1-N23	91.3	93.00(6)	

Note: O4 (opt) ≡ O3 (exp); O13 (opt) ≡ O4 (exp); O44 (opt) ≡ O2 (exp);O52 (opt) ≡ O1 (exp); N22 (opt) ≡ N2 (exp); N23 (opt) ≡ N1 (exp)

Table S7: Selected optimized and experimental geometric parameters for Cu(TTA)BipyCl at the PBE1PBE/6-311g(d,p) level of theory displayed in Figure 6, in the main article.

	Cu(TTA)bipyCl		
	Opt	Exp	
Cu1-Cl	2.43	2.471(1)	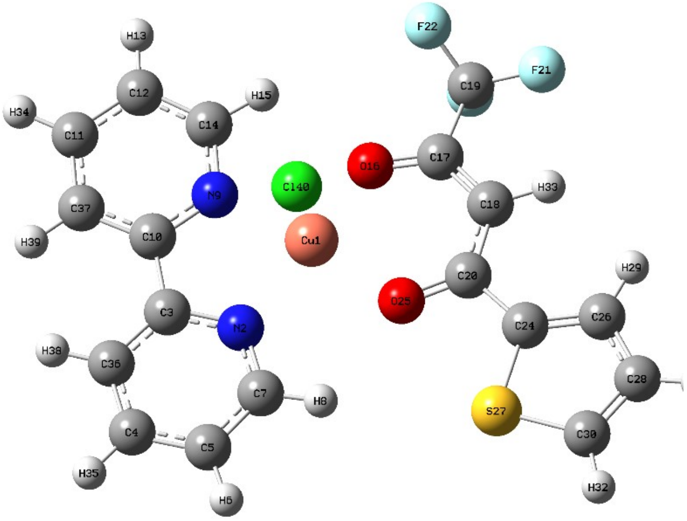
Cu1-O16	1.95	1.948(2)	
Cu1-O25	1.94	1.946(2)	
Cu1-N9	2.00	1.999(2)	
Cu1-N2	2.02	2.010(2)	
Cl-Cu-O16	105.2	97.18(5)	
Cl-Cu-O25	99.4	97.77(5)	
Cl-Cu-N9	92.7	99.83(6)	
Cl-Cu-N2	94.7	101.08(5)	
N9-N2-O25-O16	5.7	-0.61(6)	
Cl-Cu-O16-O25	99.9	98.15(5)	
Cl-Cu-N9-N2	94.3	-99.78(5)	
Cl-Cu-N2-C3	84.8	97.71(13)	
Cl-Cu-O16-C20	78.8	93.44(14)	

Note: O16 (opt) ≡ O1 (exp); O25 (opt) ≡ O2 (exp); N9 (opt) ≡ N1 (exp); N2 (opt) ≡ N2 (exp)

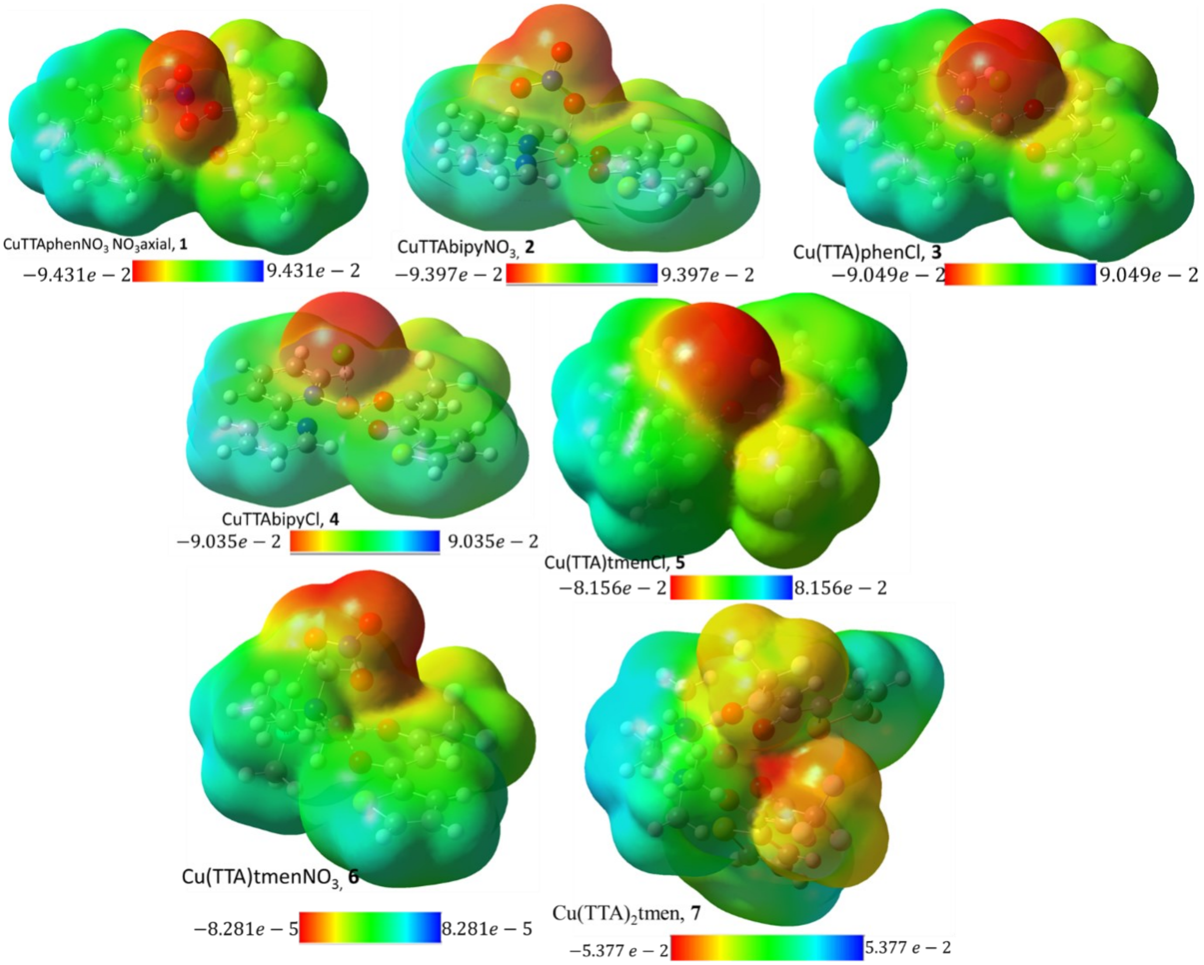

Figure S3: Electrostatic potential surfaces of the copper complexes at the PBE1PBE/6-311g(d,p) level of theory.

Hirshfeld Analysis

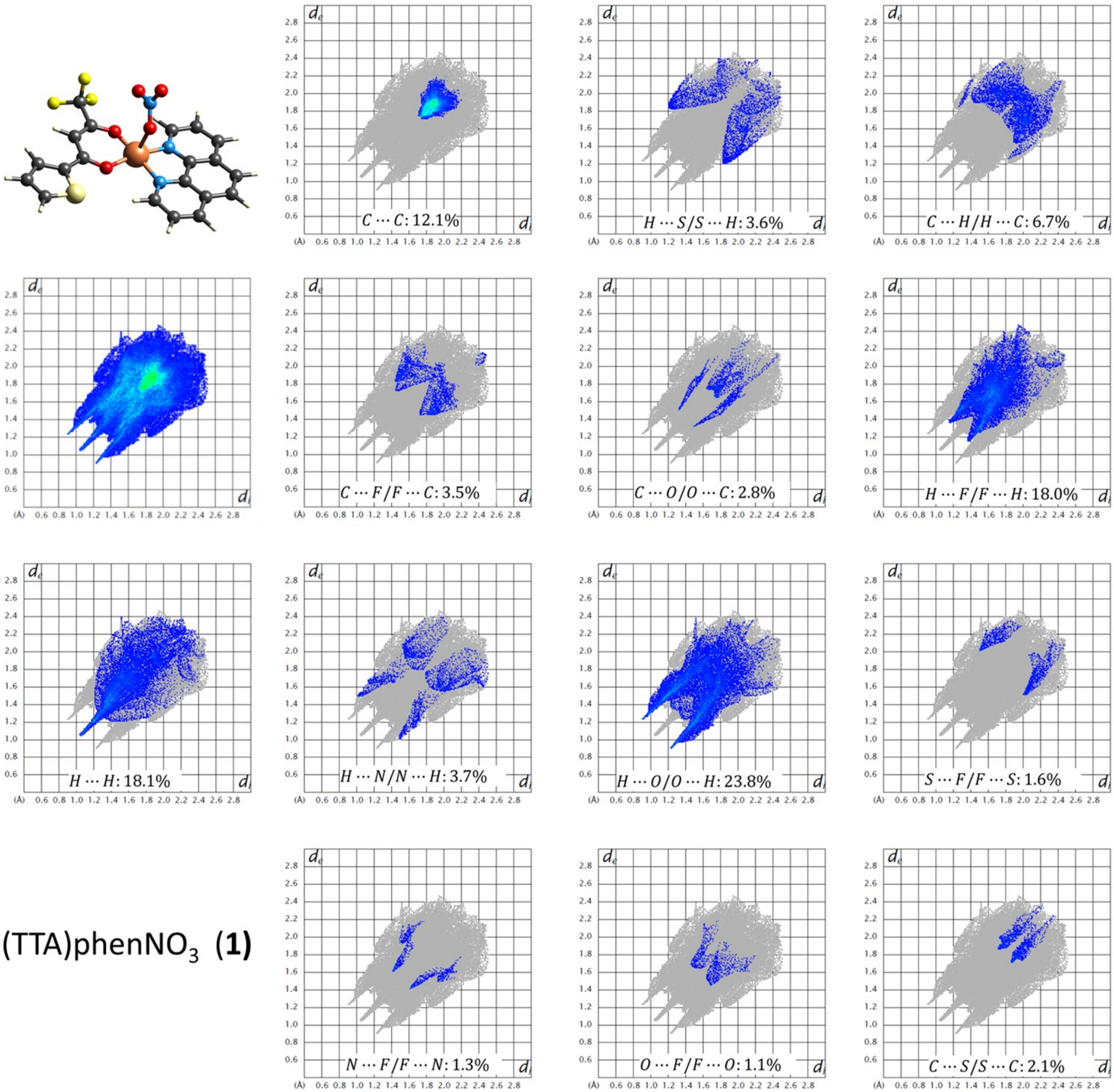

Figure S4: Fingerprint plots for CuTTA phenNO$_3$, 1. The main interactions are $H\cdots O/O\cdots H$:23.8%, $H\cdots H$: 18.1%, $H\cdots F/F\cdots H$: 18.0%, $C\cdots C$:12.1%, **other significant interactions are** $H\cdots C/C\cdots H$: 6.7%, $H\cdots N/N\cdots H$: 3.7% **(close contact),** $C\cdots O/O\cdots C$:2.84%, $C\cdots S/S\cdots C$:2.1%; $O\cdots N/N\cdots O$: 1.3%, **and** $F\cdots O/O\cdots F$:1.1%.

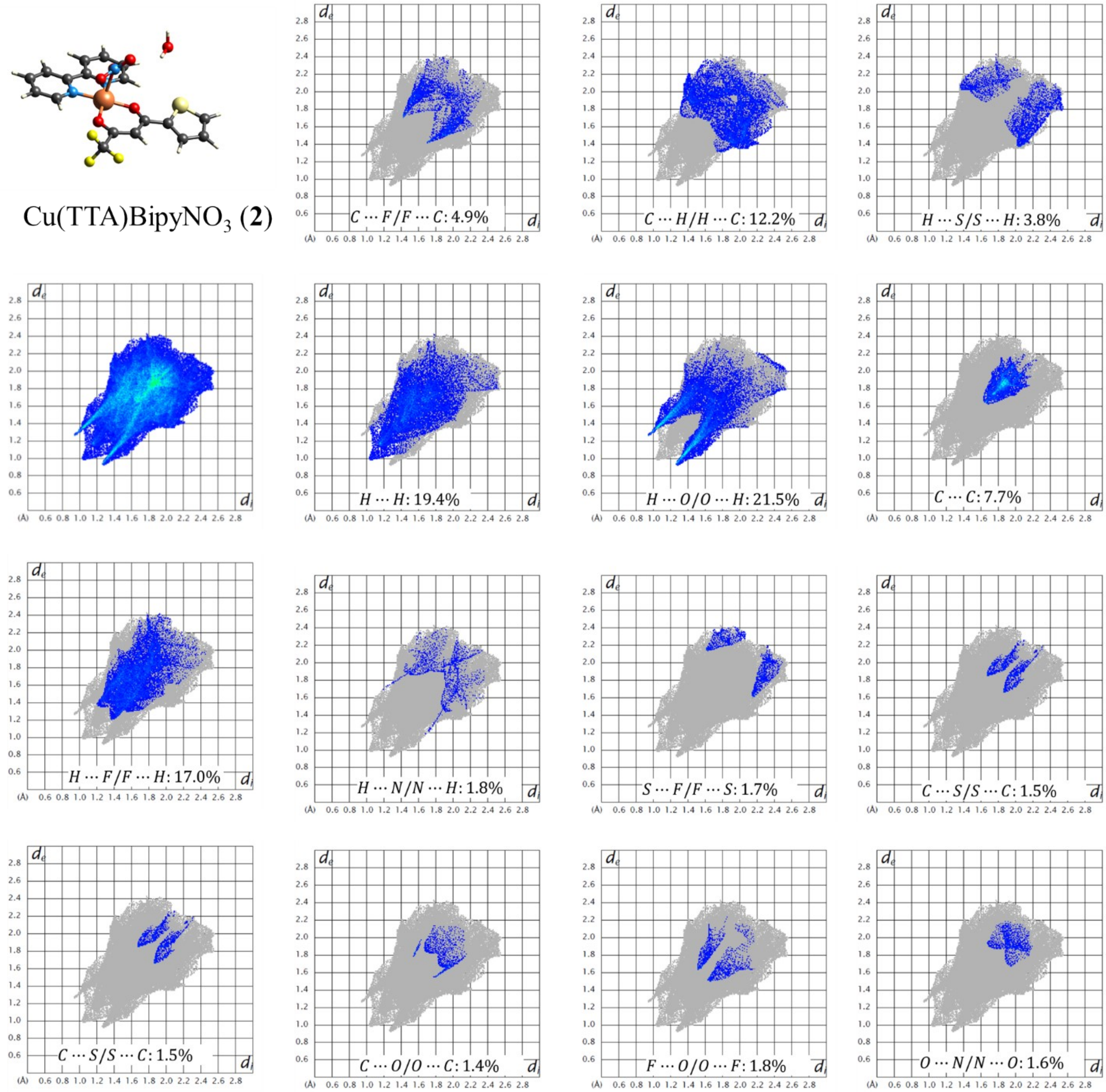

Figure S5: Fingerprint plots for Cu (TTA) bipyNO_3, 2. The main interactions are $H\cdots O/O\cdots H$: 21.5%, $H\cdots H$: 19.4%, $H\cdots F/F\cdots H$: 17.0%, $H\cdots C/C\cdots H$: 12.2%,$C\cdots C$ **contribution of** 7.7% **indicates a reasonable amout of** $\pi...\pi$ **stacking; other significant interactions are**$C\cdots F/F\cdots C$:4.9%, $H\cdots S/S\cdots H$: 3.8%, **and others are** $S\cdots F/F\cdots S$:1.7%,$H\cdots N/N\cdots H$: 1.8%, $C\cdots S/S\cdots C$:1.5%, $C\cdots O/O\cdots C$:1.4%, $F\cdots O/O\cdots F$:1.8%, **and** $O\cdots N/N\cdots O$: 1.6%.

: ;

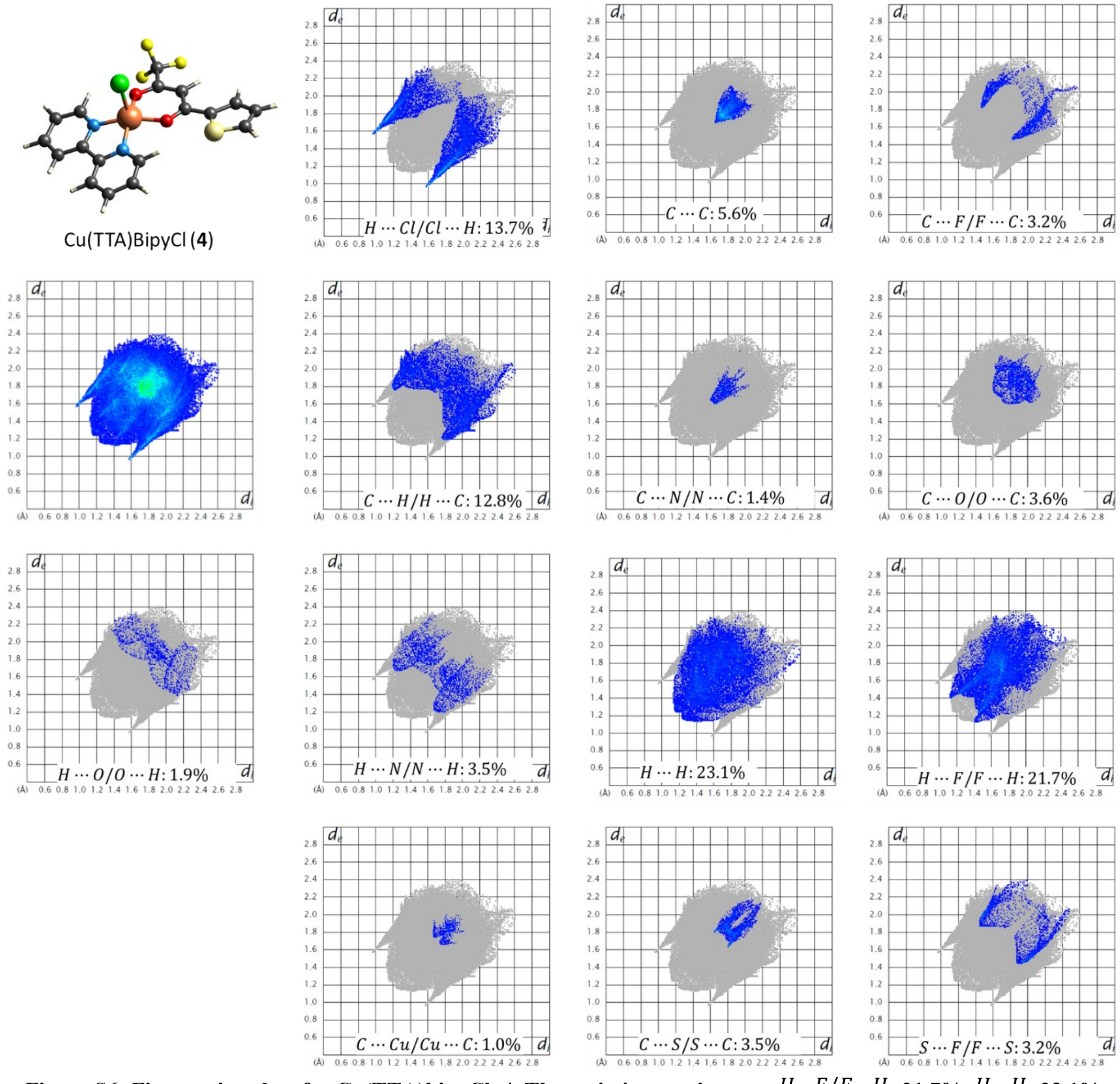

Figure S6: Fingerprint plots for Cu(TTA)bipyCl, 4. The main interactions are $H\cdots F/F\cdots H$: 21.7%, $H\cdots H$: 23.1%, $H\cdots C/C\cdots H$: 12.8%, $H\cdots Cl/Cl\cdots H$, 13.7%; $C\cdots C$, 5.6% from $\pi\ldots\pi$ stacking. Other significant interactions are $C\cdots O/O\cdots C$: 3.6%, $H\cdots N/N\cdots H$: 3.5%;$C\cdots S/S\cdots C$: 3.5%, $C\cdots F/F\cdots C$:3.2%, $S\cdots F/F\cdots S$:3.2%, **and others are** $H\cdots O/O\cdots H$:1.9%, $C\cdots N/N\cdots C$: 1.4%, **and $Cu\cdots C/C\cdots Cu$: 1.0%.**

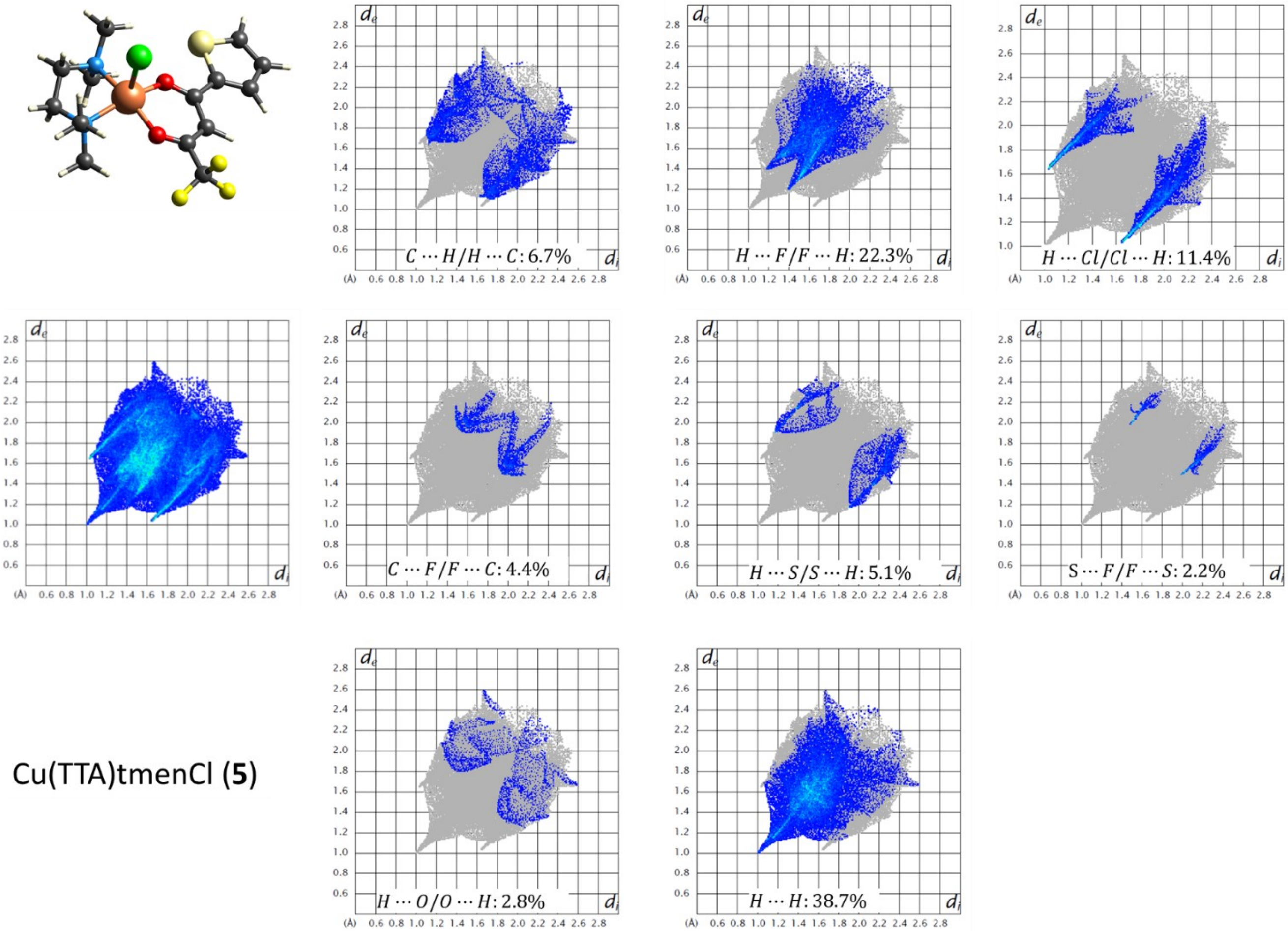

Figure S7: Fingerprint plots for Cu(TTA)tmenCl, 5. The main interactions are $H{\cdots}F/F{\cdots}H$: 22.3%, $H{\cdots}H$: 38.7% and $H{\cdots}Cl/Cl{\cdots}H$: 11.4%. Other significant interactions are $H{\cdots}C/C{\cdots}H$: 6.7%, $H{\cdots}S/S{\cdots}H$: 5.1%,**$C{\cdots}F/F{\cdots}C$:**4.4%, **$H{\cdots}O/O{\cdots}H$:**2.8%**, and $S{\cdots}F/F{\cdots}S$: 2.2%.**

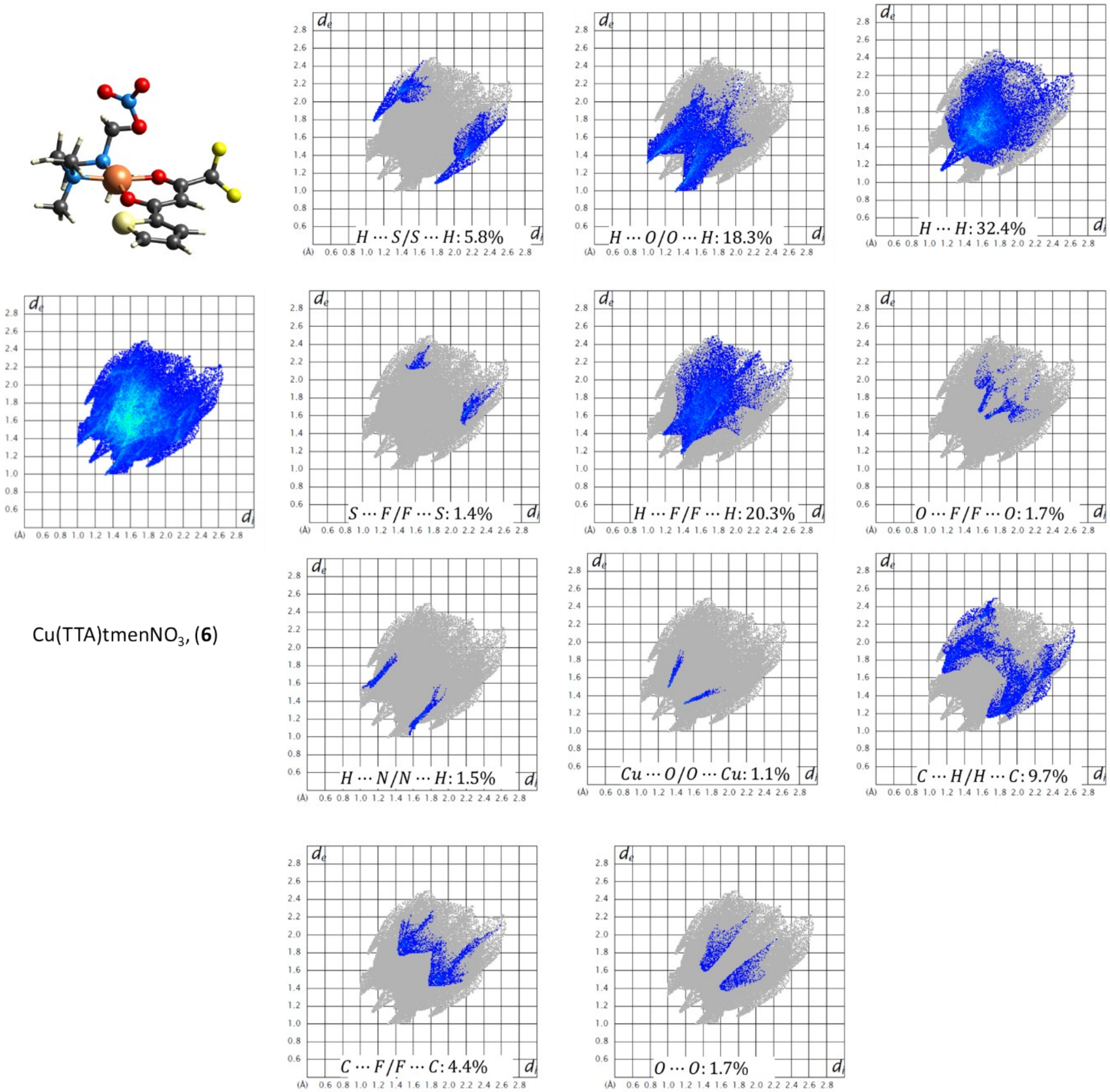

Figure S8: Fingerprint plots for CuTTAtmenNO$_3$, 6. The main interactions are $H\cdots H$: 32.4%;$H\cdots F/F\cdots H$: 20.3%, $H\cdots O/O\cdots H$: 18.3%, **and** $C\cdots H/H\cdots C$: 9.7%**; other significant interactions are** $C\cdots F/F\cdots C$:4.4%, $H\cdots S/S\cdots H$: 5.8%**;and others are** $S\cdots F/F\cdots S$:1.4%,$H\cdots N/N\cdots H$: 1.5%, $Cu\cdots O/O\cdots Cu$:1.1%,$O\cdots F/F\cdots O$:1.7%, **and** $O\cdots O$: 1.7%.

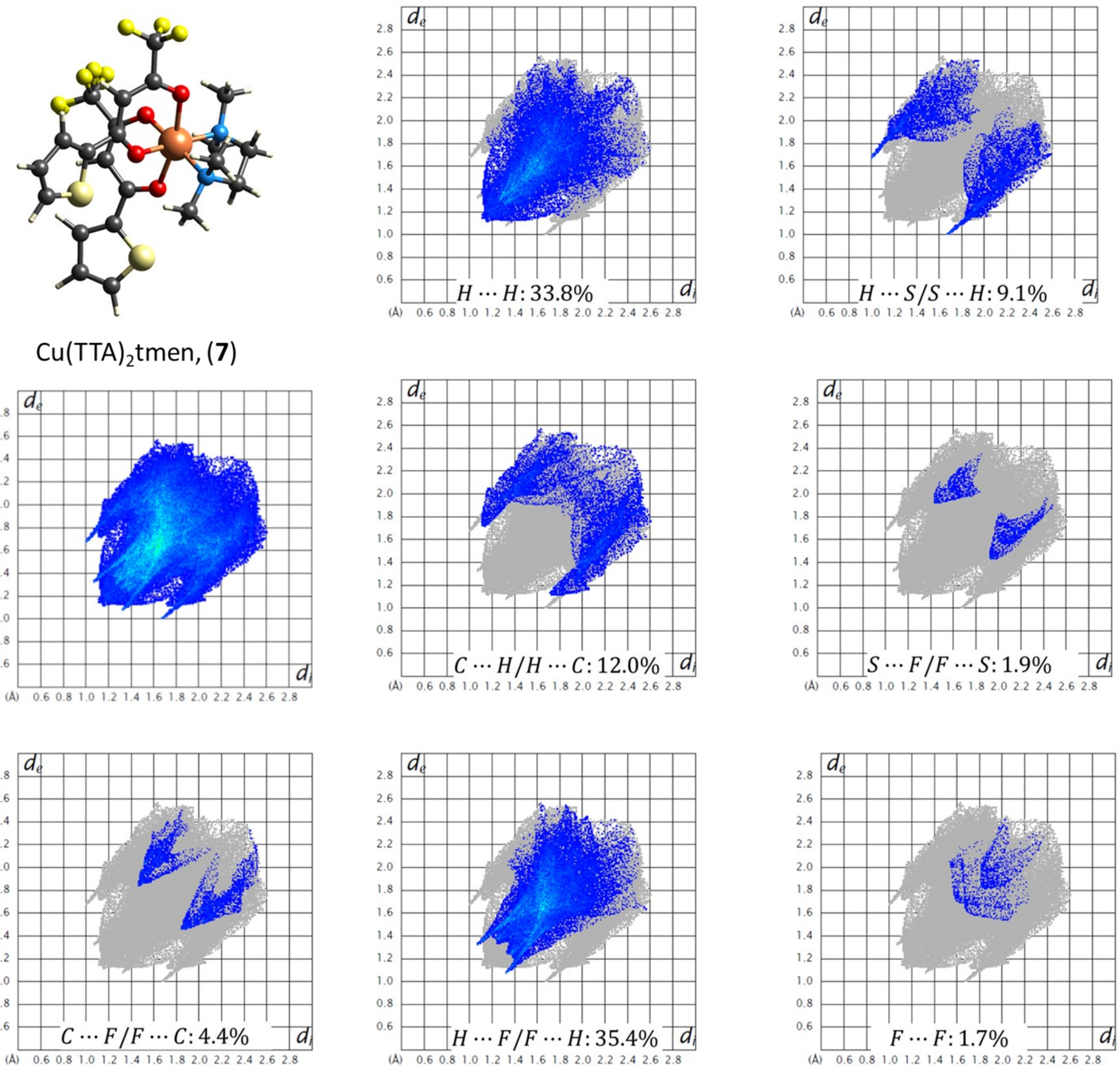

Figure S9: Fingerprint plotsfor Cu(TTA)$_2$tmen, 7. The main interactions are $H\cdots H$ **:** 33.8%, $H\cdots F/F\cdots H$:35.4%, $H\cdots C/C\cdots H$**:** 12.0%**. Others are** $H\cdots S/S\cdots H$:9.1%, $C\cdots F/F\cdots C$**:** 4.4% **and** $F\cdots F$ **:** 1.7%

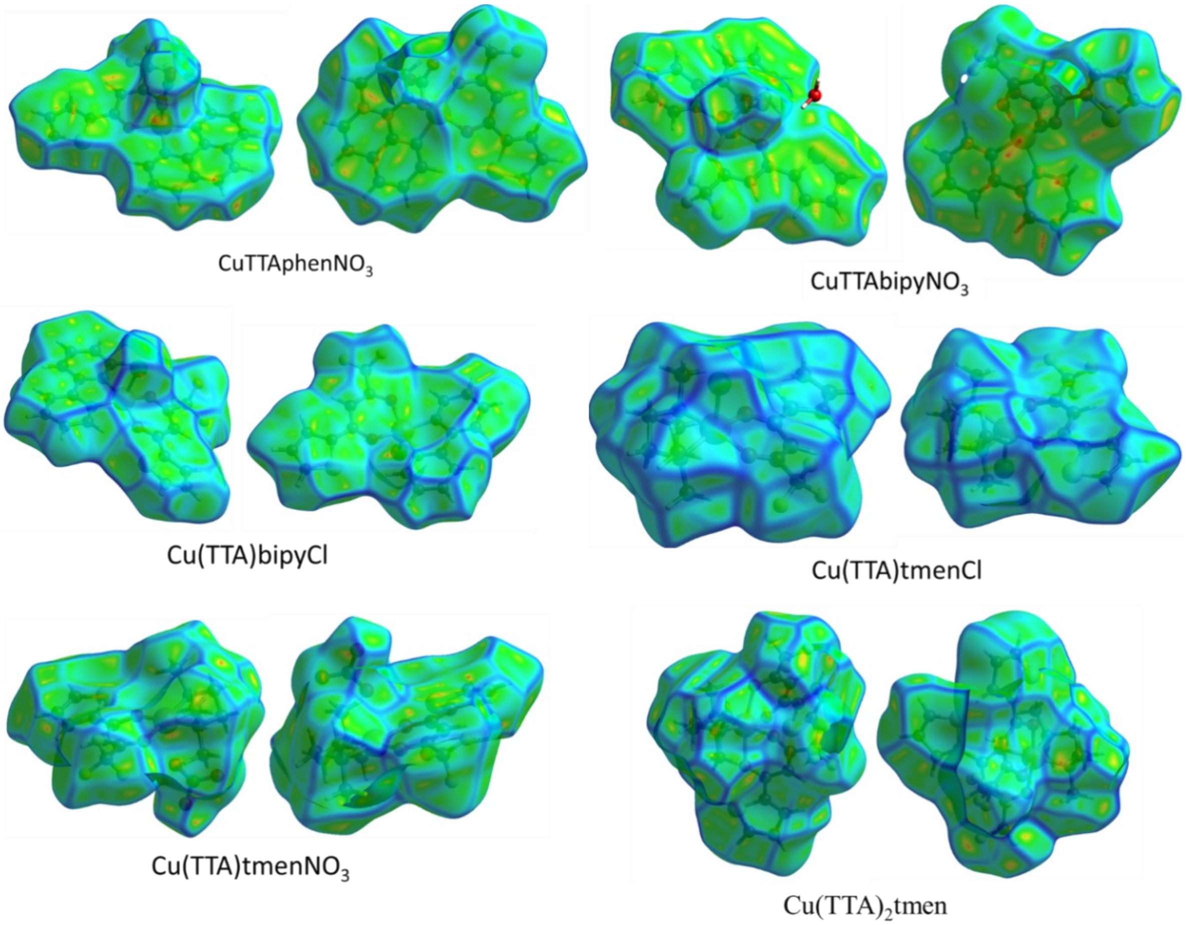

Figure S10: Hirshfeld surface mapped over curvedness property.

Table S8:Summary of the various interactions (in %) in the crystal of the complexes.

	CuTTAPhen NO_3	CuTTA bipyNO_3	CuTTAbipyCl	CuTTAtmenCl	CuTTA tmenNO_3	CuTTA$_2$tmen
**Cu···O**		0.2			1.1	
**Cu···H**	1.0		0.1			
**Cu···N**		0.1				
**Cu···C**			1.0			
**H···S**	3.6	3.8		5.1	5.8	9.1
**H···F**	18.0	17.0	21.7	22.3	20.3	35.4
**H···O**	23.8	21.5	1.9	2.8	18.3	0.9
**H···N**	3.7	1.8	3.5		1.5	
**H···C**	6.7	12.2	12.8	11.5	9.7	12.0
**S···F**	1.6	1.7	3.2	2.2	1.4	1.9
**S···S**			0.2			
**H···H**	18.1	19.4	23.1	38.7	32.4	33.8
**C···S**	2.1	1.5	3.5			0.7
**C···F**	3.5	4.9	3.2	4.4	4.4	4.4
**C···O**	2.8	1.4	3.6		0.4	
**C···C**	12.1	7.7	5.6	0.7	0.8	0.1
**C···N**	0.4	0.7	1.4			
**F···F**		0.2	0.7	0.6	0.6	1.7
**F···O**	1.1	1.8		0.1	1.7	
**F···N**	1.3	1.0				
**O···O**	0.1	0.7			1.7	
**O···N**	0.1	1.6				
**O···S**		0.2	0.5			
**N···N**		0.2				
**N···S**						
**H···Cl**		0.2	13.7	11.4		
**Cl···C**			0.4			
**Cl···F**				0.2		

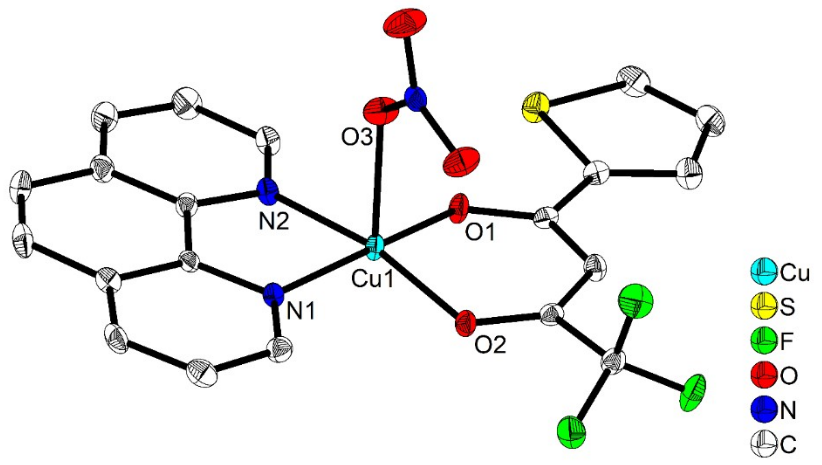

Fig. S11: Solid-state molecular structure of [Cu(TTA)phenNO_3] (1) determined by single-crystal X-ray diffraction at 100 K. All ellipsoids are drawn at the 50% probability level, and H atoms are omitted for clarity.

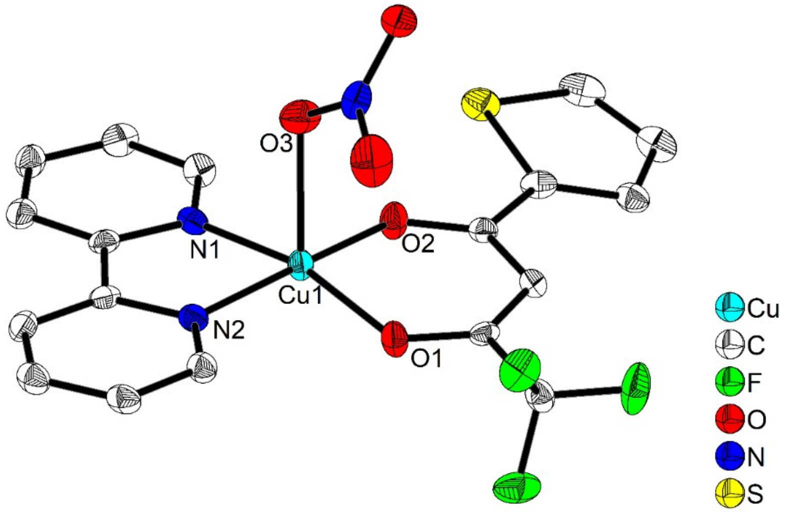

Fig. S12: Solid-state molecular structure of [Cu(TTA)bipyNO_3] (2) determined by single-crystal X-ray diffraction at 100 K. All ellipsoids are drawn at the 50% probability level, and H atoms as well as water solvent molecules are omitted for clarity.

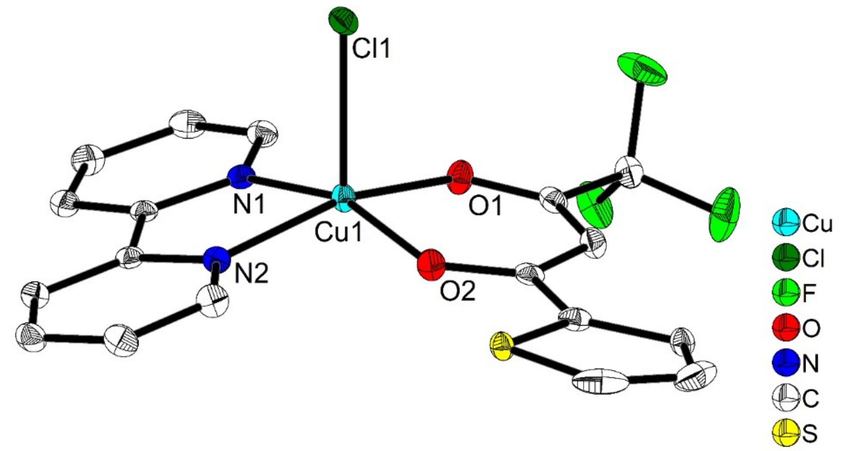

Fig. S13: Solid-state molecular structure of [Cu(TTA)bipyCl] (4) determined by single-crystal X-ray diffraction at 100 K. All ellipsoids are drawn at the 50% probability level, and H atoms are omitted for clarity. The thienyl and trifluoromethyl groups are disordered and only the major parts with 78% occupancy are shown here.

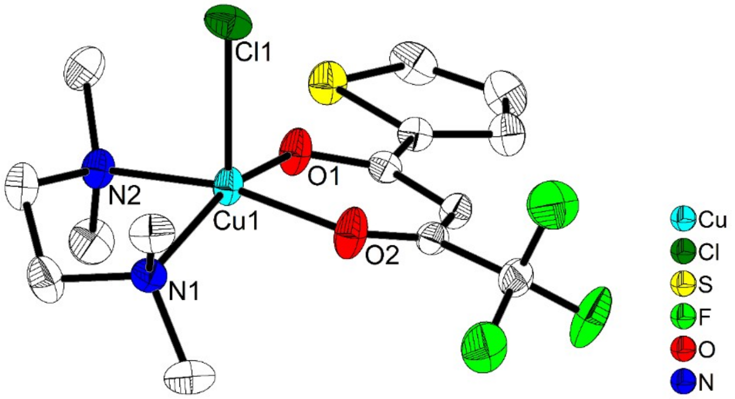

Fig. S14: Solid-state molecular structure of [Cu(TTA)tmenCl] (5) determined by single-crystal X-ray diffraction at 100 K. All ellipsoids are drawn at the 50% probability level, and H atoms are omitted for clarity.

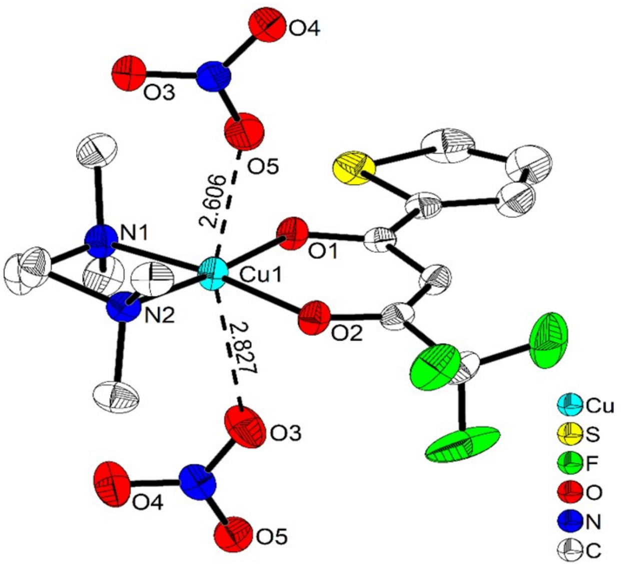

Fig. S15: Solid-state molecular structure of [Cu(TTA)tmenNO$_3$] (6) determined by single-crystal X-ray diffraction at 100 K. All ellipsoids are drawn at the 50% probability level, and H atoms are omitted for clarity.Two nitrate anions are drawn in order to show the coordination geometry around the central Cu atom. However, only one of the two anions is present in the asymmetric unit. Contact distances are given in Å.

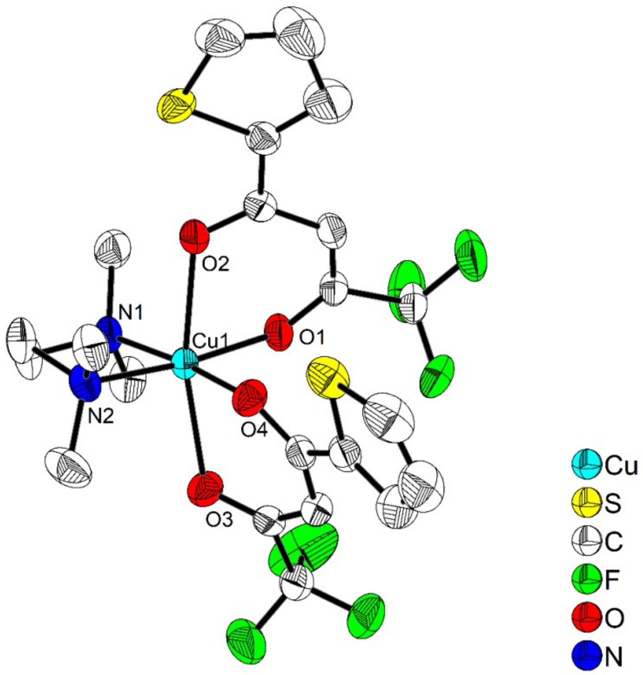

Fig. S16: Solid-state molecular structure of [Cu(TTA)$_2$tmen] (7) determined by single-crystal X-ray diffraction at 100 K. All ellipsoids are drawn at the 50% probability level, and H atoms are omitted for clarity. The thienyl and trifluoromethyl groups are disordered and only the major parts with 65% and 50% occupancy, respectively, are shown here.

Table S9: Single-crystal X-ray diffraction data and refinement details for 1,2, 4,5,6 and 7

Data	[Cu(TTA)phenNO$_3$]<br>**1**	[Cu(TTA)bipyNO$_3$]<br>**2**	[Cu(TTA)bipyCl]<br>**4**
CCDC number	2056492	2056493	2056494
Empirical formula	$C_{20}H_{12}CuF_3N_3O_5S$	$C_{18}H_{14}CuF_3N_3O_6S$	$C_{18}H_{12}CuClF_3N_2O_2S$
Formula weight / g·mol^{-1}	526.94	520.92	476.35
T / K	100(2)	100(2)	100(2)
Radiation, λ / Å	Mo-K$_\alpha$ 0.71073	Mo-K$_\alpha$ 0.71073	Mo-K$_\alpha$ 0.71073
Crystal size / mm³	0.20×0.20×0.20	0.10×0.20×0.25	0.23×0.36×0.41
Crystal colour, habit	green block	green block	yellow block
μ / mm^{-1}	1.297	1.288	1.543
Crystal system	Triclinic	Orthorhombic	Monoclinic
Space group	$P1$	$Fdd2$	$P2_1/c$
a / Å	7.6341(4)	27.42(7)	13.023(6)
b / Å	7.7048(4)	27.513(18)	8.377(4)
c / Å	9.5687(4)	10.435 (7)	17.040(7)
α/ °	95.380(2)	90	90
β / °	108.445(2)	90	106.899(13)
γ / °	110.266(2)	90	90
Volume / Å^3	487.61(4)	7872(23)	1778.7(14)
Z	1	16	4
ρ_{calc} / g·cm^{-3}	1.794	1.758	1.779
F(000)	265	4208	956
θ range / °	2.305 – 26.992	2.215 – 26.773	1.634 – 26.000
Reflections collected	10769	18494	64883
Unique reflections	3411	4201	3500
Parameters / restraints	298 / 3	296 / 5	283 / 58
GooF on F^2	1.057	1.041	1.040
R_1 [I>2σ(I)]	0.0211	0.0362	0.0249
wR_2 (all data)	0.0498	0.0855	0.0629
Max. / min. residual electron density / e·Å^{-3}	0.251 / –0.241	1.303 / –0.280	0.710 / –0.626

Table S9:Continued

Data	[Cu(TTA)tmenCl] **5**	[Cu(TTA)tmen]NO_3 **6**	[Cu(TTA)$_2$tmen] **7**
CCDC number	2056495	2056496	2056497
Empirical formula	$C_{14}H_{20}ClCuF_3N_2O_2S$	$C_{14}H_{20}CuF_3N_3O_5S$	$C_{22}H_{24}CuF_6N_2O_4S_2$
Formula weight / g·mol^{-1}	436.37	462.93	622.09
T / K	168(2)	168(2)	168(2)
Radiation, λ / Å	Mo-K$_\alpha$ 0.71073	Mo-K$_\alpha$ 0.71073	Mo-K$_\alpha$ 0.71073
Crystal size / mm³	0.35×0.45×0.55	0.50×0.50×0.50	0.40×0.40×0.60
Crystal colour, habit	green block	green block	green block
μ / mm^{-1}	1.521	1.350	1.066
Crystal system	Monoclinic	Monoclinic	Monoclinic
Space group	$P2_1/n$	$P2_1/n$	$P2_1/c$
a / Å	10.1537(14)	10.4518(14)	16.633(3)
b / Å	13.5200(19)	13.4241(18)	9.9312(18)
c / Å	13.2892(18)	13.3035(18)	16.773(3)
α/ °	90	90	90
β / °	100.416(2)	96.356(2)	108.801(2)
γ / °	90	90	90
Volume / Å^3	1794.3(4)	1855.1(4)	2622.9(8)
Z	4	4	4
ρ_{calc} / g·cm^{-3}	1.615	1.658	1.575
F(000)	892	948	1268
θ range / °	2.167 – 28.917	2.162 – 26.999	2.419 – 26.000
Reflections collected	28058	27678	43348
Unique reflections	4718	4054	5145
Parameters / restraints	221 / 0	248 / 0	403 / 43
GooF on F^2	1.061	1.051	1.116
R_1 [I>2σ(I)]	0.0250	0.0325	0.0318
wR_2 (all data)	0.0703	0.0918	0.0934
Max. / min. residual electron density / e·Å^{-3}	0.545 / –0.275	0.916 / –0.568	0.445 / –0.262

Table S10: Selected bond lengths (Å), distances (Å), and angles (°) of [Cu(TTA)phenNO_3] (**1**), [Cu(TTA)bipyNO_3] (**2**), [Cu(TTA)bipyCl] (**4**), [Cu(TTA)tmenCl] (**5**), [Cu(TTA)tmen]NO_3 (**6**), and [Cu$(TTA)_2$tmen] (**7**). For atom labels see Figures S11 – S16 in the SI.

	**1**	**2**	**4**	**5**	**6**	**7**
Cu–N1	1.996(3)	1.975(4)	1.9993(17)	2.0751(12)	2.0306(17)	2.0556(16)
Cu–N2	2.017(3)	1.978(4)	2.0096(17)	2.0376(12)	2.0189(16)	2.0610(16)
Cu–O1	1.927(2)	1.918(4)	1.9480(15)	1.9625(10)	1.9292(14)	1.9904(13)
Cu–O2	1.929(2)	1.926(4)	1.9458(15)	1.9389(10)	1.9215(14)	2.2814(13)
Cu–O3/Cl1	2.241(2)	2.280(7)	2.4712(10)	2.5048(5)	2.8265(18)	2.2916(14)
Cu–O4/O5					2.6062(16)	1.9596(14)
O1–Cu–O2	93.59(10)	92.23(15)	92.45(6)	91.52(4)	93.27(6)	83.40(5)
O1–Cu–N1	169.70(11)	165.46(17)	91.21(6)	156.10(5)	89.26(6)	92.05(6)
O2–Cu–N1	92.12(11)	91.78(17)	161.43(6)	89.78(5)	172.72(7)	94.69(6)
O1–Cu–N2	90.44(11)	92.72(16)	161.11(6)	87.09(4)	175.49(6)	174.13(6)
O2–Cu–N2	168.58(10)	171.06(17)	89.87(6)	166.67(5)	90.26(6)	91.03(6)
N1–Cu–N2	82.40(12)	81.65(18)	80.98(6)	86.20(5)	87.57(7)	86.56(6)
O1–Cu–O3/Cl1	93.52(10)	105.95(15)	97.18(5)	101.41(4)	83.41(6)	86.07(5)
O2–Cu–O3/Cl1	104.97(9)	89.87(16)	97.77(5)	95.59(4)	76.44(5)	167.17(5)
N1–Cu–O3/Cl1	93.27(11)	88.03(17)	99.83(6)	102.21(4)	97.09(6)	93.00(6)
N2–Cu–O3/Cl1	85.41(10)	95.92(17)	101.08(5)	97.68(4)	100.17(6)	99.69(6)
O1–Cu–O4/O5					84.75(6)	92.81(6)
N1–Cu–O4/O5					98.20(6)	174.49(6)
O3–Cu–O4/O5					160.49(5)	84.74(6)
N2–Cu–O4/O5					92.52(6)	88.86(7)
O2–Cu–O4/O5					88.84(6)	88.46(6)
N1–N2–O1–O2	1.74(12)	4.98(17)		-7.54(5)		
N1–N2–O2–O1			-0.61(6)		6.96(6)	
N1–N2–O4–O1						6.14(6)
N2–N1–O2–O1	1.72(12)					
Cu–plane $_{square}$	0.1574(17)	0.174(2)	0.3050(7)	0.3203(6)	0.0333(8)	0.0252(8)
∠Cu–O, plane	9.71(9)	9.62(14)	2.35(4)	1.47(3)	6.15(5), 13.36(5)	4.95(5), 7.92(5)
O3/Cl–Cu–O1–C	90.8(3)	-80.5(4)	93.44(14)	100.39(11)	-76.47(15)	
O3/Cl–Cu–O2–C	-78.1(3)	95.9(4)	-90.66(15)	-106.06(11)	79.59(15)	
O3/Cl–Cu–N2–C	-88.0(3)	84.4(3)	97.71(13)	-76.60(9)	-113.4(1)	
O3/Cl–Cu–N1–C	78.9(3)	-93.5(4)	-99.81(13)	98.42(9)	89.00(13)	
Cl1–Cu–O1–O2			98.15(5)	96.01(4)		
Cl1–Cu–N1–N2			-99.78(5)	97.05(4)		

Table S11: Continuous Shape measurements (CSM) of the complexes

**Five-coordinate complexes**						
	Lable	PP-5	vOC-5	TBPY-5	SPY-5	JTBPY-5
	Shape	Pentagon	Vacant octahedron	Trigonal bipyramid	Square pyramid	Johnson trigonal bipyramid
	Symmetry	D_{5h}	C_{4v}	D_{3h}	C_{4v}	D_{3h}
**1**		29.642	0.989	5.861	**0.868**	8.460
**2**		28.679	1.369	5.055	**0.923**	7.856
**4**		32.101	2.415	5.698	**0.598**	9.313
**5**		32.901	2.515	4.154	**0.637**	8.037
**Four-coordinate complex**						
	Lable	SP-4	T-4	SS-4	vTBPY	
	Shape	Square	Tetrahedron	Seesaw	Axially vacant trigonal bipyramid	
	Symmetry	D_{4h}	T_d	C_{2v}	C_{3v}	
**6**		**0.219**	29.348	16.071	30.862	
**Six-coordinate complex**						
	Lable	HP-6	PPY-6	OC-6	TPR-6	JPPY-5
	Shape	Hexagon	Pentagonal pyramid	Octahedron	Trigonal prism	Johnson pentagonal pyramid
	Symmetry	D_{6h}	C_{5v}	O_h	D_{3h}	C_{5v}
**7**		33.271	25.565	**0.981**	12.686	29.090

Table S12: Calculated absorption UV-visible spectra from TD-DFT at the m06-2x/6-311++G(d,p) level of theory

	**[Cu(TTA)phenNO3] 1**	**[Cu(TTA)bipyNO3] 2**	**[Cu(TTA)phenCl] 3**	**[Cu(TTA) bipyCl] 4**	**[Cu(TTA)tmenCl] 5**	**[Cu(TTA)tmenNO3] 6**	**[Cu(T[illegible] tmen[illegible] 7**
**nm**	524.55	481.17	513.34	524.42	397.14	401.93	411.2[illegible]
**cm^{-1}**	19063.96	20782.68	19480.27	19068.69	25180.04	24879.95	2431[illegible]

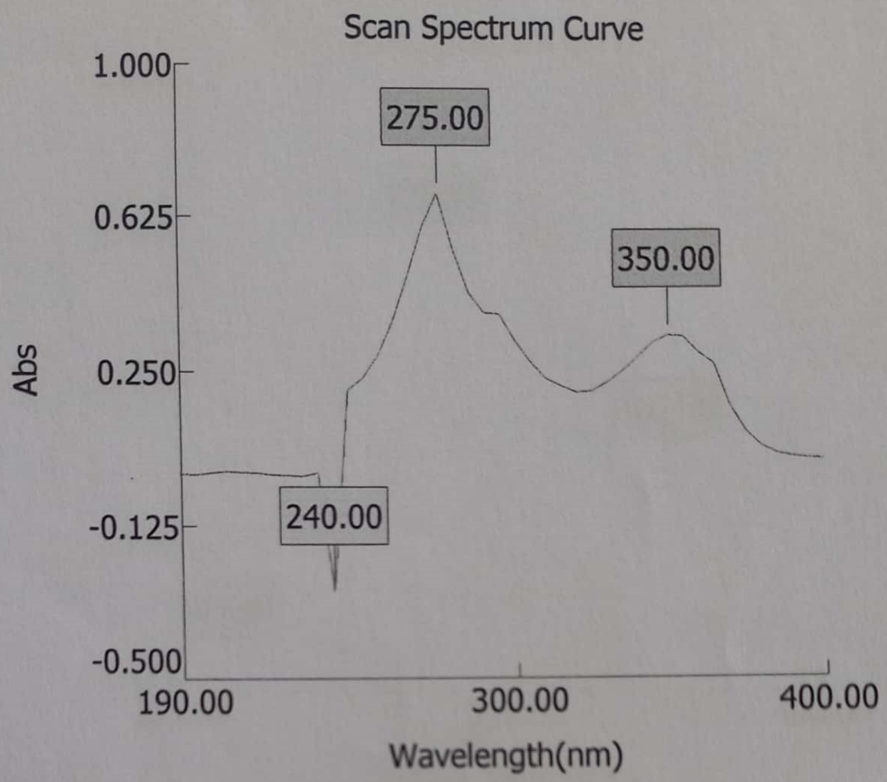

1	Peak	350.00	0.336
2	Peak	275.00	0.684
1	Valley	240.00	-0.287

Cu (TFA) Phen NO_3 in Chloroform

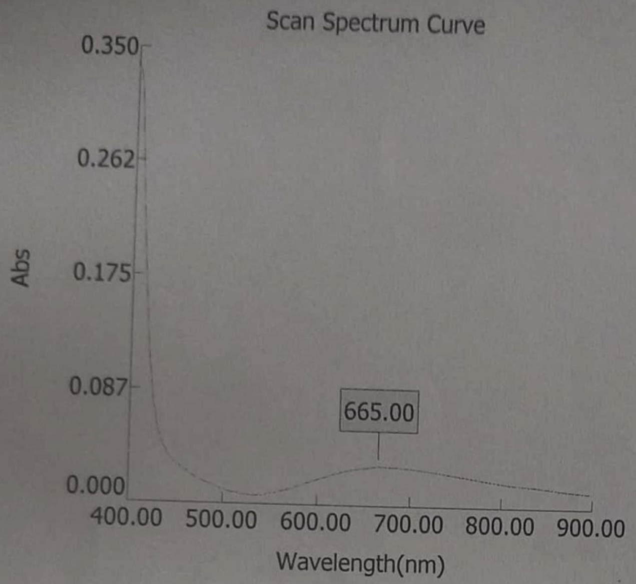

1	Peak	665.00	0.031

- **Instrument Performance**
  Model : SPECTRO UV/VIS
  Spectral Bandwidth : 2.00 nm
- **Scan Spectrum Performance**
  Scan Range : 400.00 to 900.00 nm
  Measure Mode : Abs
  Interval : 5.00 nm
  Speed : Fast
  Data File : CuTPNcv2.spd
  Create Date/Time : Sunday, January 01, 2006
  Data Type : Original
  Method File:
- **Analyse Note**
  Analyser : Administrator
  Sample Name :
  Comment :
- **No. P/V Wavelength(nm) Abs Co**

Cu(TTA)PhenNO_3 in chloroform

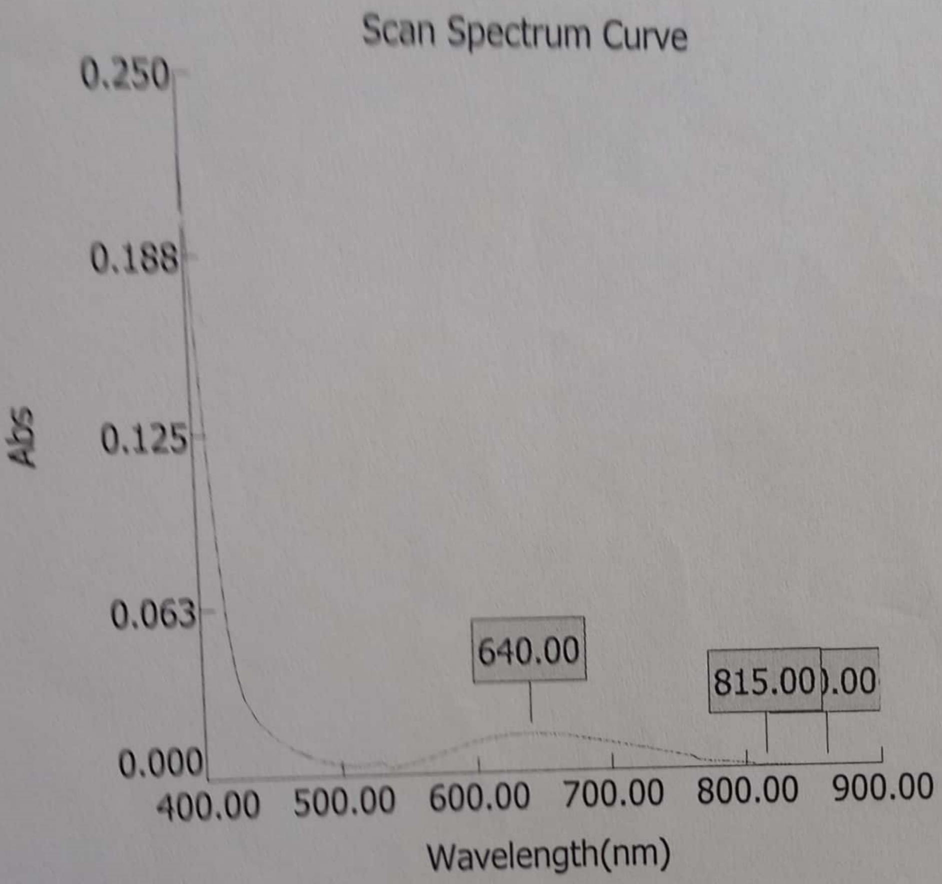

1	Peak	860.00	0.000
2	Peak	815.00	0.001
3	Peak	640.00	0.014

Cu(TTA)Phen NO_3 in

Methanol

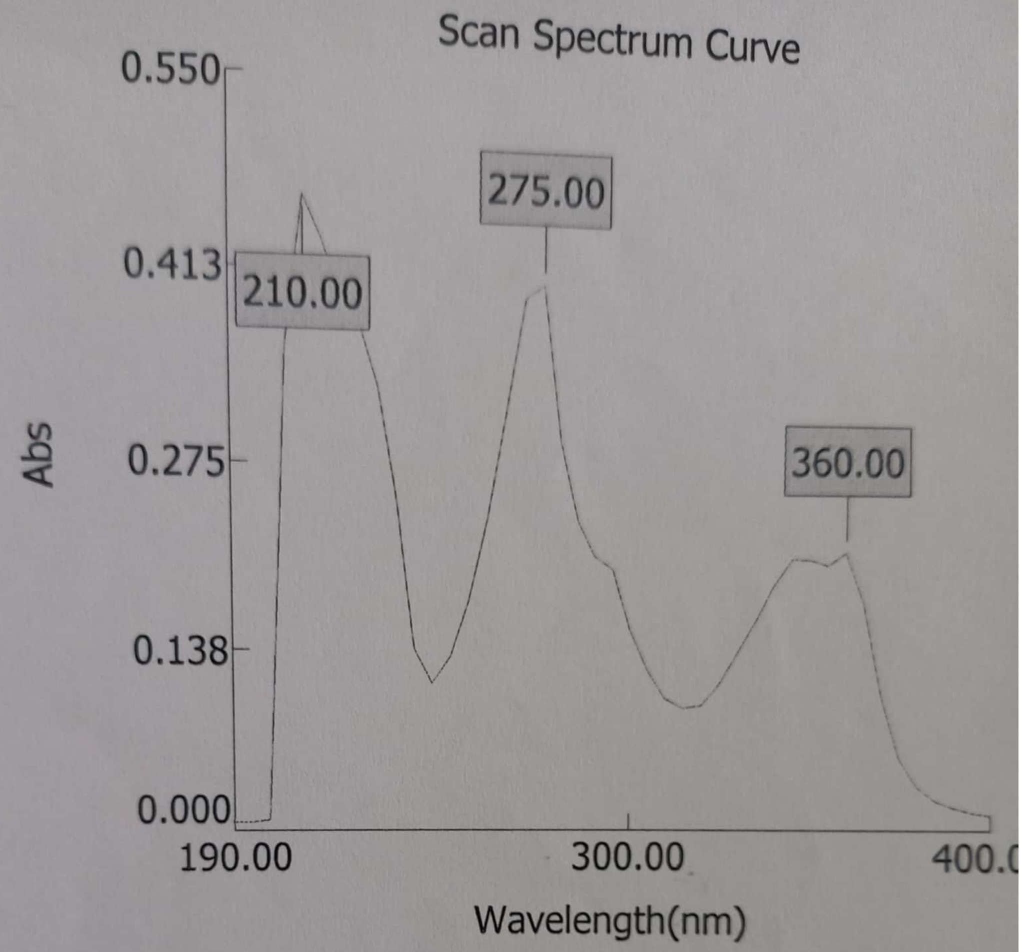

1	Peak	360.00	0.212
2	Peak	275.00	0.405
3	Peak	210.00	0.464

[Cu(TTA)Phen NO3

In UV

Scan Spectrum Curve

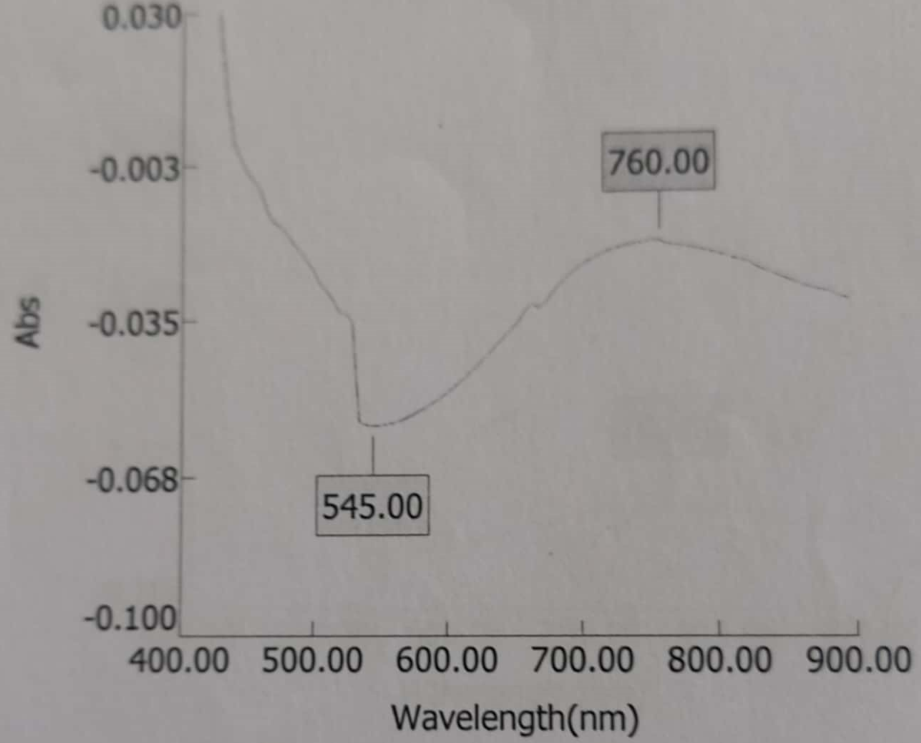

1	Peak	760.00	-0.019
1	Valley	545.00	-0.057

[Cu(iTA)Phen Cl] in

Chloroform

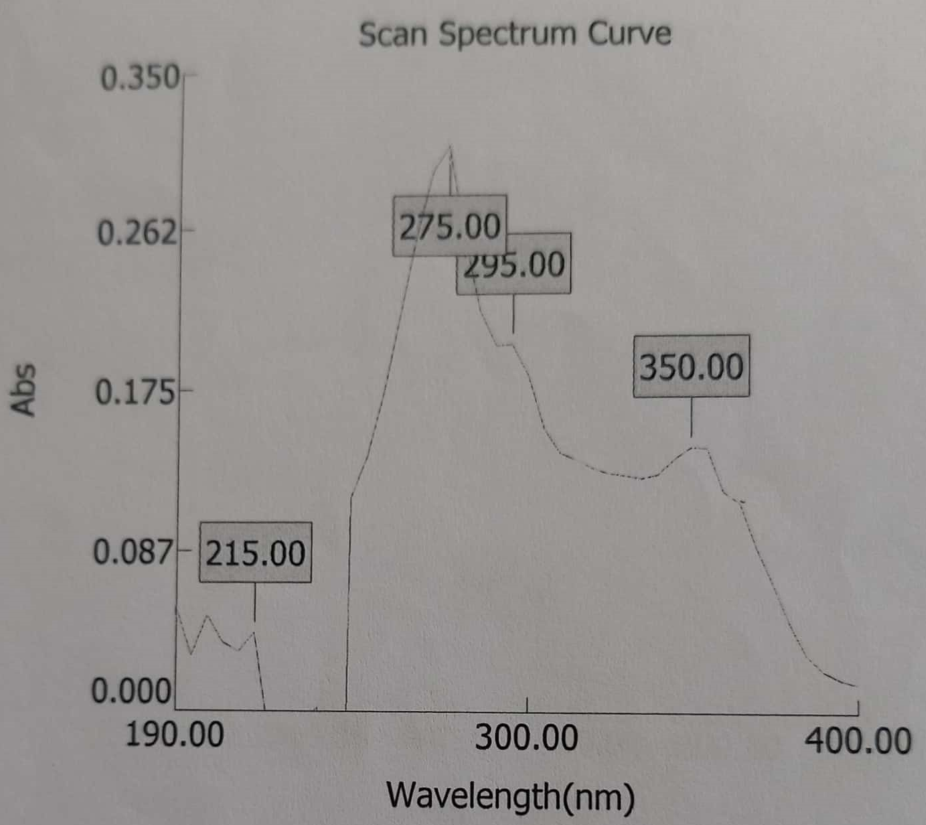

1	Peak	350.00	0.143
2	Peak	295.00	0.199
3	Peak	275.00	0.308
4	Peak	215.00	0.043

[Cu(TTA)Phen Cl]

in chloroform

Scan Spectrum Curve

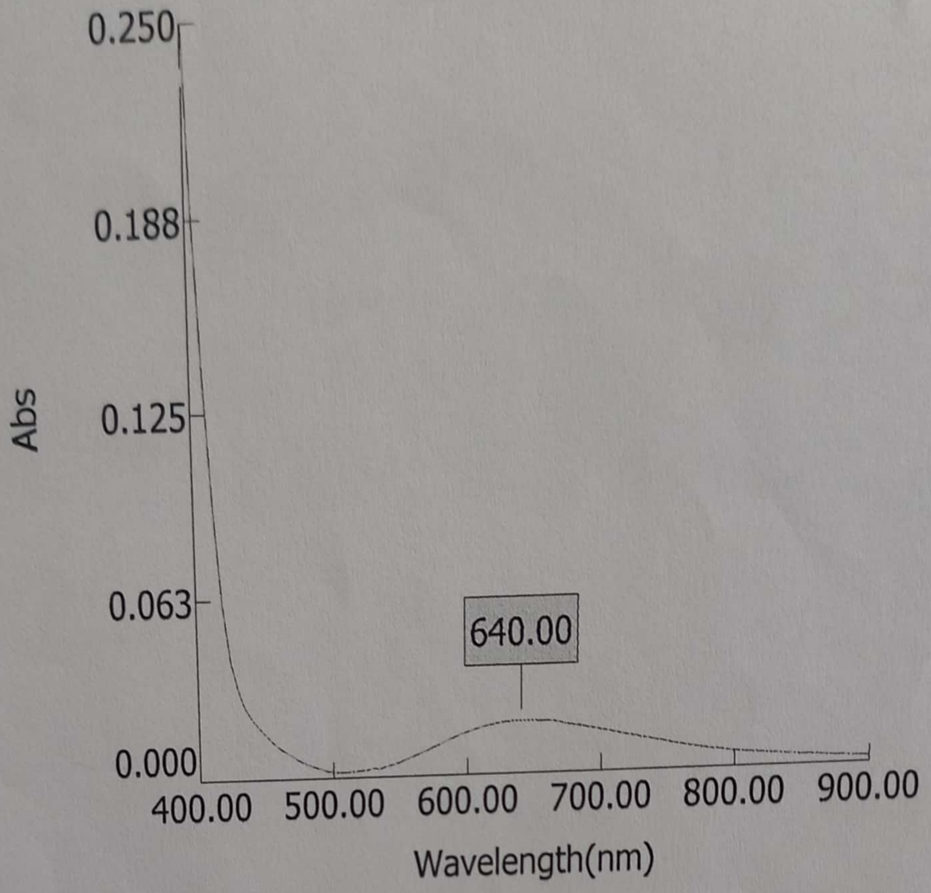

1	Peak	640.00	0.018

Cu(TTA) Phen Cl in

Methanol

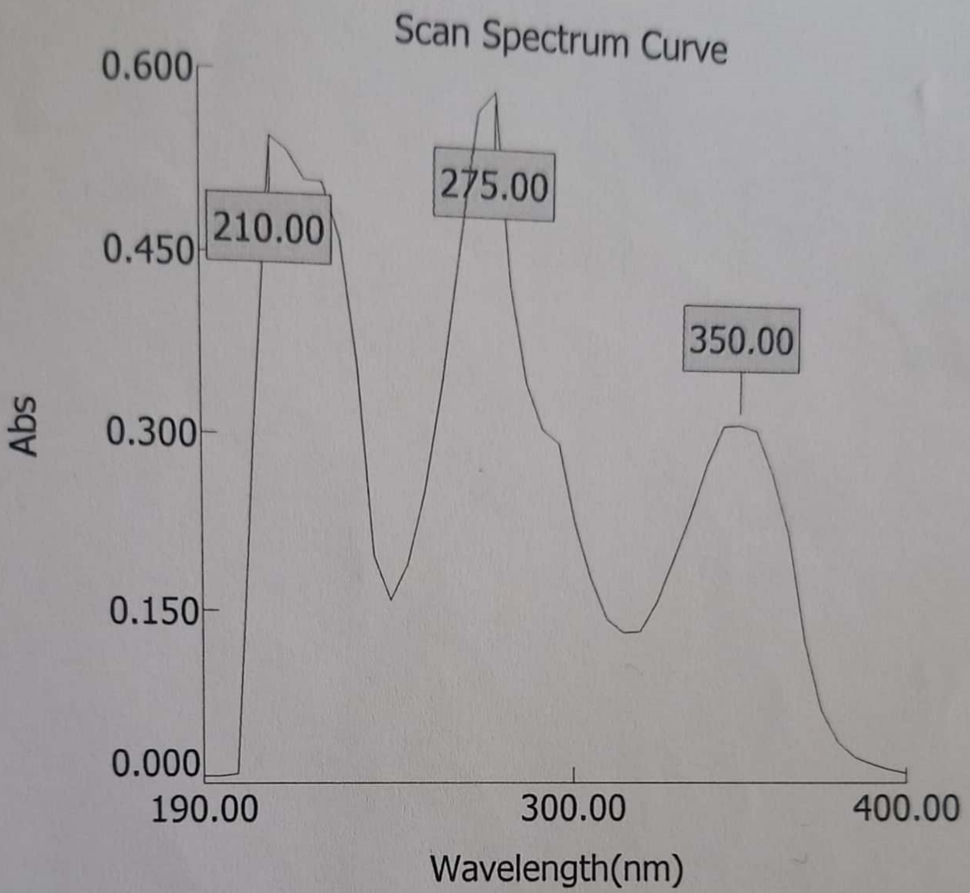

1	Peak	350.00	0.316
2	Peak	275.00	0.589
3	Peak	210.00	0.546

Cu(TTA) Phen Cl in methanol.

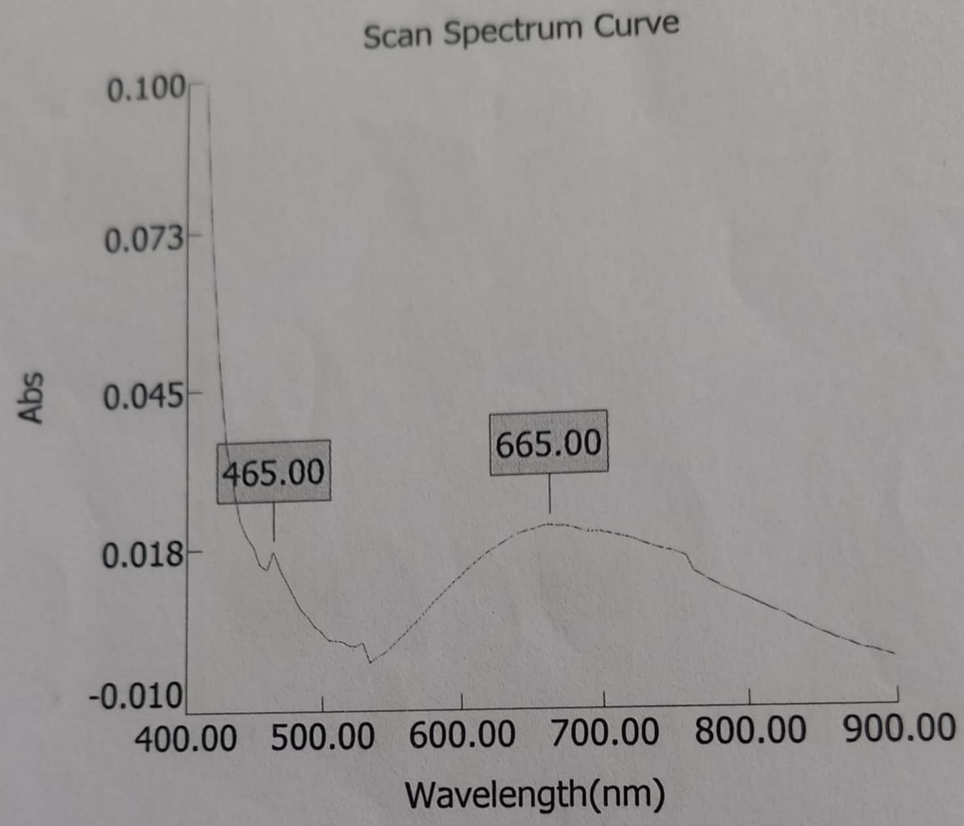

1	Peak	665.00	0.020
2	Peak	465.00	0.017

[Cu(TTA)BipyNO$_3$] in Chloroform

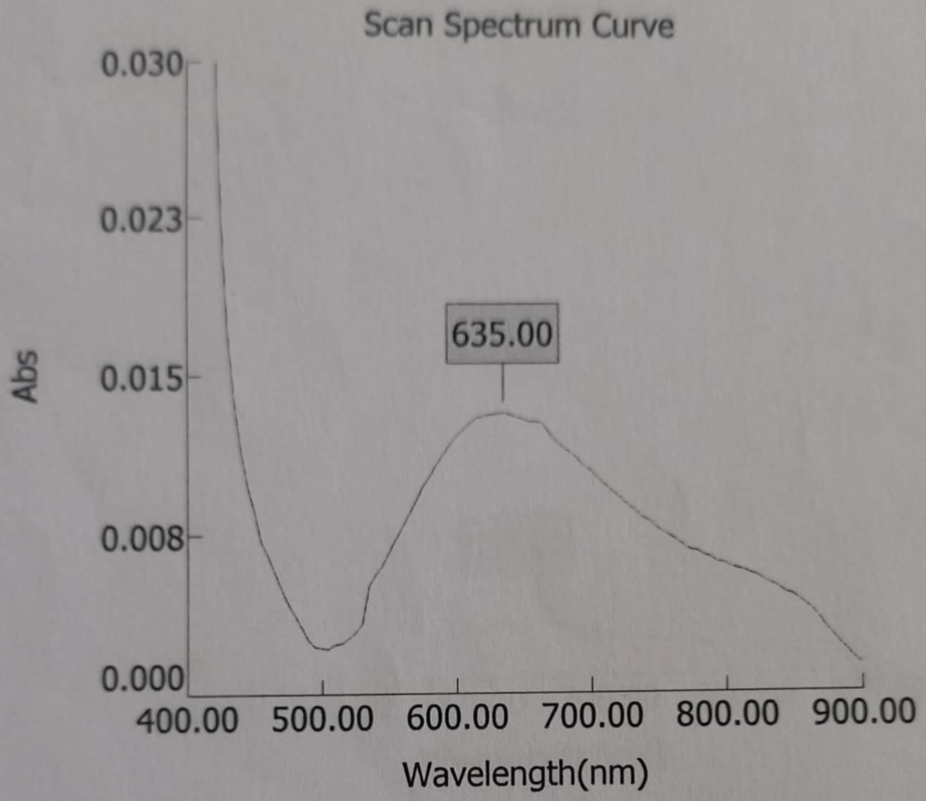

1	Peak	635.00	0.013

Cu(TTA)Bpy NO_3 in Methanol.

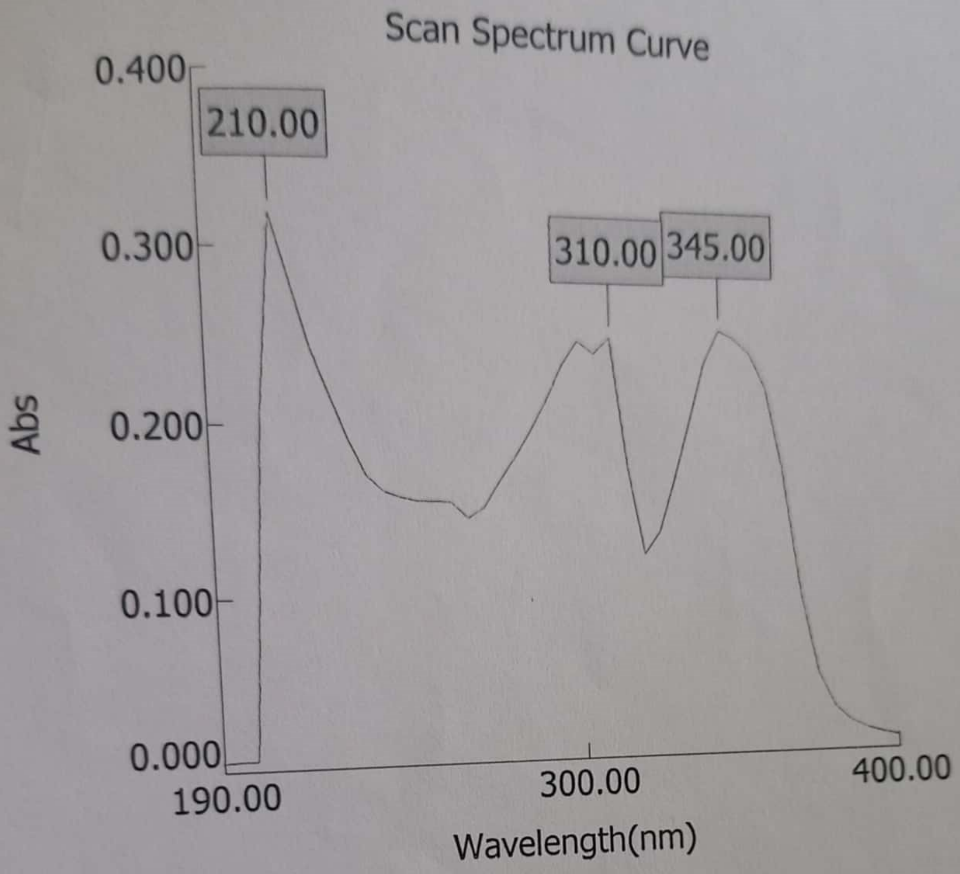

1	Peak	345.00	0.258
2	Peak	310.00	0.253
3	Peak	210.00	0.319

Cu(TTA)Bpy NO_3 in
Methanol

- In
  Mc
  Sp
- Se
  Sc
  M
  Ir
  S
  [illegible]

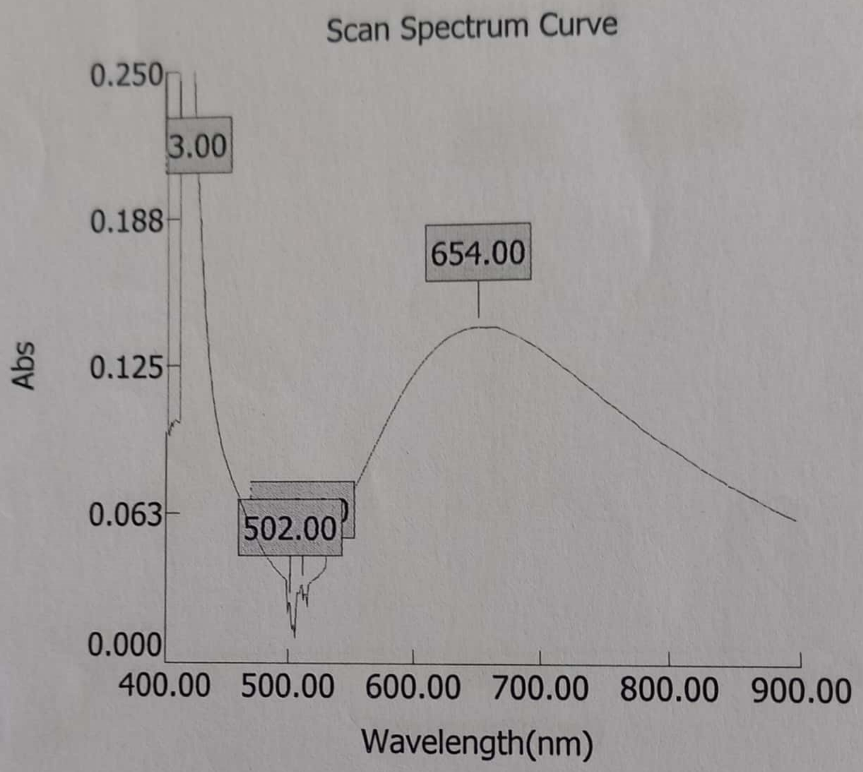

1	Peak	654.00	0.140
2	Peak	512.00	0.032
3	Peak	502.00	0.025
4	Peak	413.00	0.430

$[Cu(TTA)\,TMEN]NO_3$
in chloroform.

- In
Mc
Sp
- Sc
Sc
M
Ir
S
D
C
D
M
- A
A
S
C
- I

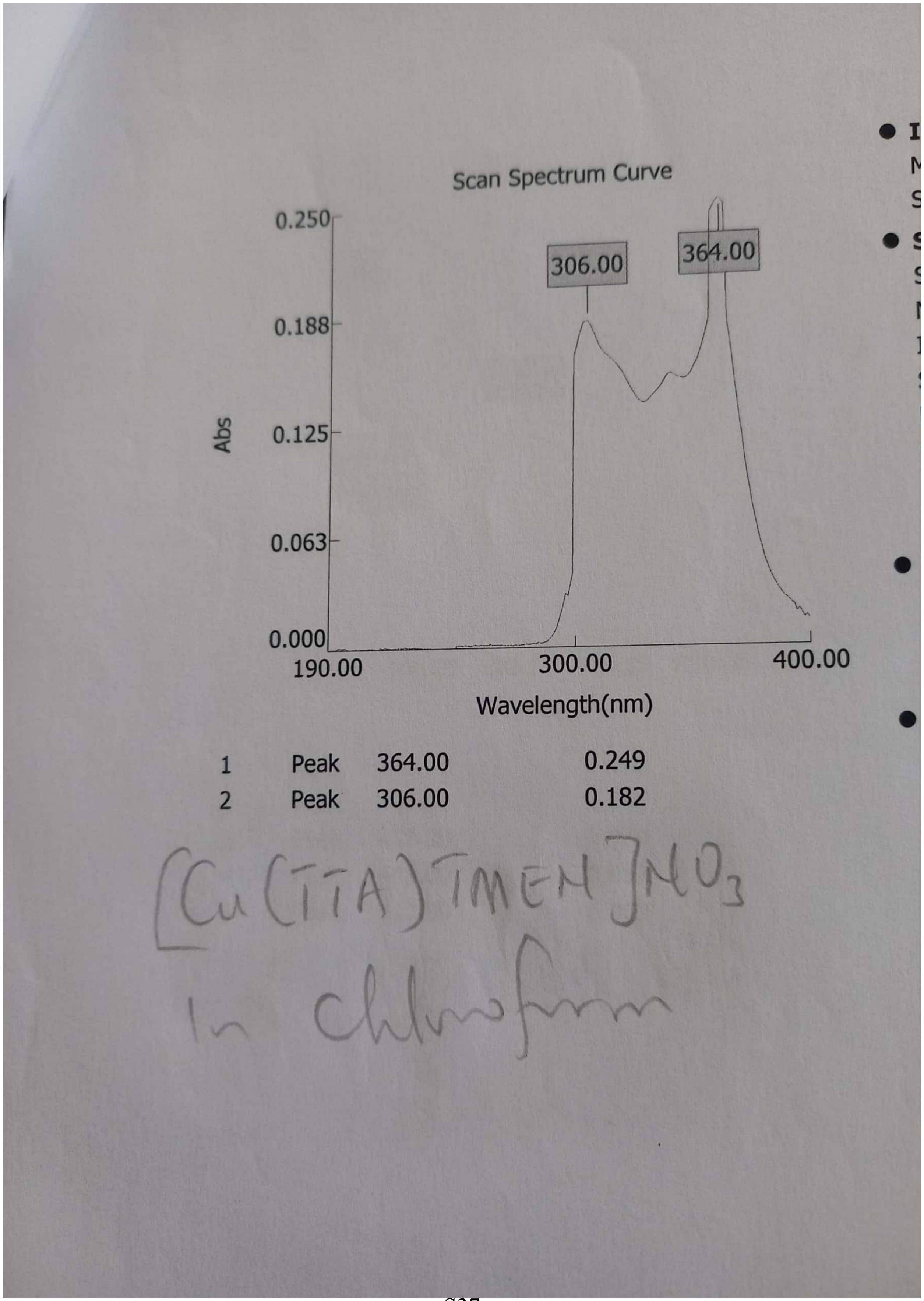
Scan Spectrum Curve
306.00
364.00
0.250
0.188
0.125
0.063
0.000
Abs
190.00
300.00
400.00
Wavelength(nm)
1 Peak 364.00 0.249
2 Peak 306.00 0.182
[Cu(TTA)TMEN]NO3
in chloroform

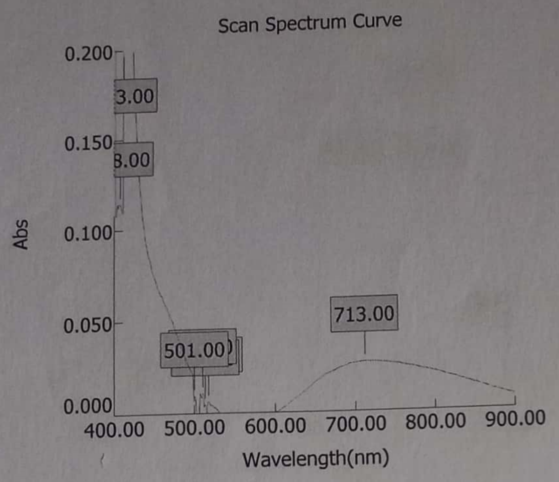

- **Instrument Performance**
  Model : SPECTRO UV/VIS
  Spectral Bandwidth : 2.00 nm
- **Scan Spectrum Performance**
  Scan Range : 400.00 to 900.00 nm
  Measure Mode : Abs
  Interval : 1.00 nm
  Speed : Medium
  Data File : CuTTATMEOacv.spd
  Create Date/Time : Sunday, January 01, 2006 1:50:31 AM
  Data Type : Original
  Method File:
- **Analyse Note**
  Analyser : Administrator
  Sample Name :
  Comment :

No.	P/V	Wavelength(nm)	Abs	Comment
1	Peak	713.00	0.027	
2	Peak	518.00	0.006	
3	Peak	514.00	0.005	
4	Peak	511.00	0.011	
5	Peak	501.00	0.009	
6	Peak	413.00	0.463	
7	Peak	408.00	0.114	

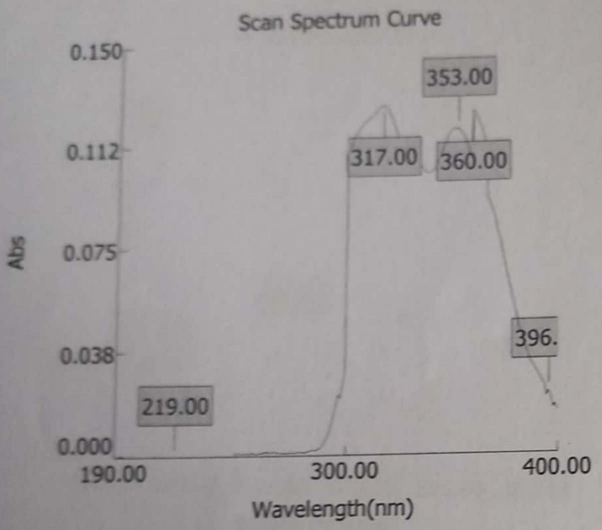

1	Peak	396.00	0.022
2	Peak	360.00	0.125
3	Peak	353.00	0.118
4	Peak	317.00	0.127
5	Peak	219.00	0.000

- **Instrument Performance**
  Model : SPECTRO UV/VIS
  Spectral Bandwidth : 2.00 nm
- **Scan Spectrum Performance**
  Scan Range : 190.00 to 400.00 nm
  Measure Mode : Abs
  Interval : 1.00 nm
  Speed : Medium
  Data File : CuTTATMEOAccuv.spd
  Create Date/Time : Sunday, January 01, 2006 2:55:26
  Data Type : Original
  Method File:
- **Analyse Note**
  Analyser : Administrator
  Sample Name :
  Comment :
- **No. P/V Wavelength(nm) Abs Comme**

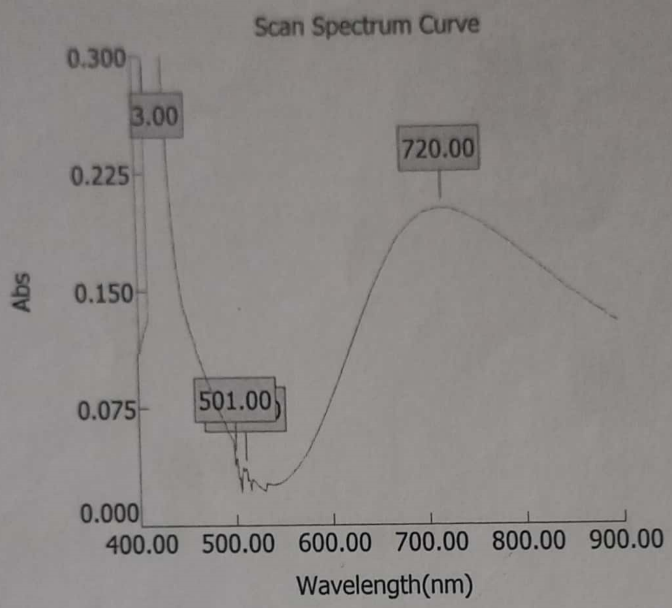

1	Peak	720.00	0.202
2	Peak	511.00	0.036
3	Peak	501.00	0.042
4	Peak	413.00	0.686

- **Instrument Performance**
  Model : SPECTRO UV/VIS
  Spectral Bandwidth : 2.00 nm
- **Scan Spectrum Performan**
  Scan Range : 400.00 to 900.0
  Measure Mode : Abs
  Interval : 1.00 nm
  Speed : Medium
  Data File : CuTTATMEClcv.sp
  Create Date/Time : Sunday,
  Data Type : Original
  Method File:
- **Analyse Note**
  Analyser : Administrator
  Sample Name :
  Comment :
- **No.** **P/V** **Wavelengt**

[Cu(TTA)Tmen Cl] in chloroform.

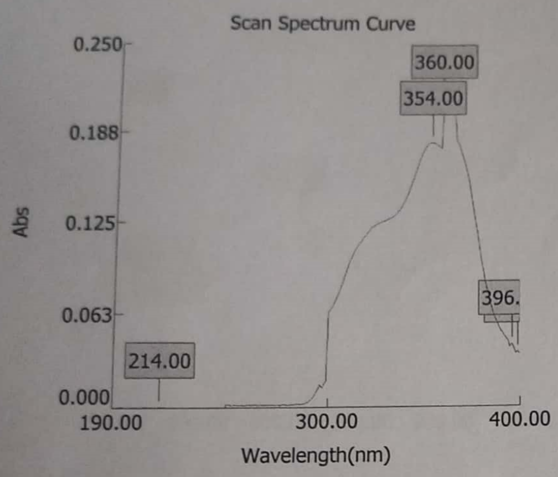

- **Instrument Performance**
  Model : SPECTRO UV/VIS
  Spectral Bandwidth : 2.00 nm
- **Scan Spectrum Performance**
  Scan Range : 190.00 to 400.00 nm
  Measure Mode : Abs
  Interval : 1.00 nm
  Speed : Medium
  Data File : CuTTATMEClcuv.spd
  Create Date/Time : Sunday, January 01, 2006 2:59:09 AM
  Data Type : Original
  Method File:
- **Analyse Note**
  Analyser : Administrator
  Sample Name :
  Comment :

No.	P/V	Wavelength(nm)	Abs	Comment
1	Peak	399.00	0.037	
2	Peak	396.00	0.042	
3	Peak	360.00	0.207	
4	Peak	354.00	0.181	
5	Peak	214.00	0.000	

[Cu(TTA) imen Cl]
in chloroform

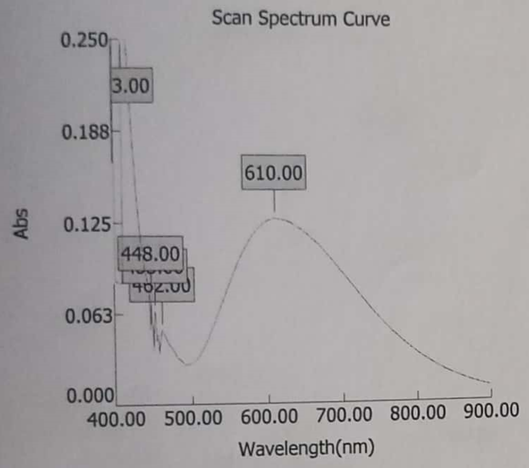

- **Instrument Performance**
  Model : SPECTRO UV/VIS
  Spectral Bandwidth : 2.00 nm
- **Scan Spectrum Performance**
  Scan Range : 400.00 to 900.00 nm
  Measure Mode : Abs
  Interval : 1.00 nm
  Speed : Medium
  Data File : CuTTATMEClmv.spd
  Create Date/Time : Sunday, January 01, 2006 1:12:56 AM
  Data Type : Original
  Method File:
- **Analyse Note**
  Analyser : Administrator
  Sample Name :
  Comment :

No.	P/V	Wavelength(nm)	Abs	Comment
1	Peak	610.00	0.129	
2	Peak	462.00	0.052	
3	Peak	453.00	0.064	
4	Peak	448.00	0.073	
5	Peak	413.00	0.328	

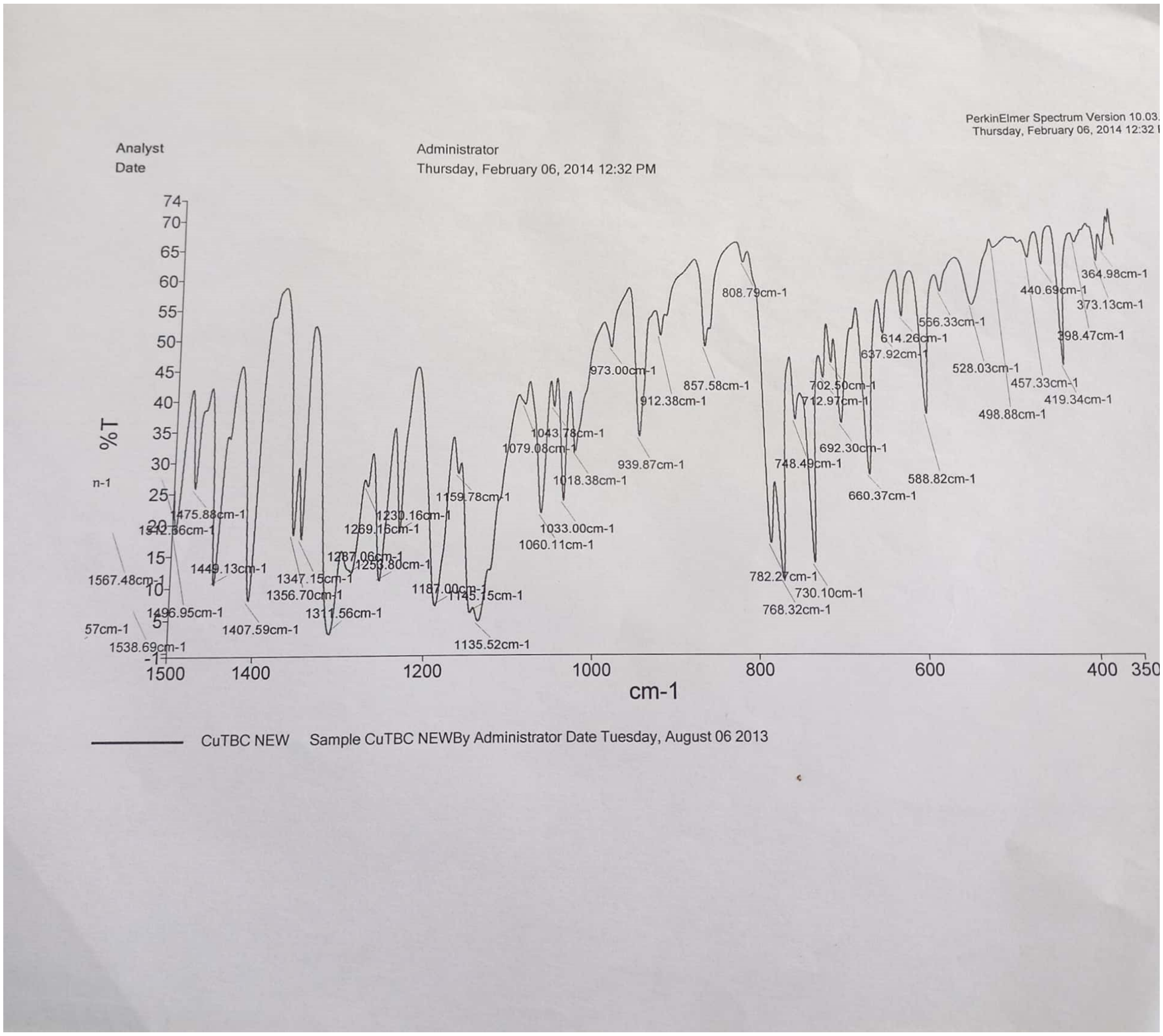

PerkinElmer Spectrum Version 10.03.
Thursday, February 06, 2014 12:32
Analyst
Date
Administrator
Thursday, February 06, 2014 12:32 PM
%T
cm-1
1567.48cm-1
1538.69cm-1
1512.36cm-1
1496.95cm-1
1475.88cm-1
1449.13cm-1
1407.59cm-1
1356.70cm-1
1347.15cm-1
1311.56cm-1
1269.15cm-1
1267.06cm-1
1253.80cm-1
1230.16cm-1
1187.00cm-1
1159.78cm-1
1145.15cm-1
1135.52cm-1
1079.08cm-1
1060.11cm-1
1043.78cm-1
1033.00cm-1
1018.38cm-1
973.00cm-1
939.87cm-1
912.38cm-1
857.58cm-1
808.79cm-1
782.27cm-1
768.32cm-1
748.49cm-1
730.10cm-1
712.97cm-1
702.50cm-1
692.30cm-1
660.37cm-1
637.92cm-1
614.26cm-1
588.82cm-1
566.33cm-1
528.03cm-1
498.88cm-1
457.33cm-1
440.69cm-1
419.34cm-1
398.47cm-1
373.13cm-1
364.98cm-1
CuTBC NEW
Sample CuTBC NEWBy Administrator Date Tuesday, August 06 2013

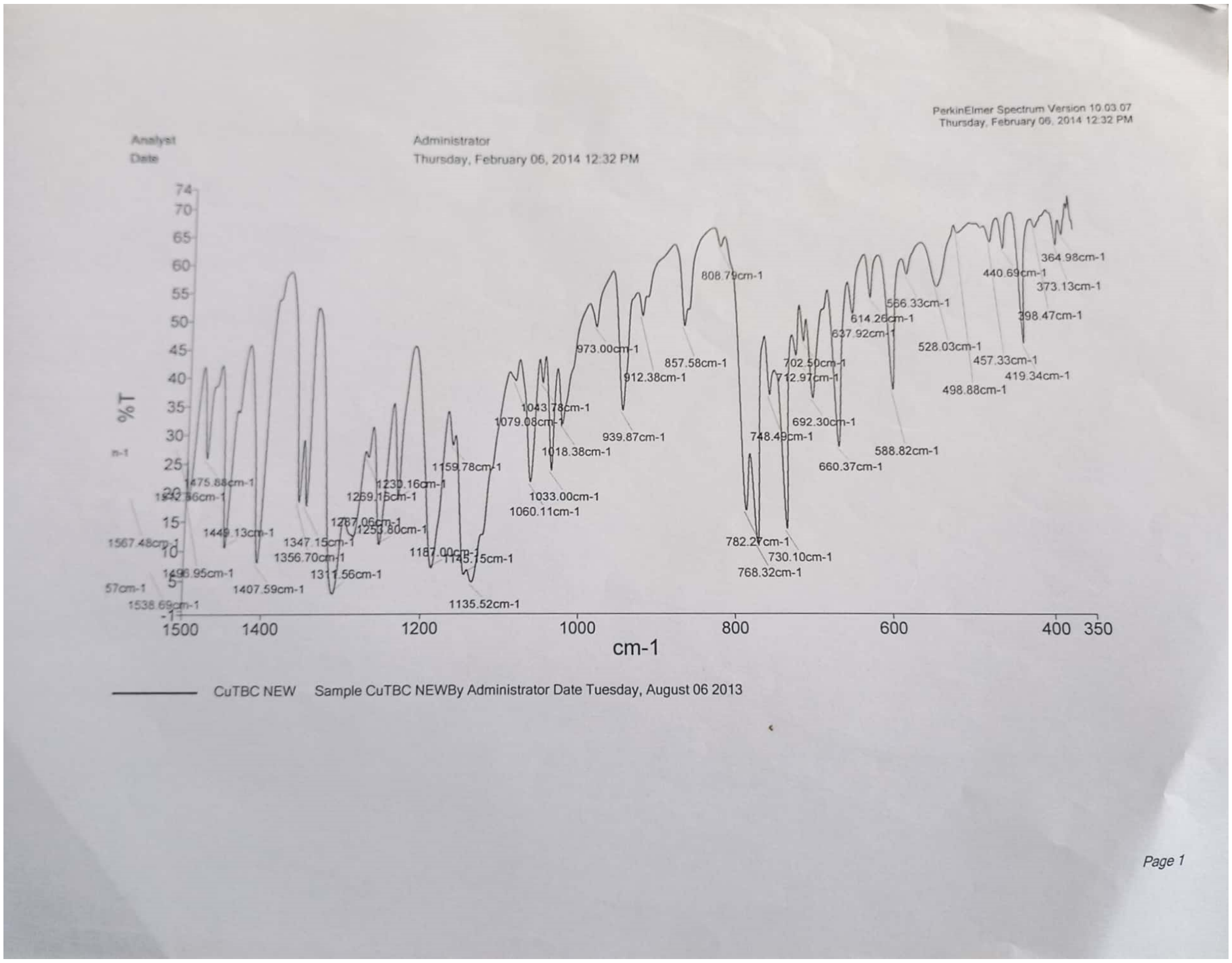

PerkinElmer Spectrum Version 10.03.07
Thursday, February 06, 2014 12:32 PM
Analyst
Date
Administrator
Thursday, February 06, 2014 12:32 PM
%T
cm-1
CuTBC NEW
Sample CuTBC NEWBy Administrator Date Tuesday, August 06 2013
Page 1
1475.88cm-1
1449.13cm-1
1567.48cm-1
1538.69cm-1
1407.59cm-1
1347.15cm-1
1356.70cm-1
1311.56cm-1
1230.16cm-1
1159.78cm-1
1187.00cm-1
1135.52cm-1
1079.08cm-1
1043.78cm-1
1060.11cm-1
1033.00cm-1
1018.38cm-1
973.00cm-1
939.87cm-1
912.38cm-1
857.58cm-1
808.79cm-1
782.27cm-1
768.32cm-1
748.49cm-1
730.10cm-1
712.97cm-1
702.50cm-1
692.30cm-1
660.37cm-1
637.92cm-1
614.26cm-1
588.82cm-1
566.33cm-1
528.03cm-1
498.88cm-1
457.33cm-1
440.69cm-1
419.34cm-1
398.47cm-1
373.13cm-1
364.98cm-1

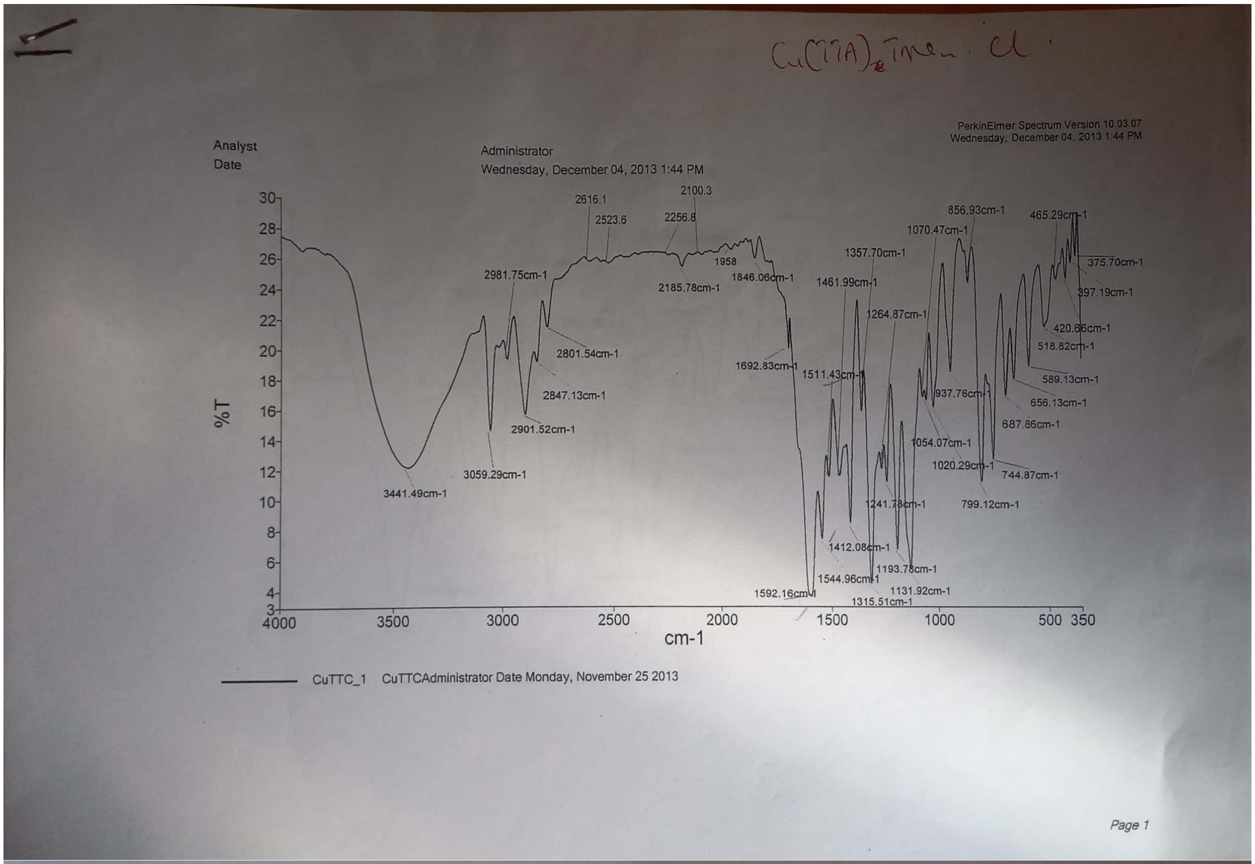
Cu(TTA)2Tmen. Cl
Analyst
Date
Administrator
Wednesday, December 04, 2013 1:44 PM
PerkinElmer Spectrum Version 10.03.07
Wednesday, December 04, 2013 1:44 PM
%T
cm-1
3441.49cm-1
3059.29cm-1
2981.75cm-1
2901.52cm-1
2847.13cm-1
2801.54cm-1
2616.1
2523.6
2256.8
2185.78cm-1
2100.3
1958
1846.06cm-1
1692.83cm-1
1592.16cm-1
1544.96cm-1
1511.43cm-1
1461.99cm-1
1412.08cm-1
1357.70cm-1
1315.51cm-1
1264.87cm-1
1241.78cm-1
1193.78cm-1
1131.92cm-1
1070.47cm-1
1054.07cm-1
1020.29cm-1
937.76cm-1
856.93cm-1
799.12cm-1
744.87cm-1
687.86cm-1
656.13cm-1
589.13cm-1
518.82cm-1
465.29cm-1
420.66cm-1
397.19cm-1
375.70cm-1
CuTTC_1 CuTTCAdministrator Date Monday, November 25 2013
Page 1

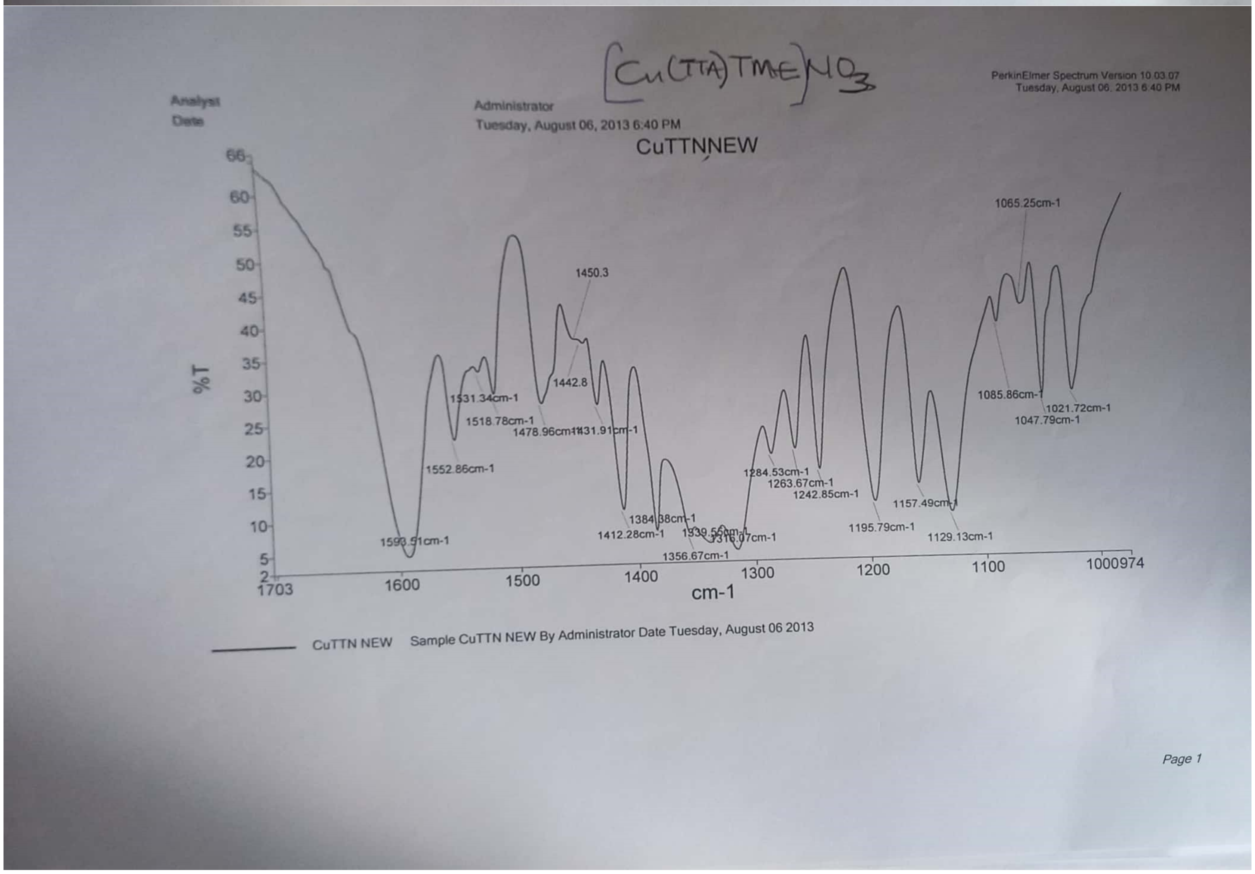
[Cu(TTA)TME]NO3
Analyst
Date
Administrator
Tuesday, August 06, 2013 6:40 PM
CuTTNNEW
PerkinElmer Spectrum Version 10.03.07
Tuesday, August 06, 2013 6:40 PM
%T
cm-1
1593.51cm-1
1552.86cm-1
1531.34cm-1
1518.78cm-1
1478.96cm-1
1450.3
1442.8
1431.91cm-1
1412.28cm-1
1384.38cm-1
1356.67cm-1
1284.53cm-1
1263.67cm-1
1242.85cm-1
1195.79cm-1
1157.49cm-1
1129.13cm-1
1085.86cm-1
1065.25cm-1
1047.79cm-1
1021.72cm-1
CuTTN NEW Sample CuTTN NEW By Administrator Date Tuesday, August 06 2013
Page 1

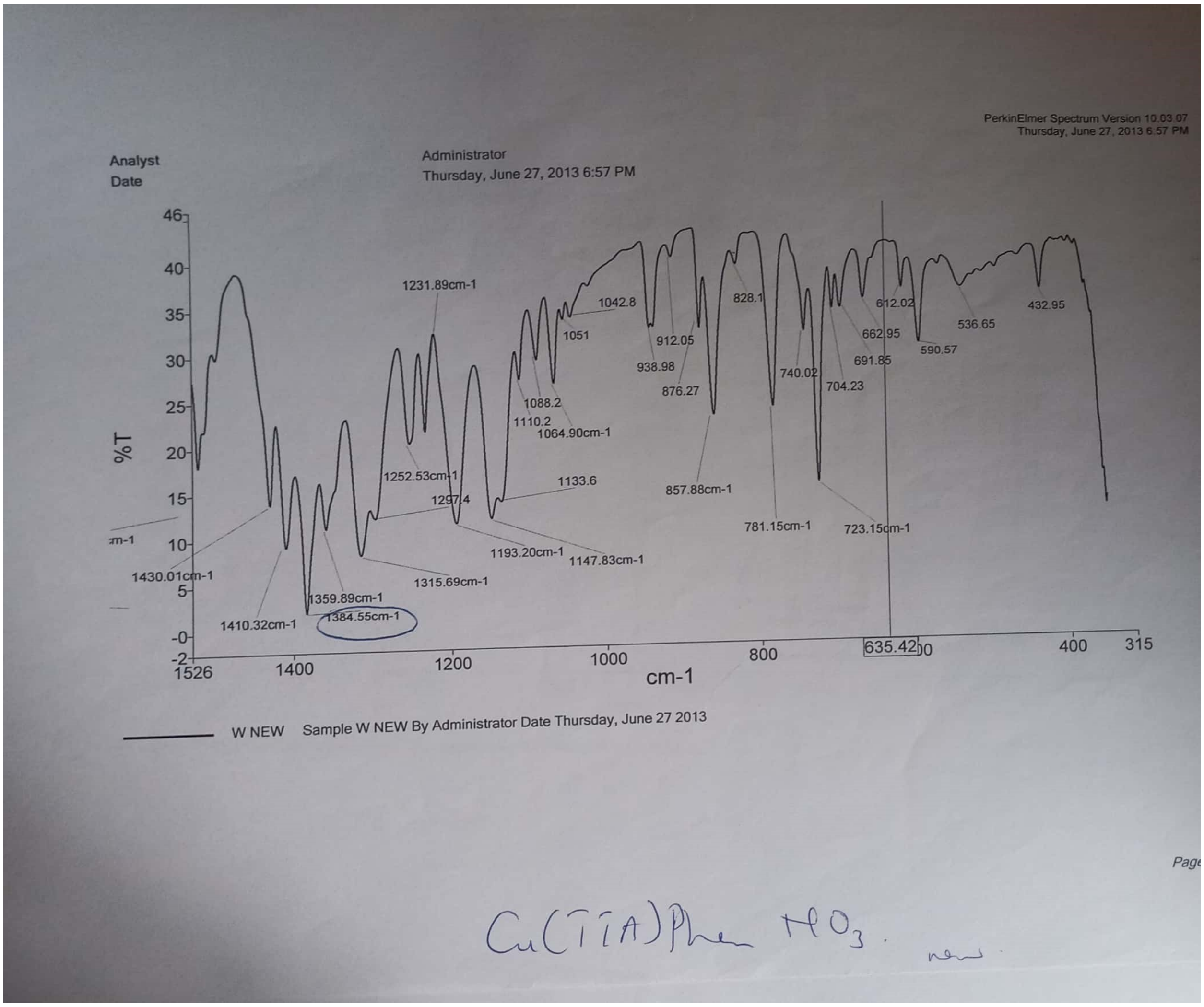
PerkinElmer Spectrum Version 10.03.07
Thursday, June 27, 2013 6:57 PM
Analyst
Date
Administrator
Thursday, June 27, 2013 6:57 PM
%T
cm-1
1231.89cm-1
1042.8
828.1
612.02
432.95
1051
912.05
662.95
536.65
938.98
691.85
590.57
740.02
876.27
704.23
1088.2
1110.2
1064.90cm-1
1252.53cm-1
1133.6
857.88cm-1
1297.4
781.15cm-1
723.15cm-1
1193.20cm-1
1147.83cm-1
1430.01cm-1
1315.69cm-1
1359.89cm-1
1384.55cm-1
1410.32cm-1
635.42
1526
1400
1200
1000
800
400
315
W NEW
Sample W NEW By Administrator Date Thursday, June 27 2013

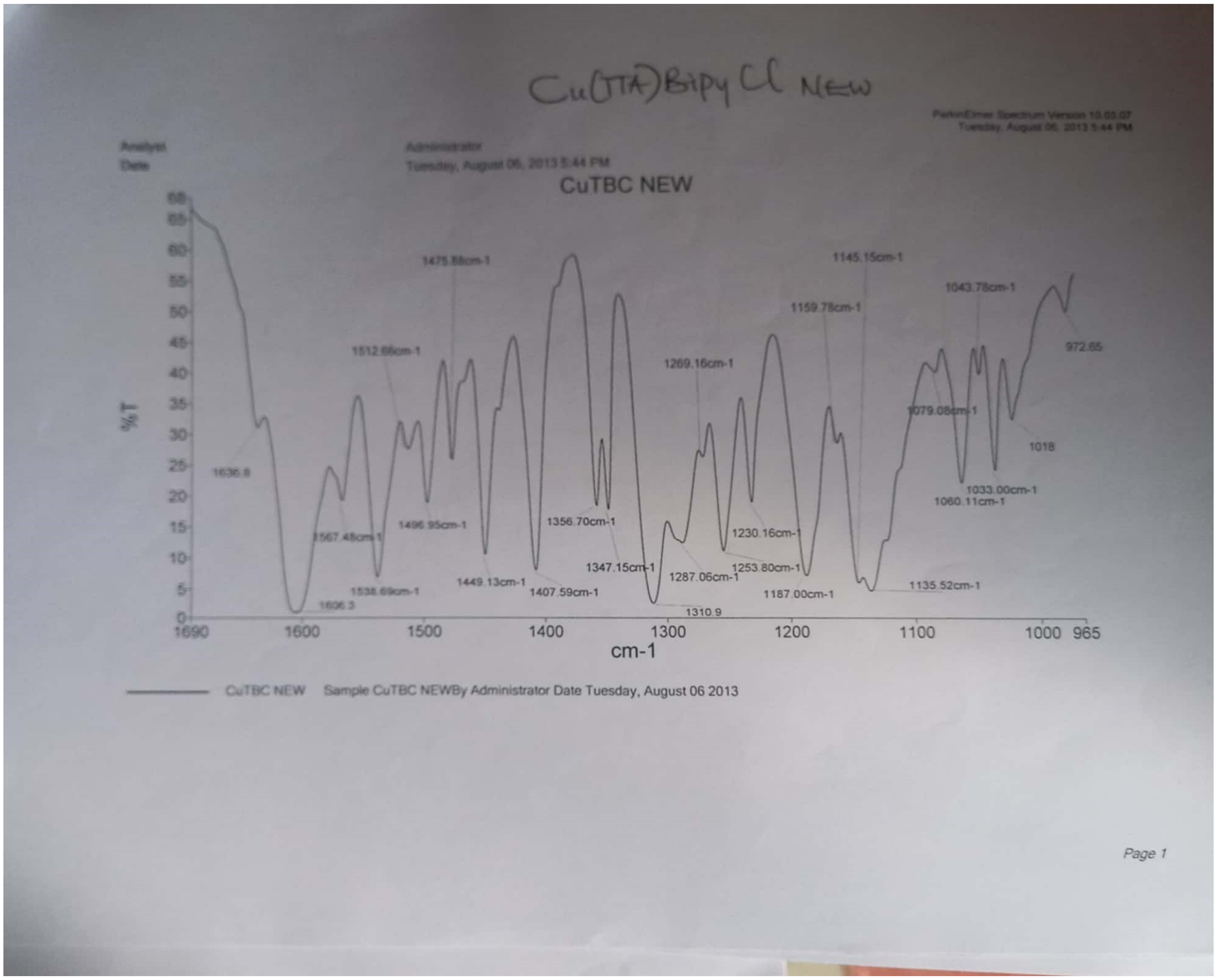

Cu(TTA)Bipy Cl New
CuTBC NEW
%T
cm-1
1636.8
1606.3
1567.48cm-1
1538.69cm-1
1512.66cm-1
1496.95cm-1
1475.88cm-1
1449.13cm-1
1407.59cm-1
1356.70cm-1
1347.15cm-1
1310.9
1287.06cm-1
1269.16cm-1
1253.80cm-1
1230.16cm-1
1187.00cm-1
1159.78cm-1
1145.15cm-1
1135.52cm-1
1079.08cm-1
1060.11cm-1
1043.78cm-1
1033.00cm-1
1018
972.65
CuTBC NEW Sample CuTBC NEWBy Administrator Date Tuesday, August 06 2013
Page 1

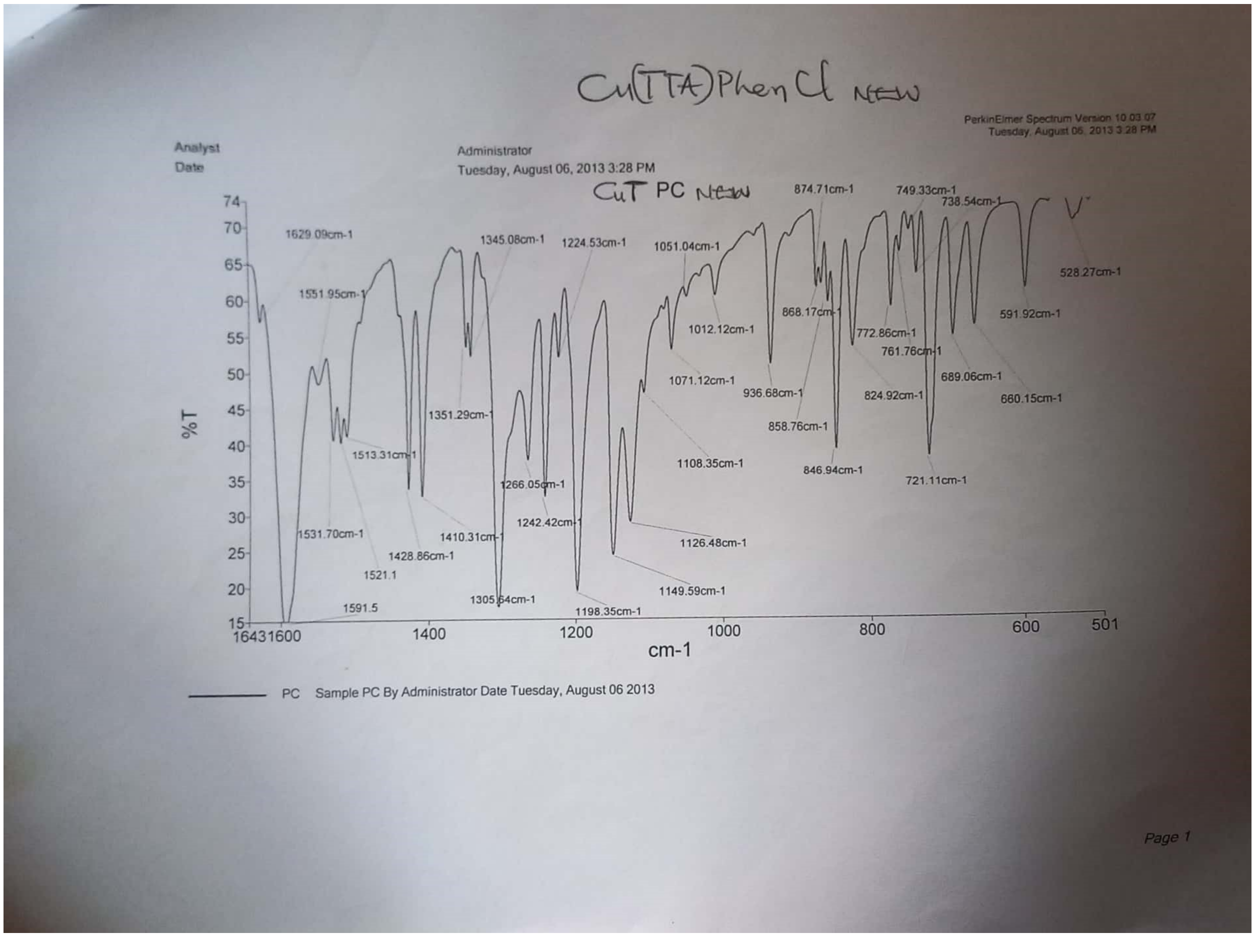

Cu(TTA)Phen Cl NEW
PerkinElmer Spectrum Version 10.03.07
Tuesday, August 06, 2013 3:28 PM
Analyst
Date
Administrator
Tuesday, August 06, 2013 3:28 PM
CuT PC NEW
%T
cm-1
1629.09cm-1
1551.95cm-1
1513.31cm-1
1531.70cm-1
1521.1
1591.5
1428.86cm-1
1410.31cm-1
1351.29cm-1
1345.08cm-1
1305.64cm-1
1266.05cm-1
1242.42cm-1
1224.53cm-1
1198.35cm-1
1149.59cm-1
1126.48cm-1
1108.35cm-1
1071.12cm-1
1051.04cm-1
1012.12cm-1
936.68cm-1
874.71cm-1
868.17cm-1
858.76cm-1
846.94cm-1
824.92cm-1
772.86cm-1
761.76cm-1
749.33cm-1
738.54cm-1
721.11cm-1
689.06cm-1
660.15cm-1
591.92cm-1
528.27cm-1
PC Sample PC By Administrator Date Tuesday, August 06 2013
Page 1

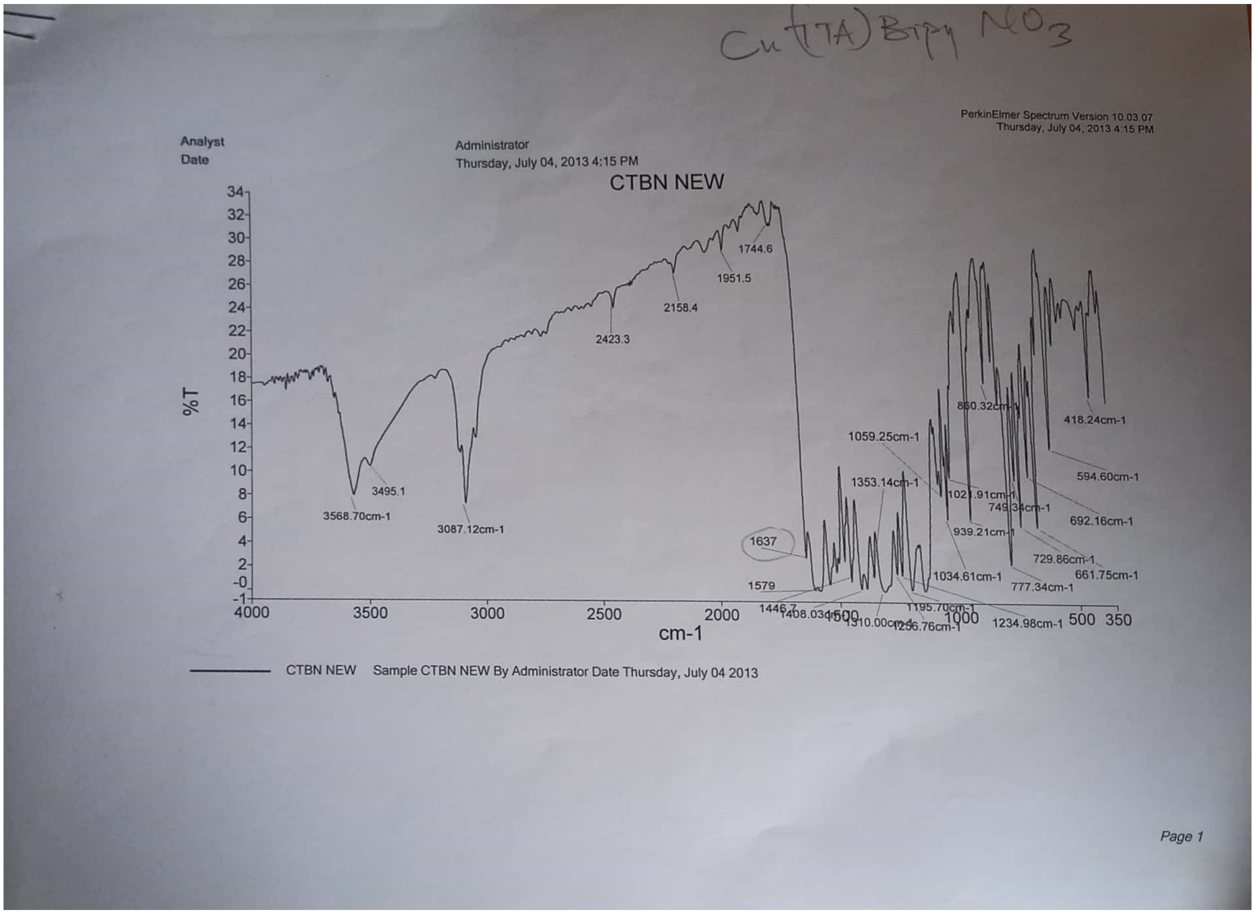
Cu(ITA)Bipy NO3
PerkinElmer Spectrum Version 10.03.07
Thursday, July 04, 2013 4:15 PM
Analyst
Date
Administrator
Thursday, July 04, 2013 4:15 PM
CTBN NEW
%T
cm-1
3568.70cm-1
3495.1
3087.12cm-1
2423.3
2158.4
1951.5
1744.6
1637
1579
1446.7
1408.03
1353.14cm-1
1310.00cm-1
1256.76cm-1
1234.98cm-1
1195.70cm-1
1059.25cm-1
1034.61cm-1
1021.91cm-1
939.21cm-1
860.32cm-1
777.34cm-1
749.34cm-1
729.86cm-1
692.16cm-1
661.75cm-1
594.60cm-1
418.24cm-1
CTBN NEW Sample CTBN NEW By Administrator Date Thursday, July 04 2013
Page 1
